# Supplementary material for: Remote versus face-to-face neuropsychological testing for dementia research: a comparative study in people with Alzheimer’s disease, frontotemporal dementia and healthy older individuals
Source: BMJ Open. 2022 Nov 25;12(11):e064576. doi: 10.1136/bmjopen-2022-064576 (PMC9702828; doi:10.1136/bmjopen-2022-064576)
Supplement: Supplementary data [file bmjopen-2022-064576supp001.pdf]

Supplementary Material: Remote versus face-to-face neuropsychological testing for dementia research: a comparative study in people with Alzheimer's disease, frontotemporal dementia and healthy older individuals

by MC Requena-Komuro, J Jiang et al

Technical aspects of the set-up for remote testing

Participants were permitted to use their preferred device (tablet, laptop, or desktop computer; see Table 1). To ensure screen visibility, we did not accept the use of smartphones. Most participants (90%) listened via speakers; six participants used headphones, and device volume was set to a comfortable level by each participant or their caregiver. Remote assessments were scheduled to ensure that testing could be completed in a quiet environment with minimal distractions. Additionally, the experimenters asked that each participant's video remained turned on so the experimenter could keep track of any distractions that may be occurring (see Table 1), as well as to ensure that no additional materials were used during the tests (e.g., paper/calculator). Where a task required visual presentation, this was done by screen sharing the Microsoft Powerpoint presentation containing the scanned stimuli for that task in full screen mode. Each patient's primary caregiver was asked to be available during each research session in case of any problems with using the equipment; in practice, no major technological issues arose. The primary caregiver was also permitted to be within the room during the sessions (see Table 1), but were told explicitly not to interfere with any of the neuropsychological and neurolinguistics tests given to the patient participants.

To check basic audibility in the remote testing environment, before each remote session, participants first listened to a set of 10 sentences from the Bamford-Kowal-Bench (BKB) list[1]. These sentences have previously been validated in hearing-impaired children. The spoken sentences were delivered online using an online experiment builder, Labvanced[2]. In each trial, a spoken sentence was played to the participant via screen and sound share on Zoom and the participant was encouraged to select the last word in the sentence they had just heard from three possible options presented visually via screen share (see Figure S1). A perfect score on the final three items was required for the participant to proceed to the remote testing session proper (this allowed each participant and/or caregiver to manually adjust the volume to a comfortable level during the first seven sentences). Most participants (95%) performed at ceiling across all ten items, and no participant made an error on any of the final three items, meaning that none was rejected based on their BKB performance (see Table S5). The order of sentences was fixed across participants.

Reasons for declining participation

Of those who did not take part, 20 (23.0%) did not respond (four AD, two bvFTD, three SD, two PNFA, three LPA, six controls); six (6.9%) declined due to not being comfortable using videoconferencing technology (two LPA, four controls); two (2.3%) declined due to changing health conditions (one AD, one control); one (1.1%) bvFTD patient declined due to sensory difficulties; one (1.1%) LPA patient declined due to family reasons; two (2.3%) declined due to anxieties about research (one AD, one LPA); three (3.4%) declined due to being too impaired

Requena-Komuro M-C, *et al. BMJ Open* 2022; 12:e064576. doi: 10.1136/bmjopen-2022-064576

(two bvFTD, one LPA); two (2.3%) declined due to being too busy with other activities (one SD, one PNFA); 12 (13.8%) provisionally said they were interested but we were unable to schedule a convenient time for them to take part (eight AD, three PNFA, one LPA); two (2.3%) healthy controls expressed an interest in participating but had not previously undertaken face-to-face research so were not included here; and one (1.1%) patient with atypical PPA expressed an interest in participating but was not included here as no other atypical PPA patients were recruited for the remote research.

Technical issues with remote test delivery

Three minor interruptions were recorded during administration of the remote general neuropsychological battery. On two occasions, the experimenter observed that the internet connection had slowed considerably for a brief period of time; however, this was not explicitly commented on by either participant affected (one healthy control, one bvFTD patient). The third interruption was a dog barking during an AD patient's testing session. In all cases, the trial that the interruption had occurred in was restarted, and the examiner judged that there was no penalty or benefit afforded by the interruption in any case. No interruptions were recorded during administration of the neurolinguistic battery, and no other interruptions were reported by participants.

Table S1. List of general neuropsychological and neurolinguistic tests delivered face-to-face that were not included in the remote battery.

Test	Reason for removal
WASI Vocabulary	Other tests from same domain already included
WASI Similarities	Other tests from same domain already included
Long Recognition Memory Test for Faces	To reduce the length of the remote battery, this was replaced with the Short Recognition Memory Test for Faces
Long Recognition Memory Test for Words	To reduce length of battery
Camden Paired Associates Learning	Other tests from same domain already included
WASI Block Design	Not feasible online as participants would not have blocks to manipulate
Stroop task	Not feasible online as too difficult for the examiner to determine the participant's target and therefore whether or not they had made an error. Also differences in colour display across participants' screens.
Trails A and B	Not feasible online as requires use of pencil and paper
WAIS-R Digit Symbol	Not feasible online as requires use of pencil and paper
Usual/Unusual views	Other tests from same domain already included
Modified Kissing and Dancing	Other tests from same domain already included
Baxter Spelling Test	Not feasible online as face-to-face task requires the participant to use pencil and paper; typing responses was not considered appropriate due to potential interference from spell-check software
Written sentences	Not feasible online as face-to-face task requires the participant to use pencil and paper; typing responses was not considered appropriate due to potential interference from spell-check software
Spatial Span Forwards	To reduce length of battery
Spatial Span Backwards	To reduce length of battery

We reduced the number of tests used in our face-to-face general neuropsychological and neurolinguistic batteries down for remote testing, reflecting a) the need to make the remote testing batteries shorter to minimise fatigue; b) impracticalities of administering certain stimuli remotely; and c) inability to adequately record participants' responses to some tasks. The Table shows the tasks that were not included in the remote batteries.

Requena-Komuro M-C, *et al. BMJ Open* 2022; 12:e064576. doi: 10.1136/bmjopen-2022-064576

Table S2. Bayesian statistics comparing general neuropsychological test performance on remote vs face-to-face assessments

Test	CTL	All Patients	AD	bvFTD	SD	PNFA	LPA
General intellect							
WASI Matrix	2.56[t] (Anecdotal)	5.403 (Substantial)	3.134[t] (Substantial)	1.477[t] (Anecdotal)	1.911[t] (Anecdotal)	1.356[t] (Anecdotal)	1.441[t] (Anecdotal)
Episodic memory							
RMT Faces short	NA	3.46 (Substantial)	0.411[t] (Anecdotal)	NA	NA	NA	NA
Working memory							
DS (Reverse)	4.304[t] (Substantial)	5.28 (Substantial)	2.746[t] (Anecdotal)	Variance at 0	1.991[t] (Anecdotal)	2.659 (Anecdotal)	0.417[t] (Anecdotal)
Short-term verbal memory							
DS (Forward)	2.663[t] (Anecdotal)	4.54 [t] (Substantial)	3.467[t] (Substantial)	1.948[t] (Anecdotal)	0.799[t] (Anecdotal)	2.528[t] (Anecdotal)	1.598[t] (Anecdotal)
Language							
BPVS	4.304[t] (Substantial)	4.05 (Substantial)	3.363 (Substantial)	1.836 (Anecdotal)	1.382[t] (Anecdotal)	2.754 (Anecdotal)	1.261 (Anecdotal)
GNT	2.923[t] (Anecdotal)	4.16 (Substantial)	3.542 (Substantial)	1.407[t] (Anecdotal)	2.331 (Anecdotal)	1.68[t] (Anecdotal)	1.325[t] (Anecdotal)
NART	2.83[t] (Anecdotal)	1.99 (Anecdotal)	1.552 (Anecdotal)	1.511[t] (Anecdotal)	2.606[t] (Anecdotal)	2.52[t] (Anecdotal)	1.948[t] (Anecdotal)
Category fluency	3.767[t] (Substantial)	1.08 (Anecdotal)	3.532 (Substantial)	2.186[t] (Anecdotal)	1.715[t] (Anecdotal)	1.027 (Anecdotal)	1.29[t] (Anecdotal)
Arithmetic							
GDA Total	3.671 (Substantial)	5.30 (Substantial)	3.33 (Substantial)	2.467[t] (Anecdotal)	0.943[t] (Anecdotal)	2.483[t] (Anecdotal)	1.123[t] (Anecdotal)
Visuospatial							
VOSP	0.0404 (Substantial)	4.76 (Substantial)	0.171[t] (Substantial)	2.215 (Anecdotal)	2.478 (Anecdotal)	2.553 (Anecdotal)	2.592[t] (Anecdotal)
Executive							

Requena-Komuro M-C, *et al. BMJ Open* 2022; 12:e064576. doi: 10.1136/bmjopen-2022-064576

Letter fluency	3.159^t (Substantial)	0.59 (Anecdotal)	2.86^t (Anecdotal)	2.426^t (Anecdotal)	1.519^t (Anecdotal)	0.511^t (Anecdotal)	0.188^t (Substantial)

A Bayes factor (BF_{01}) indicates the extent to which the null hypothesis is favoured against the alternative hypothesis (e.g., a BF_{01} value of 4 means that the obtained data are 4 times more likely under the null hypothesis than under the alternative hypothesis). A $BF_{01} > 3$ is therefore considered as substantial evidence in support of the null hypothesis; while a BF_{01} of <1/3 is considered as substantial evidence in support of the alternative hypothesis. Any values in between are categorised as 'anecdotal' evidence, equivalent to a non-significant result in inferential statistics [3]. Results are influenced by the prior (more specifically the shape of the prior influences the strength of the evidence), which can be specified be default using a Cauchy distribution, as here; the Cauchy scale set here is 1.00. The superscript ^{t} indicates that a parametric Bayesian test was used; else the non-parametric Mann Whitney (with 1000 iterative samples) was employed. Blue shading indicates that the alternative hypothesis (H1, i.e. there was a difference in performance across the two environments) was favoured with substantial evidence; Green shading indicates that the null hypothesis (H0; i.e. there was no difference in performance across environments) was favoured with substantial evidence. AD, patient group with typical Alzheimer's disease; BPVS, British Picture Vocabulary Scale; bvFTD, patient group with behavioural variant frontotemporal dementia; CTL, healthy control group; DS, Digit Span; F2F, face-to-face; GDA, Graded Difficulty Arithmetic test; GNT, Graded Naming Test; LPA, patient group with logopenic progressive aphasia; Matrix, WASI Matrix Reasoning; NART, National Adult Reading Test; PNFA, patient group with progressive nonfluent aphasia; RMT, Recognition Memory Test; SD, patient group with semantic dementia; VOSP, Visual Object Space Perception battery.

BMJ Publishing Group Limited (BMJ) disclaims all liability and responsibility arising from any reliance placed on this supplemental material which has been supplied by the author(s)

Table S3. Bayesian statistics comparing neurolinguistic test performance on remote vs face-to-face assessments

Test	CTL	All Patients	SD	PNFA	LPA
Phoneme perception					
PALPA 3	1.003 (Anecdotal)	2.918 (Anecdotal)	1.594 (Anecdotal)	2.731 (Anecdotal)	2.079 (Anecdotal)
Reading					
Non word reading	1.544 (Anecdotal)	3.239 (Substantial)	1.940^{t} (Anecdotal)	2.033^{t} (Anecdotal)	2.428^{t} (Anecdotal)
Regular reading	Variance at 0	2.989 (Anecdotal)	1.975 (Anecdotal)	2.305 (Anecdotal)	1.923 (Anecdotal)
Irregular reading	Variance at 0	2.996 (Anecdotal)	2.256 (Anecdotal)	2.194^{t} (Anecdotal)	2.005 (Anecdotal)
Naming					
BNT	3.612^{t} (Substantial)	1.995 (Anecdotal)	0.584^{t} (Anecdotal)	2.580 (Anecdotal)	1.490 (Anecdotal)
Semantic association					
Camel and cactus	4.039^{t} (Substantial)	1.067^{t} (Anecdotal)	1.524^{t} (Anecdotal)	2.102 (Anecdotal)	N<2 for F2F
Word comprehension					
Concrete synonyms	2.739^{t} (Anecdotal)	3.622 (Substantial)	2.009^{t} (Anecdotal)	2.400^{t} (Anecdotal)	1.413^{t} (Anecdotal)
Abstract synonyms	1.726^{t} (Anecdotal)	2.718 (Anecdotal)	0.782 (Anecdotal)	2.741 (Anecdotal)	1. 941 (Anecdotal)
Sentence comprehension					
PALPA55	0.944 (Anecdotal)	3.954 (Substantial)	2.246 (Anecdotal)	2.603 (Anecdotal)	2.110 (Anecdotal)
Speech repetition					
Monosyllabic word repetition	0.0487^{t} (Substantial)	0.117 (Anecdotal)	0.477^{t} (Anecdotal)	1.167^{t} (Anecdotal)	1.055^{t} (Anecdotal)
Bisyllabic word repetition	4.304^{t} (Substantial)	3.744 (Substantial)	1.086 (Anecdotal)	2.639 (Anecdotal)	0.650^{t} (Anecdotal)
Trisyllabic word repetition	Variance at 0	3.701 (Substantial)	1.339 (Anecdotal)	2.400 (Anecdotal)	1.677 (Anecdotal)
Graded difficulty sentence repetition	1.779 (Anecdotal)	2.983 (Anecdotal)	1.420^{t} (Anecdotal)	2.535^{t} (Anecdotal)	1.996 (Anecdotal)
Sentence construction					
Spoken	Variance at 0	1.525 (Anecdotal)	2.270 (Anecdotal)	1.960. (Anecdotal)	0.930^{t} (Anecdotal)

A Bayes factor (BF_{01}) is shown for each remote vs face-to-face testing comparison. The interpretation and colour coding are as indicated in the legend to Table S2 above. BNT, Boston Naming Test; CTRL, healthy control group; F2F, face-to-face; LPA, patient group with logopenic progressive aphasia; N, number of participants per group; PALPA, Psycholinguistic Assessment of Language Processing in Aphasia subtests; PNFA, patient group with progressive nonfluent aphasia; SD, patient group with semantic dementia

Table S4. Equality of variance analyses

Test	Healthy controls	Combined patients
**General neuropsychology tasks**		
WASI Matrix	F = 0.50, p = 0.32	F* = 0.15, p = 0.70
RMT faces short	N/A	F*=0.67, p=0.42
Digit span forwards	F = 1, p = 1	F = 1.36, p = 0.41
Digit span backwards	F = 1, p = 1	F* = 0.20, p = 0.65
BPVS	F = 0.82, p = 0.77	F* = 2.56, p = 0.11
GNT	F = 1.38, p =0.64	F* = 1.15, p = 0. 29
GDA	F* = 1.14, p = 0.30	F* = 0.13, p = 0.72
VOSP	F* = 0.42, p = 0.52	F* = 0.001, p = 0.97
NART	F = 1.15, p = 0.84	F* = 1.72, p = 0.19
Letter fluency	F = 2.20, p = 0.26	F* = 1.28, p = 0.26
Category fluency	F = 2.31, p = 0.23	F* = 0.96, p = 0.33
**Neurolinguistic tasks**		
PALPA-3	F* = 0.72, p = 0.41	F* = 0.07, p = 0.79
Nonword reading	F* = 3.08, p = 0.10	F* = 1.31, p = 0.26
Regular word reading	Variance=0	F* = 0.70, p = 0.41
Irregular word reading	Variance=0	F* = 0.30, p = 0.59
BNT	F = 0.57, p = 0.42	F* = 1.37, p = 0.25
Mono-syllabic repetition	**F = 0.11, p < 0.05**	**F* = 4.89, p = 0.03**
Bi-syllabic repetition	F = 0.58, p = 0.43	F* = 0.001, p = 0.98
Tri-syllabic repetition	Variance=0	F* = 0.04, p = 0.85
Concrete synonyms	F = 1.19, p = 0.80	F* = 0.91, p = 0.35
Abstract synonyms	F = 0.29, p = 0.09	F* = 2.24, p = 0.15
PALPA-55	F* = 4.41, p=0.05	F* = 1.10, p = 0.30
Sentence repetition	Variance=0	F* = 0.48, p = 0.49
Camel and cactus	F* = 1.30, p = 0.70	**F = 4.25, p = 0.02**
Spoken sentences	Variance=0	**F* = 4.37, p = 0.046**

Results of equality of variance analyses. F-test results are reported; *indicates that Levene's test was adopted instead due to violations of the assumption of normality. **Bold** indicates that the test was significant, meaning that the assumption of homogeneity of variance was violated. The short version of the RMT faces task was not administered to healthy control participants as part of their remote testing battery, making an equality of variance test impossible here. BPVS, British Picture Vocabulary Scale; GDA, Graded Difficulty Arithmetic test; GNT, Graded Naming Test; Matrix, WASI Matrix Reasoning; NART, National Adult Reading Test; VOSP, Visual Object Space Perception Object Decision task. BNT, Boston Naming Test; PALPA, Psycholinguistic Assessment of Language Processing in Aphasia.

Requena-Komuro M-C, *et al. BMJ Open* 2022; 12:e064576. doi: 10.1136/bmjopen-2022-064576

Table S5. Performance on audibility screening task by remote participant groups

	CTL	AD	bvFTD	SD	PNFA	LPA
Pre neuropsychology battery						
Average number of incorrect items (/10)	0.0 (0.0)	0.1 (0.4)	0.0 (0.0)	0.0 (0.0)	0.2 (0.4)	0.0 (0.0)
Average number of errors on last three items (/3)	0.0 (0.0)	0.0 (0.0)	0.0 (0.0)	0.0 (0.0)	0.0 (0.0)	0.0 (0.0)
Pre neurolinguistic battery						
Average number of incorrect items (/10)	0.0 (0.0)	NA	NA	0.0 (0.0)	0.4 (0.9)	0.0 (0.0)
Average number of errors on last three items (/3)	0.0 (0.0)	0.0 (0.0)	0.0 (0.0)	0.0 (0.0)	0.0 (0.0)	0.0 (0.0)

The data indicate that there were no major background listening environmental confounds nor any significant differences between participant groups (all p>0.05). AD, patient group with typical Alzheimer's disease; bvFTD, patient group with behavioural variant frontotemporal dementia; CTL, healthy control group; LPA, patient group with logopenic progressive aphasia; NA, not applicable; PNFA, patient group with progressive non-Fluent aphasia; SD, patient group with semantic dementia.

Figure S1. Example of basic audibility screening measure

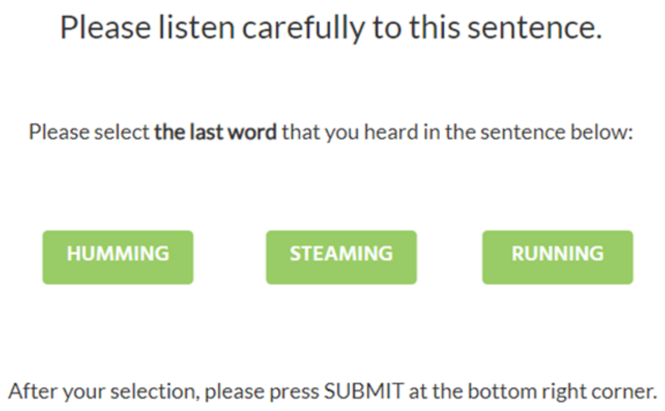

The Figure shows a Labvanced display of the BKB hearing screening measure. In this example, the sentence spoken was “The car engine is running”. For each sentence, two foils were displayed alonside the target, both of which made sense in the sentence when replacing the target. One of the foils was also selected to loosely rhyme with the target word (here, “humming”).

Figure S2. Performance profiles of patients on tasks in general neuropsychological battery.

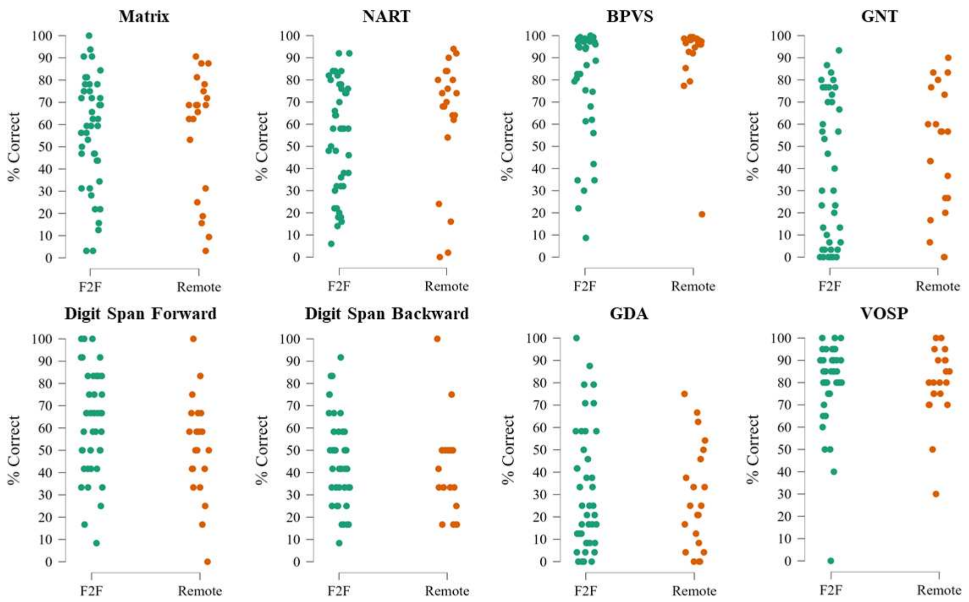

Scatter plots showing performance profiles of individual patients on tasks in the general neuropsychological battery. BPVS, British Picture Vocabulary Scale; GDA, Graded Difficulty Arithmetic test; GNT, Graded Naming Test; Matrix, WASI Matrix Reasoning; NART, National Adult Reading Test; VOSP, Visual Object Space Perception Object Decision task.

Figure S3. Performance profiles of patients on tasks in the neurolinguistic battery.

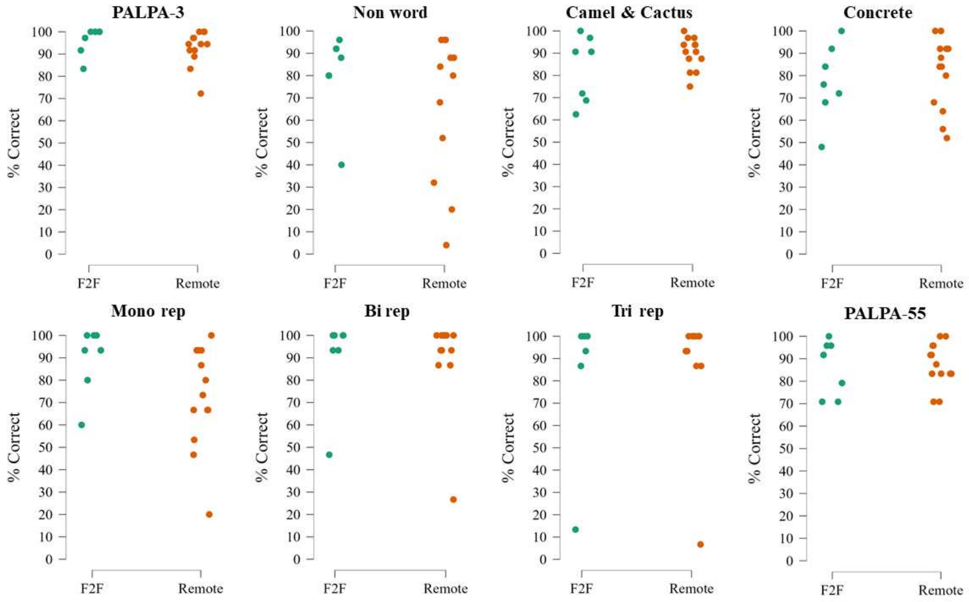

Scatter plots indicating percentage scores for each patient on representative tests from the neurolinguistic battery administered face-to-face and remotely. Bi rep, bisyllabic single word repetition; BNT, Boston Naming Test; Concrete, concrete synonyms test; F2F, face-to-face; Mono rep, monosyllabic single word repetition test; Non word, non-word reading test; PALPA, Psycholinguistic Assessment of Language Processing in Aphasia; Tri rep, trisyllabic single word repetition.

References

1. Bench J, Kowal Å, Bamford J. The Bkb (Bamford-Kowal-Bench) Sentence Lists for Partially-Hearing Children. *British Journal of Audiology* 1979;13(3):108-12. doi: 10.3109/03005367909078884
2. Finger H, Goeke C, Diekamp D, et al. LabVanced: A Unified JavaScript Framework for Online Studies. *International Conference on Computational Social Science* 2016
3. Ashworth M, Palikara O, Burchell E, et al. Online and Face-to-Face Performance on Two Cognitive Tasks in Children With Williams Syndrome. *Front Psychol* 2021;11:594465. doi: 10.3389/fpsyg.2020.594465
