# Supplementary material for: Remote versus face-to-face neuropsychological testing for dementia research: a comparative study in people with Alzheimer’s disease, frontotemporal dementia and healthy older individuals
Source: BMJ Open. 2022 Nov 25;12(11):e064576. doi: 10.1136/bmjopen-2022-064576 (PMC9702828; doi:10.1136/bmjopen-2022-064576)
Supplement: Author's manuscript [file bmjopen-2022-064576.draft_revisions.pdf]

BMJ Open is committed to open peer review. As part of this commitment we make the peer review history of every article we publish publicly available.

When an article is published we post the peer reviewers' comments and the authors' responses online. We also post the versions of the paper that were used during peer review. These are the versions that the peer review comments apply to.

The versions of the paper that follow are the versions that were submitted during the peer review process. They are not the versions of record or the final published versions. They should not be cited or distributed as the published version of this manuscript.

BMJ Open is an open access journal and the full, final, typeset and author-corrected version of record of the manuscript is available on our site with no access controls, subscription charges or pay-per-view fees (http://bmjopen.bmj.com).

If you have any questions on BMJ Open's open peer review process please email info.bmjopen@bmj.com

BMJ Open

Neuropsychological assessments for dementia research in the COVID-19 era: comparing remote and face-to-face testing

Journal:	*BMJ Open*
Manuscript ID	bmjopen-2022-064576
Article Type:	Original research
Date Submitted by the Author:	10-May-2022

Complete List of Authors:	Requena-Komuro, Mai-Carmen; University College London Dementia Research Centre,<br>Jiang, Jessica; University College London<br>Dobson, Lucianne; University College London<br>Benhamou, Elia; University College London; University of Cambridge<br>Russell, Lucy; University College London<br>Bond, Rebecca L; University College London<br>Brotherhood, Emilie V.; University College London<br>Greaves, Caroline; University College London<br>Barker, Suzie; University College London<br>Rohrer, Jonathan; UCL, Queen Square Institute of Neurology<br>Crutch, Sebastian; University College London Institute of Neurology<br>Warren, Jason; UCL<br>Hardy, Chris ; University College London, Dementia Research Centre

Keywords:	Dementia < NEUROLOGY, Telemedicine < BIOTECHNOLOGY & BIOINFORMATICS, Adult neurology < NEUROLOGY

SCHOLARONE™
Manuscripts

I, the Submitting Author has the right to grant and does grant on behalf of all authors of the Work (as defined in the below author licence), an exclusive licence and/or a non-exclusive licence for contributions from authors who are: i) UK Crown employees; ii) where BMJ has agreed a CC-BY licence shall apply, and/or iii) in accordance with the terms applicable for US Federal Government officers or employees acting as part of their official duties; on a worldwide, perpetual, irrevocable, royalty-free basis to BMJ Publishing Group Ltd ("BMJ") its licensees and where the relevant Journal is co-owned by BMJ to the co-owners of the Journal, to publish the Work in this journal and any other BMJ products and to exploit all rights, as set out in our licence.

The Submitting Author accepts and understands that any supply made under these terms is made by BMJ to the Submitting Author unless you are acting as an employee on behalf of your employer or a postgraduate student of an affiliated institution which is paying any applicable article publishing charge ("APC") for Open Access articles. Where the Submitting Author wishes to make the Work available on an Open Access basis (and intends to pay the relevant APC), the terms of reuse of such Open Access shall be governed by a Creative Commons licence – details of these licences and which Creative Commons licence will apply to this Work are set out in our licence referred to above.

Other than as permitted in any relevant BMJ Author's Self Archiving Policies, I confirm this Work has not been accepted for publication elsewhere, is not being considered for publication elsewhere and does not duplicate material already published. I confirm all authors consent to publication of this Work and authorise the granting of this licence.

For peer review only

Neuropsychological assessments for dementia research in the COVID-19 era: comparing remote and face-to-face testing

Maï-Carmen Requena-Komuro[1*], Jessica Jiang[1*], Lucianne Dobson[1], Elia Benhamou[1,2], Lucy Russell[1], Rebecca L Bond[1], Emilie V Brotherhood[1], Caroline Greaves[1], Suzie Barker[1], Jonathan D Rohrer[1], Sebastian J Crutch[1], Jason D Warren[1], Chris JD Hardy[1]

*joint lead authors

[1]Dementia Research Centre, Queen Square Institute of Neurology, University College London
[2]MRC Cognition and Brain Sciences Unit, University of Cambridge

Correspondence to: Dr Chris J D Hardy,
Dementia Research Centre
UCL Queen Square Institute of Neurology
1st Floor, 8-11 Queen Square
London WC1N 3AR
United Kingdom
Email: chris.hardy@ucl.ac.uk
Tel: 44 203 448 3676

Keywords: dementia, primary progressive aphasia, frontotemporal dementia, Alzheimer's disease, neuropsychology, remote, online,

Word count: Abstract: 268 words, Main text: 4108 words

For peer review only

For peer review only - http://bmjopen.bmj.com/site/about/guidelines.xhtml

Abstract

Objectives: We explored whether adapting traditional neuropsychological tests for online administration against the backdrop of COVID-19 was feasible for people with diverse forms of dementia and healthy older controls. We compared face-to-face and remote settings to ascertain whether remote administration affected performance.
Design: We used a longitudinal design for healthy older controls who completed face-to-face neuropsychological assessments between three and four years before taking part remotely. For patients, we used a cross-sectional design, contrasting a prospective remote cohort with a retrospective face-to-face cohort matched in age, education, and disease duration.
Setting: Remote assessments were performed using video-conferencing and online testing platforms, with participants using a personal computer or tablet and situated in a quiet room in their own home. Face-to-face assessments were carried out in dedicated testing rooms in our research centre.
Participants: The remote cohort comprised ten healthy older controls (also seen face-to-face 3-4 years previously) and 25 patients (n=8 Alzheimer's disease (AD); n=3 behavioural variant frontotemporal dementia (bvFTD); n=4 semantic dementia (SD); n=5 progressive nonfluent aphasia (PNFA); n=5 logopenic aphasia (LPA)). The face-to-face patient cohort comprised 64 patients (n=25 AD; n=12 bvFTD; n=9 SD; n=12 PNFA; n=6 LPA).
Primary and secondary outcome measures: The outcome measures comprised the strength of evidence under a Bayesian analytic framework for differences in performances between face-to-face and remote testing environments on a general neuropsychological (primary outcomes) and neurolingustic battery (secondary outcomes).
Results: There was evidence to suggest comparable performance across testing environments for all participant groups, for a range of neuropsychological tasks across both batteries.
Conclusions: Our findings suggest that remote delivery of neuropsychological tests for dementia research is feasible.

Strengths and limitations of this study

Methodological strengths of this study include:

- Diverse patient cohorts representing rare dementias with specific communication difficulties
- Sampling of diverse and relevant neuropsychological domains
- Use of Bayesian statistics to quantify the strength of evidence for the putative null hypothesis (no effect between remote and face-to-face testing)

Limitations include:

- Relatively small cohort sizes
- Lack of direct head-to-head comparisons of test environment in the same patients

For peer review only - http://bmjopen.bmj.com/site/about/guidelines.xhtml

1. Introduction

The COVID-19 pandemic and associated social distancing and lockdown measures imposed a series of daunting challenges for conducting research with people with dementia. In the UK, three national lockdowns between March 2020 and February 2021 largely prevented face-to-face research. People with dementia are at increased risk for COVID-19[1], and many participants understandably did not feel safe to travel for research, particularly before widespread vaccination was implemented. Here, we describe our attempts to translate our traditional neuropsychology and neurolinguistics batteries (typically administered face-to-face) for remote administration.

Development and implementation of online cognitive assessments for dementia patients, particularly within communities who experience difficulties in accessing clinical care is not new[2]. Telemedicine has been previously used successfully in Alzheimer's disease (AD)[3 4] and with rarer dementias such as primary progressive aphasia (PPA)[5-7] and behavioural variant frontotemporal dementia (bvFTD)[7]. However, due to COVID-19 there has been a more pervasive shift towards the use of online methods to meet clinical, support and research needs[8 9].

A review by Hunter and colleagues[10] summarises 20 years of research comparing face-to-face and online administration of cognitive tests in healthy older adults (≥40 years old) and participants diagnosed with mild cognitive impairment (MCI), AD, or other types of dementia (often unspecified). The authors identified 12 studies that used videoconferencing methods. Overall, there was clear evidence to suggest that remote cognitive testing for people living with AD and other forms of dementia is feasible. Additionally, there is evidence to suggest that online performance remains stable over time (with a maximum delay of three months between assessments), particularly for the domains of executive function, working memory, verbal episodic memory, and language. Minimal evidence was available for visuospatial tasks, and tests of single word and sentence comprehension.

Notwithstanding considerable progress in this area to date, further research into remote neuropsychological testing of patients with neurodegenerative diseases is required. There are three main issues that need addressing. First, it would be informative to understand more about the feasibility of remote testing in a non-controlled "home" environment (e.g., with potentially unstable internet connection and technological equipment not designed for research purposes). Second, it would be instructive to assess a range of patients with different forms of dementia, including PPA and bvFTD. Third, it is important to explore the feasibility of a range of neuropsychological tests measuring diverse cognitive functions remotely.

Based largely on the face-to-face protocol for general neuropsychological and neurolinguistic testing used at our research centre, we built a protocol for remote testing of patients diagnosed with typical AD, patients representing major variants of PPA (semantic dementia (SD), progressive nonfluent aphasia (PNFA), logopenic aphasia (LPA)), and bvFTD. Patients were tested from their homes via the widely used video conferencing software, Zoom (Zoom Video Communications Inc). We also recruited a small cohort of healthy older adults who had taken part in our face-to-face research at the Dementia Research Centre three-to-four years before the pandemic. Here, we compared the healthy controls' performance on several neuropsychological and neurolinguistic tests between the two testing environments (face-to-face vs remote). We also compared the performance of patients tested remotely with a historical

For peer review only - http://bmjopen.bmj.com/site/about/guidelines.xhtml

face-to-face cohort of patients chosen to represent the same syndromes and to match the remote cohort based on age, education, and symptom duration. We adopted a Bayesian approach that assesses the amount of evidence in favour of the null hypothesis (i.e., that there is no significant difference in performance on a given neuropsychological task between testing environments) relative to the alternative hypothesis (i.e., that there is a significant difference in performance on a given neuropsychological task between testing environments).

Following previous research[10], we did not predict major differences in terms of participants' performances when tested face-to-face and remotely on most neuropsychological and neurolinguistics tests. However, we did consider the potential for poorer performance on tests of speech perception that were administered remotely, given additional difficulties associated with controlling the remote auditory environment.

2. Methods

Participant recruitment and group matching

Recruitment for the study took place between February-August 2021. Potential patient participants were identified via the Specialist Cognitive Disorders Clinic at the National Hospital for Neurology and Neurosurgery, direct research referrals from external clinicians or via Rare Dementia Support (www.raredementiasupport.org); healthy controls were recruited via our research participant database.

An initial telephone screen was conducted for each participant to establish they had access to the necessary equipment (tablet or desktop/laptop computer), a broadband internet connection, a quiet testing space to support the remote research assessment, and no preclusive hearing or visual impairments. We also performed the telephone version of the Mini-Mental State Examination (MMSE) with patients to assess their disease severity[11 12]. A cut-off score of 12 on the T-MMSE (which corresponds to a converted MMSE score of 16) was used as an inclusion criterion[11]. Of the potential participants who were approached about research, six (two healthy controls, one right temporal variant FTD, one PNFA, two LPA) declined for reasons related to remote delivery (e.g., not being comfortable with videoconferencing technology).

Twenty-five patients (eight with typical AD, three bvFTD, four SD, five PNFA, five LPA) were recruited for the remote study. For comparison purposes, a reference historical cohort comprising 64 patients (25 with AD, 12 bvFTD, nine SD, 12 PNFA, six LPA) who had undertaken a face-to-face research assessment at our Centre between 2013 and 2020 were selected, matching the cohort assessed remotely as closely as possible for syndromic composition, age, years of education and symptom duration. Henceforth, these are referred to as the 'remote' and 'face-to-face' patient cohorts, respectively. All patients fulfilled consensus diagnostic criteria for the relevant syndromic diagnosis[13-15] and all had clinically mild-to-moderate severity disease. Where available, brain MRI was consistent with the syndromic diagnosis, without evidence of significant cerebrovascular burden. Ten healthy older individuals with no history of neurological or psychiatric illness and who had been seen for face-to-face testing between three and four years previously also underwent remote assessments. Demographic and clinical details for all participants are summarised in Table 1. All participants gave informed consent for their involvement in the study.

For peer review only - http://bmjopen.bmj.com/site/about/guidelines.xhtml

Table 1. General demographic and clinical characteristics for all participant groups: comparison of remote and face-to-face cohort characteristics.

Characteristic	CTL	AD		bvFTD		SD		PNFA		LPA	
	Remote	F2F	Remote	F2F	Remote	F2F	Remote	F2F	Remote	F2F	Remote
N	10	25	8	12	3	9	4	12	5	6	5
Gender (M/F)	7/3	15/10	5/3	8/4	3/0	7/2	2/2	6/6	2/3	4/2	4/1
Age (yrs)	74.0 (4.1)*	69.5 (4.1)	69.8 (5.9)	70.3 (2.9)	72.0 (5.6)	60.3 (5.2)	58.3 (9.3)	67.1 (5.2)	68.2 (6.4)	69.5 (3.6)	71.6 (4.7)
Education (yrs)	17.6 (0.7)	14.8 (2.5)	16.0 (3.7)	14.9 (2.7)	14.7 (3.1)	16.0 (1.9)	15.5 (3.1)	15.3 (2.3)	14.8 (2.8)	16.0 (1.7)	16.0 (3.3)
Handedness (R/L/A)	9/1/0	23/1/1	8/0/0	11/1/0	3/0/0	9/0/0	3/1/0	10/2/0	5/0/0	5/1/0	5/0/0
Symptom duration (yrs)	NA	6.8 (2.3)	7.3 (3.7)	4.7 (2.1)	3.7 (3.8)	4.1 (1.9)	3.5 (1.9)	3.4 (1.7)	3.2 (0.8)	4.2 (1.9)	3.8 (1.9)

Mean (standard deviation) values are shown for each group. No significant differences between remote and face-to-face cohorts were found. *on average healthy controls were 3.5 years younger when tested face-to-face; A, ambidextrous; AD, patient group with typical Alzheimer's disease; bvFTD, patient group with behavioural variant frontotemporal dementia; CTL, healthy control group; F, female; F2F, face-to-face; L, left; LPA, patient group with logopenic progressive aphasia; M, male; N, number of participants per group; NA, not applicable; PNFA, patient group with progressive non-fluent aphasia; R, right; SD, patient group with semantic dementia.

For peer review only

For peer review only - http://bmjopen.bmj.com/site/about/guidelines.xhtml

Testing procedure – face-to-face

Data for the reference historical cohort were collected under our face-to-face research assessment protocol, as delivered in experimental sessions at the Dementia Research Centre between 2013 and 2020. Under this protocol, all neuropsychological tests were administered in dedicated quiet testing rooms, with the participant sitting opposite the experimenter. Patients were predominantly tested on their own, unless the informant accompanying them to the study visit requested to be present and the participant agreed to this. In these cases, the informant was explicitly asked not to intervene during testing. No feedback was given on performance and no time limits were imposed (unless timing was intrinsic to the test). General neuropsychological and neurolinguistic test batteries (see Tables 2 and 3) were administered, following standard methods. The neurolinguistic battery was developed specifically to characterise the language profiles of people with PPA and therefore, was not administered to bvFTD or AD participants.

Modifying the face-to-face neuropsychological battery for remote delivery

We reviewed the general neuropsychological and neurolinguistic batteries that had been used historically at our Centre for face-to-face administration, in order to identify tests that could be feasibly delivered remotely online while preserving the overall structure of the batteries and sampling across cognitive domains as far as possible. In selecting tests, an important consideration was to sample cognitive domains representatively so that we could establish phenotypic profiles of deficits in the target neurodegenerative syndromes (see Table S1, Supplementary file). Where a task required visual stimulus presentation, a high-quality copy of the stimuli was made. Images were then imported into Microsoft Powerpoint for subsequent presentation to the participant via screen share.

Tests that were retained for remote testing (i.e., tests administered to both the remote and face-to-face patient cohorts) are itemised in Tables 2 and 3.The general neuropsychological battery comprised tests of general intellect (Wechsler Adult Intelligence Scale (WASI) Matrix Reasoning[16]), episodic memory (Recognition Memory Test (RMT) for Faces[17]), working memory (Digit span forwards[18]), language (the graded naming test (GNT[19]), British Picture Vocabulary Scale (BPVS[20]) and National Adult Reading Test (NART)[21]), arithmetic (Graded Difficulty Arithmetic (GDA)), visual perception (Visual Object and Space Perception (VOSP) Object Decision task[22]) and executive function (Digit span reverse[18], letter ('F') and category fluency ('animal') tasks[23]).

The neurolinguistic battery comprised tests of phoneme perception (a shortened version of the Psycholinguistic Assessments of Language Processing in Aphasia (PALPA) Test 3 – minimal pair discrimination[24]), reading (graded non-word reading[25], and graded tests of regular word and irregular word reading adapted from[26]), confrontation naming (a subset of items from the Boston Naming Test (BNT)[27]), semantic association (modified Camel and Cactus test[28]), single word comprehension (concrete and abstract synonyms[29]), sentence comprehension (a shortened version of the PALPA Subtest 55[24]), speech repetition (tests of monosyllabic, bisyllabic, and trisyllabic single word repetition[30], graded difficulty sentence repetition using a subset of items from the Sentence Repetition test[31] and sentence construction based on[32]. We opted to include two naming tasks as the GNT is part of the core neuropsychological battery at our Centre and therefore administered to patients with all diagnoses. However, as patients with SD often score at floor on this task[33], we also include a shortened version of the BNT in our

For peer review only - http://bmjopen.bmj.com/site/about/guidelines.xhtml

neurolinguistic battery that is administered to patients with PPA as SD patients are more likely to be above floor on this task[34].

For peer review only

For peer review only - http://bmjopen.bmj.com/site/about/guidelines.xhtml

Table 2. General neuropsychological performance for all participant groups: comparison of remote and face-to-face test administration.

**Test**	**CTL**		**AD**		**bvFTD**		**SD**		**PNFA**		**LPA**	
	**F2F**	**Remote**	**F2F**	**Remote**	**F2F**	**Remote**	**F2F**	**Remote**	**F2F**	**Remote**	**F2F**	**Remote**
**No. tested**	10	10	25	8	12	3	9	4	12	5	6	5
General intellect												
WASI Matrix (/32)	26.9 (2.4)	26.3 (3.5)	**14.5 (7.5)**	**13.7 (11.5)[b]**	14.6 (9.5)[a]	7.7 (2.5)	25.8 (4.9)	23.3 (3.4)	18.6 (8.3)[a]	24.0 (2.6)	10.0 (9.2)	17.6 (10.3)
Episodic memory												
RMT faces (Short) (/25)	NA	23.3 (2.5)	18.9 (3.7)[h]	15.3 (2.1)	NA	20.3 (4.0)	NA	19.3 (3.7)	NA	22.4 (3.7)	NA	21.6 (3.8)
RMT faces (/50)	42.7 (4.7)	NA	30.44 (6.56)[g]	NA	28.56 (6.29)[c]	NA	31.5 (4.47)[a]	NA	40.3 (5.79)[b]	NA	33.17 (8.93)	NA
Working memory												
DS (Forward) (/12)	9.8 (2.0)	9.2 (2.0)	**6.5 (2.1)[a]**	**6.6 (2.3)**	7.8 (2.9)	6.3 (0.6)	9.4 (2.2)	7.3 (0.5)	5.1 (2.6)[b]	6.0 (4.4)	3.0 (3.1)[a]	5.0 (2.5)
Language												
BPVS (/150)	**149.0 (1.1)**	**148.9 (1.1)**	**135.1 (24.0)[a]**	**133.9 (21.4)**	118.7 (28.9)[a]	148.0 (1.0)	61.9 (44.1)	98.0 (46.3)	136.3 (30.4)	142.8 (8.4)	120.2 (39.6)	145.2 (4.4)
GNT (/30)	27.3 (2.1)	26.8 (1.8)	**13.7 (8.5)[a]**	**12.4 (6.3)**	11.5 (11.0)[a]	20.0 (4.4)	0.2 (0.4)	0.5 (1.0)	15.8 (6.8)	20.2 (7.6)	6.2 (7.7)	12.4 (7.0)
NART (/50)	45.2 (3.4)	44.6 (3.0)	28.3 (11.9)[a]	37.1 (4.6)	30.7 (13.1)	40.0 (2.0)	16.1 (10.2)	16.3 (13.6)	28.3 (15.0)[f]	30.8 (14.9)	20.4 (18.5)[a]	29.8 (16.9)
Arithmetic												
GDA Total (/24)	**16.6 (6.4)**	**16.1 (4.0)**	**4.9 (5.0)[e]**	**4.8 (5.4)**	8.2 (7.4)[a]	8.3 (7.2)	13.3 (5.5)	7.3 (4.0)	5 (3.6)[d]	6.4 (7.2)	1.7 (1.5)	7 (5.8)
Visuospatial												
VOSP OD (/20)	*19.5 (0.7)*	*18.4 (1.2)*	*16.1 (2.6)[a]*	*13.0 (3.0)*	14.0 (4.2)	17.0 (1.0)	15.3 (6.3)[a]	14.5 (5.9)	16.8 (3.6)[b]	17.6 (2.1)	15.8 (3.1)	15.8 (2.7)
Executive												
DS (Reverse) (/12)	**8.0 (2.6)**	**8.2 (2.2)**	4.6 (1.9)[c]	4.0 (1.9)	5.0 (3.1)[a]	4.0 (0.0)	6.9 (2.7)	5.5 (2.7)	4.4 (1.7)[d]	5.2 (4.2)	2.4 (1.1)[a]	4.8 (1.8)
Letter fluency (60s, "F")	**19.7 (6.0)**	**20.8 (4.2)**	10.3 (5.2)	11.9 (7.4)	6.8 (3.9)[a]	7.3 (2.3)	7.0 (3.6)[b]	10.8 (7.0)	4.8 (5.8)[d]	14.0 (8.2)[a]	*2.6 (3.8)[a]*	*11.3 (3.9)*
Category fluency (60s, "Animals")	**25.0 (6.6)**	**24.7 (5.4)**	**10.0 (5.6)**	**10.4 (6.7)**	8.0 (6.1)	10.3 (6.0)	6.1 (5.3)[a]	9.8 (6.2)	8.6 (5.8)[d]	18.6 (11.5)	5.7 (5.8)	11.6 (8.1)

Mean (standard deviation) values of performance on neuropsychology tests are shown (maximum scores are indicated in parentheses) for each testing environment (face-to-face vs remote research setting) for each patient group. **Bold** indicates values for which there was strong evidence in support of the null hypothesis (H0); *italics* indicate values for which there was strong evidence in support of the alternative hypothesis (H1). Exact values for the Bayes factor comparing H0 against H1 (BF_{01}) are presented in Table S3 (Supplementary file). A reduced number of participants completed certain tests, as follows: [a]n-1, [b]n-2, [c]n-3, [d]n-4, [e]n-5, [f]n-6, [g]n-9, [h]n-16. AD, patient group with typical Alzheimer's disease; BPVS, British Picture Vocabulary Scale [20]; bvFTD, patient group with behavioural variant frontotemporal dementia; CTL, healthy control group; DS, Digit Span [18]; F2F, face-to-face; GDA, Graded Difficulty Arithmetic test [57]; GNT, Graded Naming Test [19]; LPA, patient group with logopenic progressive aphasia; NA, not available; NART, National Adult Reading Test [21]; PNFA, patient group with progressive nonfluent aphasia; RMT, Recognition Memory Test [17]; SD, patient group with semantic dementia; VOSP OD, Visual Object Space Perception Object Decision task [22]; WASI, Wechsler Abbreviated Scale of Intelligence [16].

For peer review only - http://bmjopen.bmj.com/site/about/guidelines.xhtml

Table 3. Neurolinguistic performance for all participant groups: comparison of face-to-face and remote test administration.

Test	CTL		SD		PNFA		LPA	
	F2F	Remote	F2F	Remote	F2F	Remote	F2F	Remote
**No. tested**	10	10	9	4	12	5	6	5
Phoneme perception								
PALPA 3 (/36)	34.9 (1.6)	33.8 (2.0)	35.3 (1.3)	33.5 (2.6)	31.9 (5.7)	34.0 (1.6)	33.8 (2.4)[a]	32.0 (4.0)
Reading								
Non word reading (/25)	24.4 (0.8)	24.9 (0.3)	19.6 (4.9)	16.0 (9.8)	19.3 (5.7)[d]	15.8 (8.7)	14.5 (5.4)	16.4 (9.6)
Regular reading (/25)	25.0 (0.0)	25.0 (0.0)	22.8 (3.9)[d]	21.3 (6.2)	23.3 (2.2)[g]	22.2 (4.2)	24.5 (0.7)[d]	21.8 (6.1)
Irregular reading (/25)	24.7 (0.7)	25.0 (0.0)	18.8 (5.8)[d]	18.3 (8.9)	19.8 (6.4)[g]	21.6 (4.4)	21.5 (3.5)[d]	22.6 (2.1)
Naming								
BNT (/30)	**29.2 (0.8)**	**29.0 (1.0)**	3.3 (3.4)	9.5 (7.1)	24.7 (4.4)[b]	24.4 (7.6)	9.7 (8.0)	16.8 (8.8)
Semantic association								
Camel and cactus (/32)	**31.0 (1.0)**	**30.9 (1.1)**	22.3 (5.1)[e]	25.5 (2.6)	28.3 (5.5)[h]	30.0 (1.4)	29.0 (0.0)[e]	29.0 (2.1)
Word comprehension								
Concrete synonyms (/25)	24.5 (0.5)	24.3 (0.5)	15.5 (2.9)[c]	17.0 (4.0)[a]	22.2 (3.2)[b]	21.0 (4.2)	15.6 (9.4)[a]	21.4 (3.5)
Abstract synonyms (/25)	24.8 (0.4)	24.3 (0.8)	14.4 (1.5)[d]	17.3 (1.2)[a]	21.4 (3.7)[b]	21.2 (3.4)	16.5 (11.2)[b]	22.3 (2.1)[a]
Sentence comprehension								
PALPA 55 (/24)	23.8 (0.4)	23.1 (1.3)	22.6 (1.6)[a]	21.8 (2.2)	19.6 (4.8)[a]	21.0 (2.6)	18.8 (2.6)[a]	20.0 (2.1)
Speech repetition								
Monosyllabic word repetition (/15)	*14.6 (0.5)*	*12.7 (1.6)*	14.8 (0.4)	10.8 (3.2)	12.8 (2.6)[c]	10.0 (4.2)	14.0 (1.2)[b]	11.2 (3.3)
Bisyllabic word repetition (/15)	**14.7 (0.5)**	**14.7 (0.7)**	14.8 (0.7)	13.0 (1.6)	12.8 (3.3)[c]	12.4 (4.7)	12.0 (2.9)[b]	14.8 (0.4)
Trisyllabic word repetition (/15)	15.0 (0.0)	15.0 (0.0)	14.8 (0.4)	12.8 (3.2)	11.1 (5.9)[c]	12.0 (6.2)	12.3 (2.9)[b]	14.0 (1.0)
Graded difficulty sentence repetition (/10)	9.8 (0.4)	9.3 (0.9)	8.3 (1.2)	7.3 (1.7)	4.6 (3.0)[e]	5.2 (3.2)	4.7 (2.9)[c]	5.2 (1.6)
Sentence construction								
Spoken sentences (/25)	25.0 (0.0)	24.9 (0.3)	19.1 (7.5)	23.5 (1.3)	18.0 (7.4)[d]	21.6 (5.1)	18.0 (5.3)[c]	22.8 (2.7)

Mean (standard deviation) values of performance on neurolinguistic tests are shown (maximum scores are indicated in parentheses) for each testing environment (face-to-face vs remote) for each participant group. **Bold** indicates values for which there was strong evidence in support of the null hypothesis (H0); *italics* indicate values for which there was strong evidence in support of the alternative hypothesis (H1). Exact values for the Bayes factor comparing H0 against H1 (BF_{01}) are presented in Table S4 (Supplementary file). A reduced number of participants completed certain tests: [a]n-1, [b]n-2, [c]n-3, [d]n-4, [e]n-5, [f]n-6, [g]n-8, [h]n-9. In the spoken sentence construction test, participants are given five different words (walked, radio, throw, green, tree) and are asked to produce a sentence that contains each word in turn. Sentences are then scored based on grammar and semantic sense, each sentence counting for a maximum of 5 points. BNT, Boston Naming Test [27]; CTRL, healthy control group; F2F, face-to-face; LPA, patient group with logopenic progressive aphasia; N, number of participants per group; PALPA, Psycholinguistic Assessment of Language Processing in Aphasia subtests [24]; PNFA, patient group with progressive nonfluent aphasia; SD, patient group

For peer review only - http://bmjopen.bmj.com/site/about/guidelines.xhtml

with semantic dementia.

For peer review only - http://bmjopen.bmj.com/site/about/guidelines.xhtml

Where applicable, we sought permission from the test publishers to adapt tests for remote administration.

Testing procedure – remote

An initial session was conducted via Zoom to accustom participants to the remote testing format, check the screen and sound sharing options on Zoom, and that the quality of their internet connection was acceptable.

Participants were permitted to use their preferred device (tablet, laptop, or desktop computer). To ensure screen visibility, we did not accept the use of smartphones. Most participants (90%) listened via speakers; six participants used headphones, and device volume was set to a comfortable level by each participant or their caregiver. Remote assessments were scheduled to ensure that testing could be completed in a quiet environment with minimal distractions. Where a task required visual presentation, this was done by screen sharing the Microsoft Powerpoint presentation containing the scanned stimuli for that task in full screen mode. Each patient's primary caregiver was asked to be available during each research session in case of any problems with using the equipment; in practice, no major technological issues arose.

To provide a check on basic audibility in the remote testing environment, before each remote session, participants first listened to a set of 10 sentences from the Bamford-Kowal-Bench (BKB) list[35]. These sentences have previously been validated in hearing-impaired children. The spoken sentences were delivered online using an online experiment builder, Labvanced[36]. In each trial, a spoken sentence was played to the participant via screen and sound share on Zoom and the participant was encouraged to select the last word in the sentence they had just heard from three possible options presented visually via screen share (see Figure S1, Supplementary file). A perfect score on the final three items was required for the participant to proceed to the remote testing session proper (this allowed each participant and/or caregiver to manually adjust the volume to a comfortable level during the first seven sentences). Most participants (95%) performed at ceiling across all ten items, and no participant made an error on any of the final three items, meaning that none was rejected based on their BKB performance (see Table S2, Supplementary file). The order of sentences was fixed across participants.

The remote neuropsychological and neurolinguistic batteries each took around an hour to administer. To minimise fatigue[37], batteries were delivered in separate testing sessions typically within a week (and never more than two weeks apart).

At the end of each testing session, each participant was debriefed by the experimenter. This provided them with the opportunity to raise any technical issues and give their impressions of the remote testing session. No technical difficulties were reported; all participants said they had felt comfortable with remote testing.

Statistical analysis

All statistical analyses were performed in JASP (version 0.16).

The remote and face-to-face patient cohorts were compared on demographic characteristics using independent samples t-tests and Wilcoxon rank-sum tests. Healthy controls' scores in remote and face-to-face testing environments were compared using paired samples t-tests or

For peer review only - http://bmjopen.bmj.com/site/about/guidelines.xhtml

(where the assumption of normality was not met) Wilcoxon signed rank tests. To reduce Type I error, no corrections for multiple comparisons were applied.

We did not perform between group comparisons of neuropsychological and neurolinguistic performance as these syndromic profiles of the neuropsychological and neurolinguistic tests have been reviewed and published previously[38 39].

Our null hypothesis was that there would be no effect of testing environment on neuropsychological performance – i.e., no differences in performance between remote and face-to-face assessment settings – for any participant group. To critically assess the magnitude of evidence in favour of this null hypothesis versus the alternative hypothesis (i.e., that there was in fact an effect of testing environment) particularly in light of the relatively small patient cohorts here, we employed a Bayesian approach[40]. Bayesian independent samples t-tests (and non-parametric equivalents where assumptions of the general linear model were violated) were performed for each general neuropsychological and neurolinguistic test in each patient group separately. As numbers in some groups were quite small, we also conducted analyses for a combined patient cohort in both environments. Healthy control performance was compared using Bayesian paired samples t-tests (or appropriate non-parametric equivalent). A Bayes factor, which is the ratio of evidence supporting the null hypothesis over the alternative hypothesis (hereafter BF_{01}) was calculated for each comparison using JASP. A BF_{01} value > 3 indicates strong evidence in favour of the null hypothesis while a value < 0.33 supports the alternative hypothesis; BF_{01} values between 0.33 and 3 are classified as 'anecdotal' evidence, comparable to non-significant differences in inferential statistics[41 42]. Bayes factor values are presented in Tables S3 and S4 (Supplementary file).

Patient and public involvement

In August and September 2020, we contacted 527 people (comprising healthy control participants and people with a diagnosis of a dementia) who had previously taken part in our face-to-face research programmes in the Dementia Research Centre, UCL, or who had expressed an interest in doing so in the future. They were asked, "Would you consider participating in research remotely (telephone/ online)?" Of the 163 people who answered the question, 145 (89%) indicated that they would be happy to take part in remote research. Based on this feedback, we submitted an amendment to our existing research ethics that was approved in October 2020. Following this, we conducted a pilot remote testing session with an older healthy control individual who was also a carer for a family member living with dementia. Their feedback was instrumental in developing and improving our remote testing procedure.

Results from this work will be disseminated to members of the support groups that we run with Rare Dementia Support (www.raredementiasupport.org) through online presentations at webinars and research summaries in newsletters.

3. Results

General characteristics of participant groups are presented in Table 1; performance on the general neuropychological battery is given in Table 2; performance on the neurolinguistic battery is shown in Table 3. Figure 1 and Figure 2 show radar plots of performance for each participant group for the general neuropsychological and neurolinguistic batteries, respectively.

For peer review only - http://bmjopen.bmj.com/site/about/guidelines.xhtml

Figure 3 and Figure 4 show performance profiles of healthy control participants on the general neuropsychological and neurolinguistics batteries, respectively.

General characteristics of participant groups

There were no significant differences in age, years of education or symptom duration between the face-to-face and remote testing patient cohorts (Table 1).

Below we highlight comparisons where there was strong evidence in support of either the null (i.e. no difference between remote and face-to-face performance) or alternative (i.e. difference between remote and face-to-face performance) hypothesis. Comparisons are shown in full in Tables S3 and S4 (Supplementary file).

General neuropsychological assessment

Overall, there was little evidence for a significant effect of assessment environment on general neuropsychological test performance in any participant group.

Healthy individuals scored equally well on the digit span reverse, the British Picture Vocabulary Scale (BPVS), the Graded Difficulty Arithmetic (GDA), and on both letter and category fluency tests (all $BF_{01}>3$ indicating strong evidence in favour of the null hypothesis). However, they performed less well on the Visual Object and Spatial Perception Object Decision task (VOSP) ($BF_{01}=0.0404$, indicating strong evidence in favour of the alternative hypothesis) in remote testing than in face-to-face testing, though absolute performance differences were relatively small (remote mean=18.4; face-to-face mean=19.5; Tables 2 and S3, Figure 1 and Figure 3).

For the comparisons of the combined remote vs combined face-to-face patient cohorts, there was strong evidence supporting the null hypothesis for all neuropsychological tests (all $BF_{01}>3$), except for the NART and both letter and category fluency tests, where evidence in support of the null hypothesis was anecdotal (Tables 2 and S3).

For individual patient groups (Tables 2 and S3, Figure 1), there was strong evidence to suggest that the remote AD cohort performed similarly to the face-to-face AD cohort on WASI matrix reasoning, digit span forward, graded naming test (GNT), BPVS, GDA, and category fluency test (all $BF_{01}>3$). However, the remote AD cohort performed less well on the VOSP (mean=13.0; $BF_{01}=0.171$, strong evidence) compared to the face-to-face cohort (mean=16.1). Conversely, LPA patients who completed the letter fluency test remotely (mean words=11.3) performed better than those who completed the same task face-to-face (mean=2.6; BF01=0.188, strong evidence).

No other comparisons yielded strong evidence in support of either hypothesis (Table S3, Supplementary file).

Neurolinguistic assessment

Overall, there was little evidence for a significant effect of assessment environment on neurolinguistic test performance in any participant group.

For peer review only - http://bmjopen.bmj.com/site/about/guidelines.xhtml

Healthy individuals scored equally well on the Boston Naming Test (BNT), the camel and cactus test, and the bisyllabic single word repetition test (all BF_{01}>3, indicating strong evidence in favour of the null hypothesis). However, they performed less well on the monosyllabic word repetition test (BF01=0.0487, strong evidence in favour of the alternative hypothesis) in remote testing than in face-to-face testing, though the absolute performance difference was quite small (remote mean=12.7; face-to-face mean=14.6; Tables 3 and S4, Figure 2 and Figure 4).

The comparisons between combined patient cohorts for remote vs face-to-face testing showed strong evidence supporting the null hypothesis for non-word reading, concrete synonyms, the PALPA-55, and bisyllabic and trisyllabic single word repetition tests (all BF_{01} values>3). There was anecdotal evidence supporting the null hypothesis on all other neurolinguistic tests (all BF_{01} values higher between 1 and 3).

Individual patient group comparisons across environments did not yield strong evidence in support of either hypothesis.

4. Discussion

The present findings suggest that administration of neuropsychological tasks remotely over the internet with healthy older adults and people with a diverse range of dementia phenotypes is broadly feasible.

Our Bayesian analytical approach demonstrated that there was anecdotal or strong evidence suggesting comparable performance across testing environments of healthy participants and AD patients on a range of general neuropsychological and neurolinguistic tests, specifically those targeting working memory (digit span forward), executive functioning (digit span reverse, letter and category fluency tests, WASI matrix reasoning), arithmetic skills (GDA), and general semantic knowledge (BNT, BPVS, GNT). These results corroborate previous reports of preserved neuropsychological performance on executive function, working memory, and language tests across testing environments in both healthy individuals and AD patients[10]. Our findings also corroborated previous work suggesting that remote assessments are viable for people with PPA[43].

Healthy controls and AD participants both performed significantly worse on the remote version of the VOSP object decision task, in which participants are presented with four silhouettes and asked to select the drawing of a real object; the three distractor silhouettes are based on nonsense shapes. The typical amnestic AD phenotype can include prominent visuospatial impairments[44 45] and it is feasible that a reduction in stimulus quality may have stressed cortical apperceptive mechanisms still further, akin to a dynamic 'stress test' of degraded input processing[34 46 47]. However, it is worth noting that there was no such discrepancy across the AD cohorts for other tasks involving visual administration (e.g., WASI Matrix). For the healthy controls, the absolute performance difference across environments was relatively small (mean reduction of 1.1 points). It is also possible that this reduction at least in part reflected normal healthy ageing, as the healthy control cohort was tested on the remote battery between three to four years after their face-to-face assessment.

Healthy control participants also performed significantly worse on the monosyllabic single word repetition task when delivered remotely. The videoconferencing software may have

For peer review only - http://bmjopen.bmj.com/site/about/guidelines.xhtml

degraded the fidelity of the raw speech signal[48], essentially resulting in a harder task than when administered face-to-face. This is potentially consistent with the controls' preserved performance on the bisyllabic single word repetition test where top-down information can be used to complement bottom-up auditory information partially degraded by the videoconferencing software[49]. An alternative (or complementary) explanation could again be age-related changes, here affecting hearing function (presbycusis)[50-52].

The finding of significantly better performance on the verbal (letter) fluency task in the LPA cohort tested remotely compared to the cohort tested face-to-face is surprising. The obvious explanation is that the remote cohort was overall less impaired than the patients seen face-to-face; although efforts were made to match the two cohorts for disease severity and other potentially relevant factors. An alternative explanation could be that participants found the remote setting less anxiety-provoking than face-to-face testing in an unfamiliar environment. Patients with LPA may be relatively susceptible to anxiety as a factor modulating cognitive performance[53 54]. Additionally, as word retrieval is an intrinsically dynamic process that is likely to be facilitated by the availability of 'prompts'[55], patients may have benefitted from cueing of word retrieval by their more familiar home environments.

The way the neuropsychology and neurolinguistic testing protocol was adapted for remote delivery may have favoured null differences. The testing sessions were shorter and spread out within a week, which may have helped counteract the effect of anxiety related to the unfamiliarity of the remote testing setting, as well as potential 'Zoom' fatigue[37]. The increased flexibility of scheduling compared to face-to-face testing in addition to the absence of potentially stressors associated with a face-to-face research visit (e.g., travelling, being in a unfamiliar environment) may have led to participants feeling more relaxed when taking part remotely vs face-to-face: future research should explore this. Furthermore, certain tests selected for remote delivery may have been intrinsically less susceptible to changes in testing protocol (e.g., BPVS), whereas we deliberately excluded tests that we considered would not be practical or suboptimal for remote delivery (e.g., WASI Block design, Baxter spelling test, Trails). Anecdotally, participants reported satisfaction with the remote testing protocol.

The current study presents several limitations which should inform future work. First, while most statistical comparisons indicated similar performance between testing environments for healthy and dementia participants, they were not all supported by strong evidence and certain comparisons even led to the opposite conclusion. Second, the present study was not ideally designed to compare the two testing environments, as the patient cohorts were different and the healthy control participants were not tested simultaneously in both environments within the same year. These findings would therefore need to be replicated in larger cohorts with the same patients in each test situation, to rule out the possibility of small differences observed in favour of face-to-face testing – and in particular, to assess the extent of individual variability in any differential effect of test environment.. Patients of equivalent disease severity would also need to be tested to compare the differential impact of diagnosis on remote performance over the course of the illness. Third, here we did not control for potential deficits in peripheral hearing as these are difficult to measure remotely without adequate equipment. Fourth, we manually adapted face-to-face tasks for remote administration, but there are now several established fully integrated online neuropsychological test batteries that have shown success in assessing patients with neurodegenerative disease remotely[56]: future research could explore the extent to which our results are comparable with those obtained by such batteries.

For peer review only - http://bmjopen.bmj.com/site/about/guidelines.xhtml

Overall, the present findings demonstrate that, despite challenges in setting up remote testing protocols (specifically due to technological requirements), these produce similar results to face-to-face testing protocols. These are encouraging findings given the current climate and anticipating that research participants may continue to favour remote (or hybrid) visits over face-to-face assessments for reasons of convenience as well as safety, as we move beyond the COVID-19 pandemic.

For peer review only

Acknowledgments

We are grateful to all participants for their involvement.

Funding statement

The Dementia Research Centre is supported by Alzheimer's Research UK, Brain Research UK, and The Wolfson Foundation. This work was supported by the Alzheimer's Society, the Royal National Institute for Deaf People, Alzheimer's Research UK, the National Institute for Health Research University College London Hospitals Biomedical Research Centre, and the University College London Leonard Wolfson Experimental Neurology Centre (grant PR/ylr/18575). MCRK is supported by a Wellcome Trust PhD Studentship (102129/B/13/Z). JJ is supported by a Frontotemporal Dementia Research Studentship in Memory of David Blechner (funded through The National Brain Appeal). EB was supported by a Brain Research UK PhD Studentship. RLB was supported by an MRC PhD Studentship in Mental Health. SJC was supported by grants from ESRC-NIHR (ES/L001810/1), EPSRC (EP/M006093/1) and Wellcome Trust (200783). CJDH was supported by a Royal National Institute for Deaf People–Dunhill Medical Trust Pauline Ashley Fellowship (grant PA23_Hardy) and a Wellcome Institutional Strategic Support Fund Award (204841/Z/16/Z).

Competing interests

The authors have no competing interests to declare.

Data availability statement

The data that support the findings of this study are available on request from the corresponding author.

Author contributions

MCRK, JJ, LD, EB, LR, RLB, EVB, SB, JDR, SJC, JDW and CJDH contributed to the conception and design of the study. MCRK, JJ, LD, EB, LR, RLB, EVB and CJDH contributed to the acquisition of data. MCRK, JJ, JDW and CJDH were involved in the analysis and interpretation of data, and wrote the first draft of the article. MCRK, JJ, LD, EB, LR, RLB, EVB, SB, JDR, SJC, JDW and CJDH were involved in revision of the draft and approved the final version of the manuscript.

Ethics Approval

Ethical approval was granted by the University College London and National Hospital for Neurology and Neurosurgery Joint Research Ethics Committees in accordance with the Declaration of Helsinki (Refs 06N032 and 150508).

For peer review only - http://bmjopen.bmj.com/site/about/guidelines.xhtml

For peer review only

For peer review only - http://bmjopen.bmj.com/site/about/guidelines.xhtml

References

1. Wang QQ, Davis PB, Gurney ME, et al. COVID-19 and dementia: Analyses of risk, disparity, and outcomes from electronic health records in the US. *Alzheimers Dement* 2021;17(8):1297-306. doi: 10.1002/alz.12296
2. Poon P, Hui E, Dai D, et al. Cognitive intervention for community-dwelling older persons with memory problems: telemedicine versus face-to-face treatment. *Int J Geriat Psychiatry* 2005;20(3):285-6. doi: 10.1002/gps.1282
3. Jelcic N, Agostini M, Meneghello F, et al. Feasibility and efficacy of cognitive telerehabilitation in early Alzheimer's disease: a pilot study. *Clin Interv Aging* 2014;9:1605-11. doi: 10.2147/CIA.S68145
4. Costanzo MC, Arcidiacono C, Rodolico A, et al. Diagnostic and interventional implications of telemedicine in Alzheimer's disease and mild cognitive impairment: a literature review. *Int J Geriatr Psychiatry* 2020;35(1):12-28.
5. Dial HR, Hinshelwood HA, Grasso SM, et al. Investigating the utility of teletherapy in individuals with primary progressive aphasia. *Clin Interv Aging* 2019;14:453-71. doi: 10.2147/CIA.S178878
6. Rogalski EJ, Saxon M, McKenna H, et al. Communication Bridge: A pilot feasibility study of Internet-based speech-language therapy for individuals with progressive aphasia. *Alzheimers Dement* 2016;2(4):213-21. doi: 10.1016/j.trci.2016.08.005
7. Capozzo R, Zoccolella S, Frisullo ME, et al. Telemedicine for Delivery of Care in Frontotemporal Lobar Degeneration During COVID-19 Pandemic: Results from Southern Italy. *J Alz Dis* 2020;76(2):481-9. doi: 10.3233/JAD-200589
8. Cuffaro L, Di Lorenzo F, Bonavita S, et al. Dementia care and COVID-19 pandemic: a necessary digital revolution. *Neurol Sci* 2020;41(8):1977-9. doi: 10.1007/s10072-020-04512-4
9. Waddington CG, Harding E, Brotherhood EV, et al. The Development of Virtual Videoconference-Based Support for People Living with Rare Dementias and Their Carers: Three-Phase Support Group Evaluation Protocol. *JMIR Research Protocols* in press
10. Hunter M, Jenkins N, Dolan C, et al. Reliability of Telephone and Videoconference Methods of Cognitive Assessment in Older Adults with and without Dementia. *J Alz Dis* 2021;81:1625-47. doi: 10.3233/JAD-210088
11. Newkirk LA, Kim JM, Tinklenberg JR, et al. Validation of a 26-point telephone version of the Mini-Mental State Examination. *J Geriatr Psychiatry Neurol* 2004;17(2):81-7. doi: 10.1177/0891988704264534
12. Kennedy RE, Williams CP, Sawyer P, et al. Comparison of In-Person and Telephone Administration of the Mini-Mental State Examination in the University of Alabama at Birmingham Study of Aging. *J Am Geriatr Soc* 2014;62(10):1928-32. doi: 10.1111/jgs.13027
13. Dubois B, Feldman HH, Jacova C, et al. Advancing research diagnostic criteria for Alzheimer's disease: the IWG-2 criteria. *Lancet Neurol* 2014;13:614-29.
14. Rascovsky K, Hodges JR, Knopman D, et al. Sensitivity of revised diagnostic criteria for the behavioural variant of frontotemporal dementia. *Brain* 2011;134:2456-77.
15. Gorno-Tempini ML, Hillis AE, Weintraub S, et al. Classification of primary progressive aphasia and its variants. *Neurology* 2011;76:1006-14.
16. Wechsler D. Wechsler Adult Intelligence Scale-Revised. New York: Psychological Corporation 1981.
17. Warrington EK. Recognition memory test. Windsor: NFER-Nelson 1984.

For peer review only - http://bmjopen.bmj.com/site/about/guidelines.xhtml

18. Wechsler D. Wechsler memory scale: Revised. San Antonio, TX: The Psychological Corporation 1987.
19. McKenna P, Warrington EK. Testing for nominal dysphasia. *J Neurol Neurosurgery, Psychiatry* 1987;43:781-8.
20. Dunn LM, Whetton C. British Picture Vocabulary Scale. Windsor, England: NFER-Nelson 1997.
21. Nelson HE, Wilson J. National adult reading test (NART). Windsor, UK: Nfer-Nelson 1991.
22. Warrington EK, James M. The visual object and space perception battery. Bury St Edmunds, UK: Thames Valley Test Company 1991.
23. Delis DC, Kaplan E, Kramer JH. Delis-Kaplan executive function system (D-KEFS). San Antonio, TX: Psychological Corporation 2001.
24. Kay J, Lesser R, Coltheart M. Psycholinguistic Assessments of Language Processing in Aphasia. 1992
25. Snowling M, Stothard J, McLean JF. Graded nonword reading test. *Thames Valley Test* 1996
26. Coltheart M, Besner D, Jonasson JT, et al. Phonological encoding in the lexical decision task. *Q J Exp* 1979;31
27. Kaplan E, Goodglass H, Weintraub S. Boston Naming Test. 1983
28. Moore K, Convery R, Bocchetta M, et al. A modified Camel and Cactus Test detects presymptomatic semantic impairment in genetic frontotemporal dementia within the GENFI cohort. *Appl Neuropsychol Adult* 2022;29(1):112-9. doi: 10.1080/23279095.2020.1716357
29. Warrington EK, Mckenna P, Orpwood L. Single Word Comprehension: A Concrete and Abstract Word Synonym Test. *Neuropsychol Rehabil* 1998;8(2):143-54.
30. McCarthy R, Warrington EK. A two-route model of speech production: evidence from aphasia. *Brain* 1984;107(2):463-85. doi: 10.1093/brain/107.2.463
31. Strauss E, Sherman EMS, Spreen O. A compendium of neuropsychological tests: Administration, norms, and commentary: Oxford University Press 2006.
32. Rohrer JD, Crutch SJ, Warrington EK, et al. Progranulin-associated primary progressive aphasia: A distinct phenotype? *Neuropsychologia* 2010;48(1):288-97. doi: 10.1016/j.neuropsychologia.2009.09.017
33. Hardy CJ, Agustus JL, Marshall CR, et al. Behavioural and neuroanatomical correlates of auditory speech analysis in primary progressive aphasias. *Alzheimer's Research and Therapy* 2017;9(53) doi: 10.1186/s13195-017-0278-2
34. Hardy CJ, Marshall CR, Bond RL, et al. Retained capacity for perceptual learning of degraded speech in primary progressive aphasia and Alzheimer's disease. *Alzheimers Res Ther* 2018;10(1):70. doi: 10.1186/s13195-018-0399-2
35. Bench J, Kowal A, Bamford J. The BKB (Bamford-Kowal-Bench) sentence lists for partially-hearing children. *Br J Audiol* 1979;13(3):108-12. doi: 10.3109/03005367909078884
36. Finger H, Goeke C, Diekamp D, et al. LabVanced: A Unified JavaScript Framework for Online Studies. *International Conference on Computational Social Science* 2016
37. Bailenson JN. Nonverbal overload: A theoretical argument for the causes of Zoom fatigue. *TMB* 2021;2(1) doi: 10.1037/tmb0000030
38. Sivasathiaseelan H, Marshall CR, Agustus JL, et al. Frontotemporal Dementia: A Clinical Review. *Semin Neurol* 2019;39(2):251-63. doi: 10.1055/s-0039-1683379
39. Marshall CR, Hardy CJD, Volkmer A, et al. Primary progressive aphasia: a clinical approach. *J Neurol* 2018;265(6):1474-90. doi: 10.1007/s00415-018-8762-6 [published Online First: 2018/02/03]

For peer review only - http://bmjopen.bmj.com/site/about/guidelines.xhtml

40. Dienes Z. Using Bayes to get the most out of non-significant results. *Front Psychol* 2014;5:781. doi: 10.3389/fpsyg.2014.00781
41. Quintana DS, Williams DR. Bayesian alternatives for common null-hypothesis significance tests in psychiatry: a non-technical guide using JASP. *BMC Psychiatry* 2018;18:178. doi: 10.1186/s12888-018-1761-4
42. Ashworth M, Palikara O, Burchell E, et al. Online and Face-to-Face Performance on Two Cognitive Tasks in Children With Williams Syndrome. *Front Psychol* 2021;11:594465. doi: 10.3389/fpsyg.2020.594465
43. Rao LA, Roberts AC, Schafer R, et al. The Reliability of Telepractice Administration of the Western Aphasia Battery-Revised in Persons With Primary Progressive Aphasia. *Am J Speech Lang Pathol* 2022:1-15. doi: 10.1044/2021_AJSLP-21-00150
44. Day BL, Ocal D, Peters A, et al. Altered visual and haptic verticality perception in posterior cortical atrophy and Alzheimer's disease. *J Physiol* 2022;600(2):373-91. doi: 10.1113/JP282289
45. Mandal PK, Joshi J, Saharan S. Visuospatial perception: an emerging biomarker for Alzheimer's disease. *J Alz Dis* 2012;31 Suppl 3:S117-35. doi: 10.3233/JAD-2012-120901
46. Zarkali A, Adams RA, Psarras S, et al. Increased weighting on prior knowledge in Lewy body-associated visual hallucinations. *Brain Comms* 2019;1(1):fcz007. doi: 10.1093/braincomms/fcz00777
47. Weil RS, Pappa K, Schade RN, et al. The cats - and - dogs test: a tool to identify visuoperceptual deficits in Parkinson's disease. *Mov Disord* 2017;32(12):1789-90. doi: 10.1002/mds.27176
48. Weerathunge HR, Segina RK, Tracy L, et al. Accuracy of Acoustic Measures of Voice via Telepractice Videoconferencing Platforms. *J Speech Lang Hear Res* 2021;64(7):2586-99. doi: 10.1044/2021_JSLHR-20-00625
49. Jiang J, Benhamou E, Waters S, et al. Processing of Degraded Speech in Brain Disorders. *Brain Sci* 2021;11(3):394. doi: 10.3390/brainsci11030394
50. Goodwin MV, Hogervorst E, Maidment DW. The impact of presentation modality on cognitive test performance for adults with hearing loss. *Alzheimers Dement* 2021;17(S12):e058571. doi: 10.1002/alz.058571
51. Helfer KS, Mamo SK, Clauss M, et al. Listening in 2020: A Survey of Adults' Experiences With Pandemic-Related Disruptions. *Am J Audiol* 2021;30(4):941-55. doi: 10.1044/2021_AJA-21-00021
52. Naylor G, Burke LA, Holman JA. Covid-19 Lockdown Affects Hearing Disability and Handicap in Diverse Ways: A Rapid Online Survey Study. *Ear Hear* 2020;41(6):§442-9. doi: 10.1097/AUD.0000000000000948
53. Rohrer JD, Warren JD. Phenomenology and anatomy of abnormal behaviours in primary progressive aphasia. *J Neurol Sci* 2010;293:35-8. doi: 10.1016/j.jns.2010.03.012
54. Magnin E, Chopard G, Ferreira S, et al. Initial neuropsychological profile of a series of 20 patients with logopenic variant of primary progressive aphasia.
. *J Alzheimers Dis* 2013;36(4):799-808. doi: 10.3233/JAD-122335
55. Birn RM, Kenworthy L, Case L, et al. Neural systems supporting lexical search guided by letter and semantic category cues: a self-paced overt response fMRI study of verbal fluency. *Neuroimage* 2010;49(1):1099-107. doi: 10.1016/j.neuroimage.2009.07.036
56. Alosco ML, Mariani ML, Adler CH, et al. Developing methods to detect and diagnose chronic traumatic encephalopathy during life: rationale, design, and methodology for the DIAGNOSE CTE Research Project. *Alzheimers Res Ther* 2021;13(1):136. doi: 10.1186/s13195-021-00872-x

For peer review only - http://bmjopen.bmj.com/site/about/guidelines.xhtml

57. Jackson M, Warrington EK. Arithmetic skills in patients with unilateral cerebral lesions. *Cortex* 1986;22:611-20.

Figure legends

Figure 1. Radar plots of general neuropsychology battery performance, by participant group and testing environment.

Average percentage correct score (plotted on concentric lines) was calculated for each participant group for each test in the neuropsychology battery, across each testing environment. Scores for the fluency tasks were not included here as responses on these tasks cannot be evaluated as correct/incorrect. AD, patient group with typical Alzheimer's disease; BPVS, British Picture Vocabulary Scale; bvFTD, patient group with behavioural variant frontotemporal dementia; DS_For/Back, Digit Span Forwards/Backwards; GDA, Graded Difficulty Arithmetic test; GNT, Graded Naming Test; LPA, patient group with logopenic progressive aphasia; Matrix, WASI Matrix Reasoning; NART, National Adult Reading Test; PNFA, patient group with progressive nonfluent aphasia; RMT, Recognition Memory Test; SD, patient group with semantic dementia; VOSP, Visual Object Space Perception battery.

Figure 2. Radar plots of performance on neurolinguistic battery performance, by participant group and testing environment.

Average percentage score (plotted on concentric lines) was calculated for each participant group for each test in the neurolinguistic battery, across each testing environment. Abstract, abstract synonyms test; bi rep, bisyllabic single word repetition; BNT, Boston Naming Test; C & C, camel and cactus test; concrete, concrete synonyms test; irregular, irregular word reading test; LPA, patient group with logopenic progressive aphasia; mono rep, monosyllabic single word repetition test; non word, non-word reading test; PALPA, Psycholinguistic Assessment of Language Processing in Aphasia subtests; PNFA, patient group with progressive nonfluent aphasia; regular, regular word reading test; sentence rep, graded difficulty sentence repetition test; SD, patient group with semantic dementia; spoken sentences, spoken sentences test; tri rep, trisyllabic single word repetition test.

Figure 3. Performance profiles of healthy control participants on tasks in general neuropsychological battery.

Line plots showing performance profiles of individual healthy control participants on tasks in the general neuropsychological battery. BPVS, British Picture Vocabulary Scale; GDA, Graded Difficulty Arithmetic test; GNT, Graded Naming Test; Matrix, WASI Matrix Reasoning; NART, National Adult Reading Test; VOSP, Visual Object Space Perception Object Decision task.

Figure 4. Performance profiles of healthy control participants on tasks in the neurolinguistic battery.

Line plots indicating percentage scores for each healthy control on representative tests from the neurolinguistic battery administered face-to-face and remotely. Scores on the trisyllabic single word repetition task were jittered slightly on the x-axis to allow for plotting as participants were uniformly at ceiling in both environments. Bi rep, bisyllabic single word

For peer review only - http://bmjopen.bmj.com/site/about/guidelines.xhtml

repetition; BNT, Boston Naming Test; Concrete, concrete synonyms test; F2F, face-to-face; Mono rep, monosyllabic single word repetition test; Non word, non-word reading test; PALPA, Psycholinguistic Assessment of Language Processing in Aphasia; Tri rep, trisyllabic single word repetition.

For peer review only

For peer review only - http://bmjopen.bmj.com/site/about/guidelines.xhtml

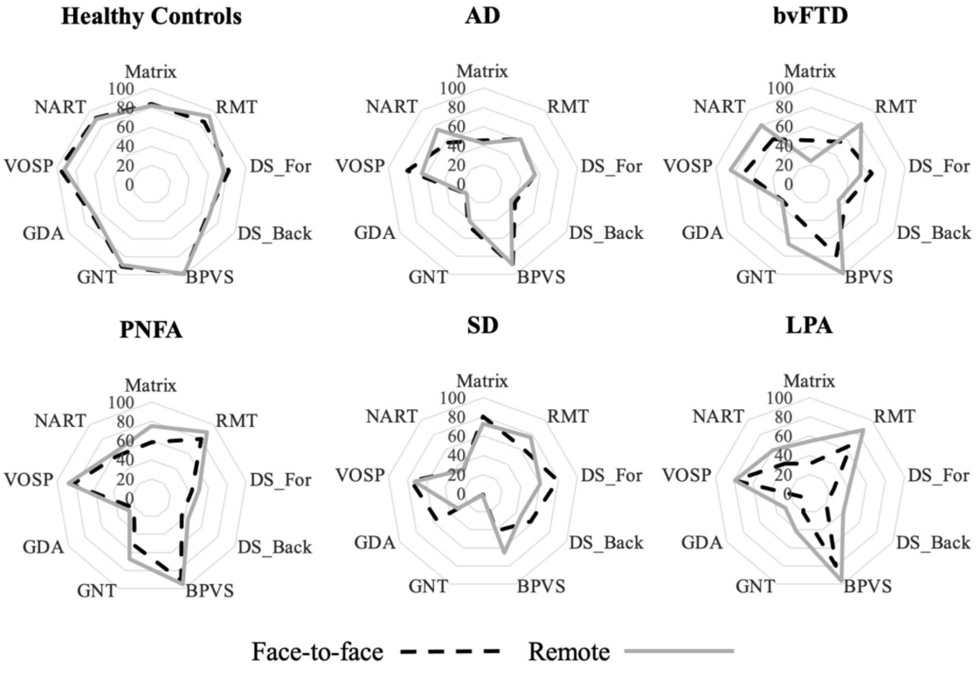

Figure 1. Radar plots of general neuropsychology battery performance, by participant group and testing environment.

Average percentage correct score (plotted on concentric lines) was calculated for each participant group for each test in the neuropsychology battery, across each testing environment. Scores for the fluency tasks were not included here as responses on these tasks cannot be evaluated as correct/incorrect. AD, patient group with typical Alzheimer's disease; BPVS, British Picture Vocabulary Scale; bvFTD, patient group with behavioural variant frontotemporal dementia; DS_For/Back, Digit Span Forwards/Backwards; GDA, Graded Difficulty Arithmetic test; GNT, Graded Naming Test; LPA, patient group with logopenic progressive aphasia; Matrix, WASI Matrix Reasoning; NART, National Adult Reading Test; PNFA, patient group with progressive nonfluent aphasia; RMT, Recognition Memory Test; SD, patient group with semantic dementia; VOSP, Visual Object Space Perception battery.

335x240mm (144 x 144 DPI)

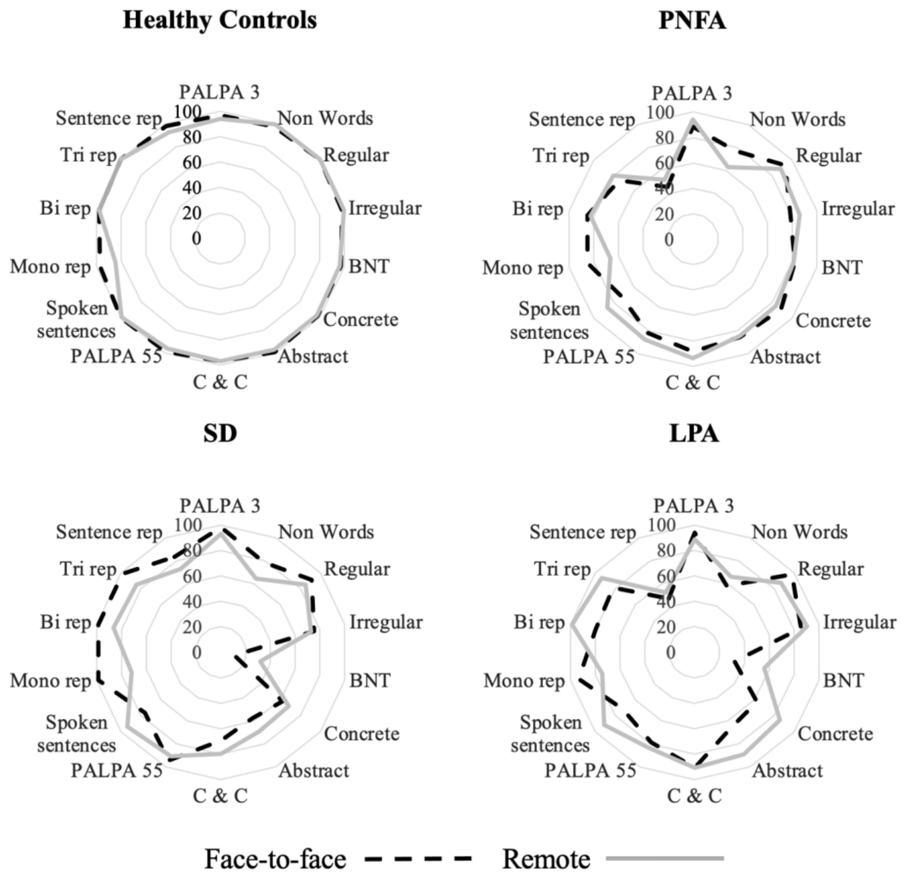

Figure 2. Radar plots of performance on neurolinguistic battery performance, by participant group and testing environment.

Average percentage score (plotted on concentric lines) was calculated for each participant group for each test in the neurolinguistic battery, across each testing environment. Abstract, abstract synonyms test; bi rep, bisyllabic single word repetition; BNT, Boston Naming Test; C & C, camel and cactus test; concrete, concrete synonyms test; irregular, irregular word reading test; LPA, patient group with logopenic progressive aphasia; mono rep, monosyllabic single word repetition test; non word, non-word reading test; PALPA, Psycholinguistic Assessment of Language Processing in Aphasia subtests; PNFA, patient group with progressive nonfluent aphasia; regular, regular word reading test; sentence rep, graded difficulty sentence repetition test; SD, patient group with semantic dementia; spoken sentences, spoken sentences test; tri rep, trisyllabic single word repetition test.

288x262mm (144 x 144 DPI)

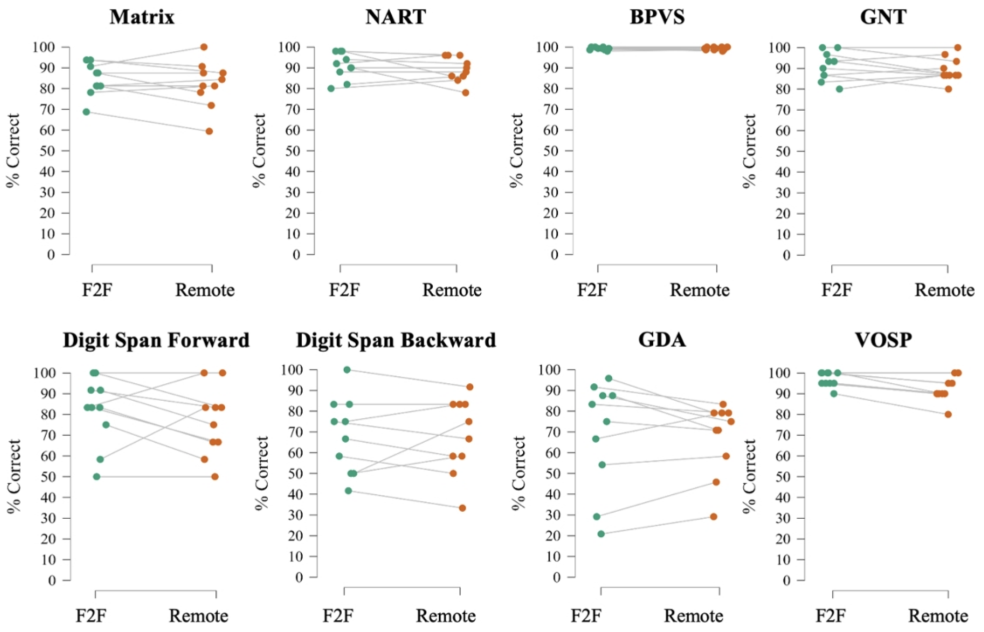

Figure 3. Performance profiles of healthy control participants on tasks in general neuropsychological battery.

Line plots showing performance profiles of individual healthy control participants on tasks in the general neuropsychological battery. BPVS, British Picture Vocabulary Scale; GDA, Graded Difficulty Arithmetic test; GNT, Graded Naming Test; Matrix, WASI Matrix Reasoning; NART, National Adult Reading Test; VOSP, Visual Object Space Perception Object Decision task.

374x247mm (144 x 144 DPI)

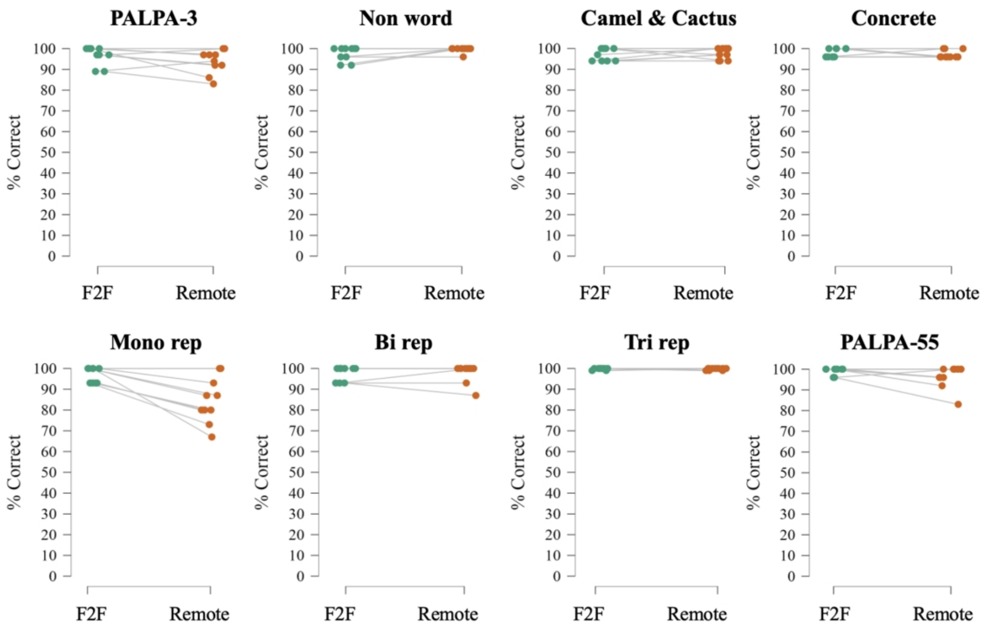

Figure 4. Performance profiles of healthy control participants on tasks in the neurolinguistic battery.

Line plots indicating percentage scores for each healthy control on representative tests from the neurolinguistic battery administered face-to-face and remotely. Scores on the trisyllabic single word repetition task were jittered slightly on the x-axis to allow for plotting as participants were uniformly at ceiling in both environments. Bi rep, bisyllabic single word repetition; BNT, Boston Naming Test; Concrete, concrete synonyms test; F2F, face-to-face; Mono rep, monosyllabic single word repetition test; Non word, non-word reading test; PALPA, Psycholinguistic Assessment of Language Processing in Aphasia; Tri rep, trisyllabic single word repetition.

370x241mm (144 x 144 DPI)

Supplementary Material: Neuropsychological assessments for dementia research in the COVID-19 era: comparing remote and face-to-face testing,

by MC Requena-Komuro, J Jiang et al

Table S1. List of general neuropsychological and neurolinguistic tests delivered face-to-face that were not included in the remote battery.

Test	Reason for removal
WASI Vocabulary	Other tests from same domain already included
WASI Similarities	Other tests from same domain already included
Long Recognition Memory Test for Faces	To reduce the length of the remote battery, this was replaced with the Short Recognition Memory Test for Faces
Long Recognition Memory Test for Words	To reduce length of battery
Camden Paired Associates Learning	Other tests from same domain already included
WASI Block Design	Not feasible online as participants would not have blocks to manipulate
Stroop task	Not feasible online as too difficult for the examiner to determine the participant's target and therefore whether or not they had made an error. Also differences in colour display across participants' screens.
Trails A and B	Not feasible online as requires use pf pencil and paper
WAIS-R Digit Symbol	Not feasible online as requires use of pencil and paper
Usual/Unusual views	Other tests from same domain already included
Modified Kissing and Dancing	Other tests from same domain already included
Baxter Spelling Test	Not feasible online as face-to-face task requires the participant to use pencil and paper; typing responses was not considered appropriate due to potential interference from spell-check software
Written sentences	Not feasible online as face-to-face task requires the participant to use pencil and paper; typing responses was not considered appropriate due to potential interference from spell-check software
Spatial Span Forwards	To reduce length of battery
Spatial Span Backwards	To reduce length of battery

We reduced the number of tests used in our face-to-face general neuropsychological and neurolinguistic batteries down for remote testing, reflecting a) the need to make the remote testing batteries shorter to minimise fatigue; b) impracticalities of administering certain stimuli remotely; and c) inability to adequately record participants' responses to some tasks. The Table shows the tasks that were not included in the remote batteries.

For peer review only - http://bmjopen.bmj.com/site/about/guidelines.xhtml

Table S2. Performance on audibility screening task by remote participant groups

	CTL	AD	bvFTD	SD	PNFA	LPA
Pre neuropsychology battery						
Average number of incorrect items (/10)	0.0 (0.0)	0.1 (0.4)	0.0 (0.0)	0.0 (0.0)	0.2 (0.4)	0.0 (0.0)
Average number of errors on last three items (/3)	0.0 (0.0)	0.0 (0.0)	0.0 (0.0)	0.0 (0.0)	0.0 (0.0)	0.0 (0.0)
Pre neurolinguistic battery						
Average number of incorrect items (/10)	0.0 (0.0)	NA	NA	0.0 (0.0)	0.4 (0.9)	0.0 (0.0)
Average number of errors on last three items (/3)	0.0 (0.0)	0.0 (0.0)	0.0 (0.0)	0.0 (0.0)	0.0 (0.0)	0.0 (0.0)

The data indicate that there were no major background listening environmental confounds nor any significant differences between participant groups (all p>0.05). AD, patient group with typical Alzheimer's disease; bvFTD, patient group with behavioural variant frontotemporal dementia; CTL, healthy control group; LPA, patient group with logopenic progressive aphasia; NA, not applicable; PNFA, patient group with progressive non-Fluent aphasia; SD, patient group with semantic dementia.

For peer review only - http://bmjopen.bmj.com/site/about/guidelines.xhtml

For peer review only

Table S3. Bayesian statistics comparing general neuropsychological test performance on remote vs face-to-face assessments

Test	CTL	All Patients	AD	bvFTD	SD	PNFA	LPA
General intellect							
WASI Matrix	2.56^{t} (Anecdotal)	5.403 (Strong)	3.134^{t} (Strong)	1.477^{t} (Anecdotal)	1.911^{t} (Anecdotal)	1.356^{t} (Anecdotal)	1.441^{t} (Anecdotal)
Episodic memory							
RMT Faces short	NA	3.46 (Strong)	0.411^{t} (Anecdotal)	NA	NA	NA	NA
Working memory							
DS (Forward)	2.663^{t} (Anecdotal)	4.54^{t} (Strong)	3.467^{t} (Strong)	1.948^{t} (Anecdotal)	0.799^{t} (Anecdotal)	2.528^{t} (Anecdotal)	1.598^{t} (Anecdotal)
Language							
BPVS	4.304^{t} (Strong)	4.05 (Strong)	3.363 (Strong)	1.836 (Anecdotal)	1.382^{t} (Anecdotal)	2.754 (Anecdotal)	1.261 (Anecdotal)
GNT	2.923^{t} (Anecdotal)	4.16 (Strong)	3.542 (Strong)	1.407^{t} (Anecdotal)	2.331 (Anecdotal)	1.68^{t} (Anecdotal)	1.325^{t} (Anecdotal)
NART	2.83^{t} (Anecdotal)	1.99 (Anecdotal)	1.552 (Anecdotal)	1.511^{t} (Anecdotal)	2.606^{t} (Anecdotal)	2.52^{t} (Anecdotal)	1.948^{t} (Anecdotal)
Arithmetic							
GDA Total	3.671 (Strong)	5.30 (Strong)	3.33 (Strong)	2.467^{t} (Anecdotal)	0.943^{t} (Anecdotal)	2.483^{t} (Anecdotal)	1.123^{t} (Anecdotal)
Visuospatial							
VOSP	0.0404 (Strong)	4.76 (Strong)	0.171^{t} (Strong)	2.215 (Anecdotal)	2.478 (Anecdotal)	2.553 (Anecdotal)	2.592^{t} (Anecdotal)
Executive							
DS (Reverse)	4.304^{t} (Strong)	5.28 (Strong)	2.746^{t} (Anecdotal)	Variance at 0	1.991^{t} (Anecdotal)	2.659 (Anecdotal)	0.417^{t} (Anecdotal)
Letter fluency	3.159^{t} (Strong)	0.59 (Anecdotal)	2.86^{t} (Anecdotal)	2.426^{t} (Anecdotal)	1.519^{t} (Anecdotal)	0.511^{t} (Anecdotal)	0.188^{t} (Strong)
Category fluency	3.767^{t} (Strong)	1.08 (Anecdotal)	3.532 (Strong)	2.186^{t} (Anecdotal)	1.715^{t} (Anecdotal)	1.027 (Anecdotal)	1.29^{t} (Anecdotal)

For peer review only - http://bmjopen.bmj.com/site/about/guidelines.xhtml

A Bayes factor (BF_{01}) indicates the extent to which the null hypothesis is favoured against the alternative hypothesis (e.g., a BF_{01} value of 4 means that the obtained data are 4 times more likely under the null hypothesis than under the alternative hypothesis). A $BF_{01} > 3$ is therefore considered as strong evidence in support of the null hypothesis; while a BF_{01} of <1/3 is considered as strong evidence in support of the alternative hypothesis. Any values in between are categorised as ‘anecdotal’ evidence, equivalent to a non-significant result in inferential statistics [1]. Results are influenced by the prior (more specifically the shape of the prior influences the strength of the evidence), which can be specified be default using a Cauchy distribution, as here; the Cauchy scale set here is 1.00. The superscript ^{t} indicates that a parametric Bayesian test was used; else the non-parametric Mann Whitney (with 1000 iterative samples) was employed. Blue shading indicates that the alternative hypothesis (H1, i.e. there was a difference in performance across the two environments) was favoured with strong evidence; Green shading indicates that the null hypothesis (H0; i.e. there was no difference in performance across environments) was favoured with strong evidence. AD, patient group with typical Alzheimer’s disease; BPVS, British Picture Vocabulary Scale; bvFTD, patient group with behavioural variant frontotemporal dementia; CTL, healthy control group; DS, Digit Span; F2F, face-to-face; GDA, Graded Difficulty Arithmetic test; GNT, Graded Naming Test; LPA, patient group with logopenic progressive aphasia; Matrix, WASI Matrix Reasoning; NART, National Adult Reading Test; PNFA, patient group with progressive nonfluent aphasia; RMT, Recognition Memory Test; SD, patient group with semantic dementia; VOSP, Visual Object Space Perception battery.

For peer review only - http://bmjopen.bmj.com/site/about/guidelines.xhtml

Table S4. Bayesian statistics comparing neurolinguistic test performance on remote vs face-to-face assessments

Test	CTL	All Patients	SD	PNFA	LPA
Phoneme perception					
PALPA 3	1.003 (Anecdotal)	2.918 (Anecdotal)	1.594 (Anecdotal)	2.731 (Anecdotal)	2.079 (Anecdotal)
Reading					
Non word reading	1.544 (Anecdotal)	3.239 (Strong)	1.940^{t} (Anecdotal)	2.033^{t} (Anecdotal)	2.428^{t} (Anecdotal)
Regular reading	Variance at 0	2.989 (Anecdotal)	1.975 (Anecdotal)	2.305 (Anecdotal)	1.923 (Anecdotal)
Irregular reading	Variance at 0	2.996 (Anecdotal)	2.256 (Anecdotal)	2.194^{t} (Anecdotal)	2.005 (Anecdotal)
Naming					
BNT	3.612^{t} (Strong)	1.995 (Anecdotal)	0.584^{t} (Anecdotal)	2.580 (Anecdotal)	1.490 (Anecdotal)
Semantic association					
Camel and cactus	4.039^{t} (Strong)	1.067^{t} (Anecdotal)	1.524^{t} (Anecdotal)	2.102 (Anecdotal)	N<2 for F2F
Word comprehension					
Concrete synonyms	2.739^{t} (Anecdotal)	3.622 (Strong)	2.009^{t} (Anecdotal)	2.400^{t} (Anecdotal)	1.413^{t} (Anecdotal)
Abstract synonyms	1.726^{t} (Anecdotal)	2.718 (Anecdotal)	0.782 (Anecdotal)	2.741 (Anecdotal)	1. 941 (Anecdotal)
Sentence comprehension					
PALPA55	0.944 (Anecdotal)	3.954 (Strong)	2.246 (Anecdotal)	2.603 (Anecdotal)	2.110 (Anecdotal)
Speech repetition					
Monosyllabic word repetition	0.0487^{t} (Strong)	0.117 (Anecdotal)	0.477^{t} (Anecdotal)	1.167^{t} (Anecdotal)	1.055^{t} (Anecdotal)
Bisyllabic word repetition	4.304^{t} (Strong)	3.744 (Strong)	1.086 (Anecdotal)	2.639 (Anecdotal)	0.650^{t} (Anecdotal)
Trisyllabic word repetition	Variance at 0	3.701 (Strong)	1.339 (Anecdotal)	2.400 (Anecdotal)	1.677 (Anecdotal)
Graded difficulty sentence repetition	1.779 (Anecdotal)	2.983 (Anecdotal)	1.420^{t} (Anecdotal)	2.535^{t} (Anecdotal)	1.996 (Anecdotal)
Sentence construction					
Spoken	Variance at 0	1.525 (Anecdotal)	2.270 (Anecdotal)	1.960. (Anecdotal)	0.930^{t} (Anecdotal)

A Bayes factor (BF_{01}) is shown for each remote vs face-to-face testing comparison. The interpretation and colour coding are as indicated in the legend to Table S3 above. BNT, Boston Naming Test; CTRL, healthy control group; F2F, face-to-face; LPA, patient group with logopenic progressive aphasia; N, number of participants per group; PALPA, Psycholinguistic Assessment of Language Processing in Aphasia subtests; PNFA, patient group with progressive nonfluent aphasia; SD, patient group with semantic dementia

Figure S1. Example of basic audibility screening measure

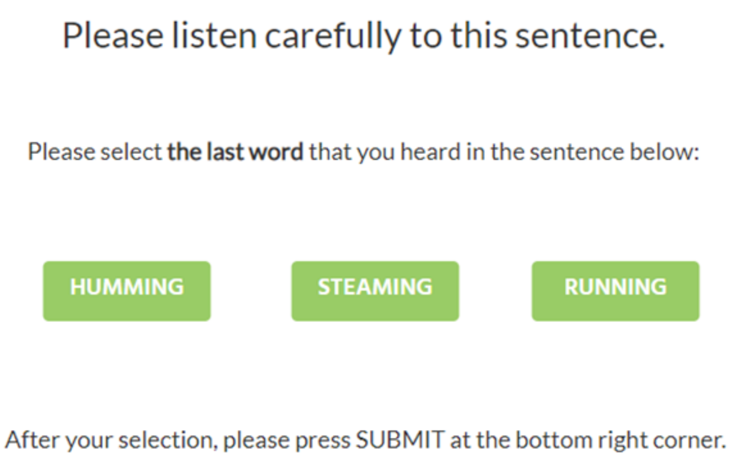

The Figure shows a Labvanced display of the BKB hearing screening measure. In this example, the sentence spoken was “The car engine is running”. For each sentence, two foils were displayed alonside the target, both of which made sense in the sentence when replacing the target. One of the foils was also selected to loosely rhyme with the target word (here, “humming”).

For peer review only - http://bmjopen.bmj.com/site/about/guidelines.xhtml

References

1. Ashworth M, Palikara O, Burchell E, et al. Online and Face-to-Face Performance on Two Cognitive Tasks in Children With Williams Syndrome. *Front Psychol* 2021;11:594465. doi: 10.3389/fpsyg.2020.594465

For peer review only

For peer review only - http://bmjopen.bmj.com/site/about/guidelines.xhtml

STROBE 2007 (v4) Statement—Checklist of items that should be included in reports of *cohort studies*

Section/Topic	Item #	Recommendation	Reported on page #
**Title and abstract**	1	(*a*) Indicate the study's design with a commonly used term in the title or the abstract	2
		(*b*) Provide in the abstract an informative and balanced summary of what was done and what was found	2
**Introduction**			
Background/rationale	2	Explain the scientific background and rationale for the investigation being reported	3
Objectives	3	State specific objectives, including any prespecified hypotheses	4
**Methods**			
Study design	4	Present key elements of study design early in the paper	4-6
Setting	5	Describe the setting, locations, and relevant dates, including periods of recruitment, exposure, follow-up, and data collection	4-6
Participants	6	(*a*) Give the eligibility criteria, and the sources and methods of selection of participants. Describe methods of follow-up	4-6
		(*b*) For matched studies, give matching criteria and number of exposed and unexposed	4
Variables	7	Clearly define all outcomes, exposures, predictors, potential confounders, and effect modifiers. Give diagnostic criteria, if applicable	4-6
Data sources/ measurement	8*	For each variable of interest, give sources of data and details of methods of assessment (measurement). Describe comparability of assessment methods if there is more than one group	*5-6*
Bias	9	Describe any efforts to address potential sources of bias	4
Study size	10	Explain how the study size was arrived at	4
Quantitative variables	11	Explain how quantitative variables were handled in the analyses. If applicable, describe which groupings were chosen and why	6-7
Statistical methods	12	(*a*) Describe all statistical methods, including those used to control for confounding	6-7
		(*b*) Describe any methods used to examine subgroups and interactions	6-7
		(*c*) Explain how missing data were addressed	6-7
		(*d*) If applicable, explain how loss to follow-up was addressed	6-7
		(*e*) Describe any sensitivity analyses	NA
**Results**			

For peer review only

Participants	13*	(a) Report numbers of individuals at each stage of study—eg numbers potentially eligible, examined for eligibility, confirmed eligible, included in the study, completing follow-up, and analysed	4
		(b) Give reasons for non-participation at each stage	4
		(c) Consider use of a flow diagram	NA
Descriptive data	14*	(a) Give characteristics of study participants (eg demographic, clinical, social) and information on exposures and potential confounders	4, 8
		(b) Indicate number of participants with missing data for each variable of interest	16
		(c) Summarise follow-up time (eg, average and total amount)	NA
Outcome data	15*	Report numbers of outcome events or summary measures over time	7-9
Main results	16	(*a*) Give unadjusted estimates and, if applicable, confounder-adjusted estimates and their precision (eg, 95% confidence interval). Make clear which confounders were adjusted for and why they were included	6-9
		(*b*) Report category boundaries when continuous variables were categorized	NA
		(*c*) If relevant, consider translating estimates of relative risk into absolute risk for a meaningful time period	NA
Other analyses	17	Report other analyses done—eg analyses of subgroups and interactions, and sensitivity analyses	7-9
**Discussion**			
Key results	18	Summarise key results with reference to study objectives	9
**Limitations**			
Interpretation	20	Give a cautious overall interpretation of results considering objectives, limitations, multiplicity of analyses, results from similar studies, and other relevant evidence	10
Generalisability	21	Discuss the generalisability (external validity) of the study results	10
**Other information**			
Funding	22	Give the source of funding and the role of the funders for the present study and, if applicable, for the original study on which the present article is based	11

*Give information separately for cases and controls in case-control studies and, if applicable, for exposed and unexposed groups in cohort and cross-sectional studies.

Note: An Explanation and Elaboration article discusses each checklist item and gives methodological background and published examples of transparent reporting. The STROBE checklist is best used in conjunction with this article (freely available on the Web sites of PLoS Medicine at http://www.plosmedicine.org/, Annals of Internal Medicine at http://www.annals.org/, and Epidemiology at http://www.epidem.com/). Information on the STROBE Initiative is available at www.strobe-statement.org.

For peer review only

BMJ Open

Remote versus face-to-face neuropsychological testing for dementia research: a comparative study in patients with Alzheimer’s disease, patients with frontotemporal dementia, and healthy older individuals

Journal:	*BMJ Open*
Manuscript ID	bmjopen-2022-064576.R1
Article Type:	Original research
Date Submitted by the Author:	06-Oct-2022
Complete List of Authors:	Requena-Komuro, Mai-Carmen; University College London Dementia Research Centre; UT Southwestern Medical Center, Kidney Cancer Program<br>Jiang, Jessica; University College London<br>Dobson, Lucianne; University College London<br>Benhamou, Elia; University College London; Cambridge University<br>Russell, Lucy; University College London<br>Bond, Rebecca L; University College London<br>Brotherhood, Emilie V.; University College London<br>Greaves, Caroline; University College London<br>Barker, Suzie; University College London<br>Rohrer, Jonathan; UCL, Queen Square Institute of Neurology<br>Crutch, Sebastian; University College London Institute of Neurology<br>Warren, Jason; UCL<br>Hardy, Chris ; University College London, Dementia Research Centre
**Primary Subject Heading**:	Neurology
Secondary Subject Heading:	Research methods
Keywords:	Dementia < NEUROLOGY, Telemedicine < BIOTECHNOLOGY & BIOINFORMATICS, Adult neurology < NEUROLOGY

SCHOLARONE™
Manuscripts

I, the Submitting Author has the right to grant and does grant on behalf of all authors of the Work (as defined in the below author licence), an exclusive licence and/or a non-exclusive licence for contributions from authors who are: i) UK Crown employees; ii) where BMJ has agreed a CC-BY licence shall apply, and/or iii) in accordance with the terms applicable for US Federal Government officers or employees acting as part of their official duties; on a worldwide, perpetual, irrevocable, royalty-free basis to BMJ Publishing Group Ltd ("BMJ") its licensees and where the relevant Journal is co-owned by BMJ to the co-owners of the Journal, to publish the Work in this journal and any other BMJ products and to exploit all rights, as set out in our licence.

The Submitting Author accepts and understands that any supply made under these terms is made by BMJ to the Submitting Author unless you are acting as an employee on behalf of your employer or a postgraduate student of an affiliated institution which is paying any applicable article publishing charge ("APC") for Open Access articles. Where the Submitting Author wishes to make the Work available on an Open Access basis (and intends to pay the relevant APC), the terms of reuse of such Open Access shall be governed by a Creative Commons licence – details of these licences and which Creative Commons licence will apply to this Work are set out in our licence referred to above.

Other than as permitted in any relevant BMJ Author's Self Archiving Policies, I confirm this Work has not been accepted for publication elsewhere, is not being considered for publication elsewhere and does not duplicate material already published. I confirm all authors consent to publication of this Work and authorise the granting of this licence.

For peer review only

Remote versus face-to-face neuropsychological testing for dementia research: a comparative study in patients with Alzheimer's disease, patients with frontotemporal dementia, and healthy older individuals

Maï-Carmen Requena-Komuro[1,2*], Jessica Jiang[1*], Lucianne Dobson[1], Elia Benhamou[1,3], Lucy Russell[1], Rebecca L Bond[1], Emilie V Brotherhood[1], Caroline Greaves[1], Suzie Barker[1], Jonathan D Rohrer[1], Sebastian J Crutch[1], Jason D Warren[1], Chris JD Hardy[1]

*Joint lead authors

[1]Dementia Research Centre, Queen Square Institute of Neurology, University College London

[2]Kidney Cancer Program, UT Southwestern Medical Center

[3]MRC Cognition and Brain Sciences Unit, University of Cambridge

Correspondence to:
Dr Chris J D Hardy,
Dementia Research Centre
UCL Queen Square Institute of Neurology
1st Floor, 8-11 Queen Square
London WC1N 3AR
United Kingdom
Email: chris.hardy@ucl.ac.uk
Tel: 44 203 448 3676

Keywords: dementia, primary progressive aphasia, frontotemporal dementia, Alzheimer's disease, neuropsychology, remote, online,

For peer review only - http://bmjopen.bmj.com/site/about/guidelines.xhtml

Word count: Abstract: 300 words, Main text: 4323 words

For peer review only

Abstract

Objectives: We explored whether adapting neuropsychological tests for online administration during the COVID-19 pandemic was feasible for dementia research.
Design: We used a longitudinal design for healthy controls, who completed face-to-face assessments three-to-four years before remote assessments. For patients, we used a cross-sectional design, contrasting a prospective remote cohort with a retrospective face-to-face cohort matched for age/education/severity.
Setting: Remote assessments were conducted using video-conferencing/online testing platforms, with participants using a personal computer/tablet at home. Face-to-face assessments were conducted in testing rooms at our research centre.
Participants: The remote cohort comprised 25 patients (n=8 Alzheimer's disease (AD); n=3 behavioural variant frontotemporal dementia (bvFTD); n=4 semantic dementia (SD); n=5 progressive nonfluent aphasia (PNFA); n=5 logopenic aphasia (LPA)). The face-to-face patient cohort comprised 64 patients (n=25 AD; n=12 bvFTD; n=9 SD; n=12 PNFA; n=6 LPA). Ten controls who previously participated in face-to-face research also took part remotely.
Outcome measures: The outcome measures comprised the strength of evidence under a Bayesian framework for differences in performances between testing environments on general neuropsychological and neurolingustic measures.
Results: There was substantial evidence suggesting no difference across environments in both the healthy control and combined patient cohorts (on measures of working memory, single-word comprehension, arithmetic, and naming; Bayes Factors $(BF)_{01}>3$), in the healthy control group alone (on measures of letter/category fluency, semantic knowledge and bisyllabic word repetition; all $BF_{01}>3$), and in the combined patient cohort alone (on measures of executive functioning, episodic memory, short-term verbal memory, visual perception, nonword reading, sentence comprehension, and bisyllabic/trisyllabic word repetition; all $BF_{01}>3$). In the control cohort alone, there was substantial evidence in support of a difference across environments for tests of visual perception (BF_{01}=0.0404) and monosyllabic word repetition (BF_{01}=0.0487).
Conclusions: Our findings suggest that remote delivery of neuropsychological tests for dementia research is feasible.

Strengths and limitations of this study

- Diverse patient cohorts representing rare dementias with specific communication difficulties.
- Sampling of diverse and relevant neuropsychological domains.
- Use of Bayesian statistics to quantify the strength of evidence for the putative null hypothesis (no effect between remote and face-to-face testing).
- Relatively small cohort sizes.
- Lack of direct head-to-head comparisons of test environment in the same patients.

For peer review only - http://bmjopen.bmj.com/site/about/guidelines.xhtml

Introduction

The COVID-19 pandemic and associated social distancing and lockdown measures imposed a series of daunting challenges for conducting research with people with dementia. In the UK, three national lockdowns between March 2020 and February 2021 largely prevented face-to-face research. People with dementia are at increased risk for COVID-19[1], and many participants understandably did not feel safe to travel for research, particularly before widespread vaccination was implemented. Here, we describe our attempts to translate our traditional neuropsychology and neurolinguistics batteries (typically administered face-to-face) for remote administration.

Development and implementation of online cognitive assessments for dementia patients, particularly within communities who experience difficulties in accessing clinical care is not new[2]. Telemedicine has been previously used successfully in Alzheimer's disease (AD)[3 4] and with rarer dementias such as primary progressive aphasia (PPA)[5-7] and behavioural variant frontotemporal dementia (bvFTD)[7]. However, due to COVID-19 there has been a more pervasive shift towards the use of online methods to meet clinical, support and research needs[8 9].

A review by Hunter and colleagues[10] summarises 20 years of research comparing face-to-face and online administration of cognitive tests in healthy older adults (≥40 years old) and participants diagnosed with mild cognitive impairment (MCI), AD, or other types of dementia (often unspecified). The authors identified 12 studies that used videoconferencing methods. Overall, there was clear evidence to suggest that remote cognitive testing for people living with AD and other forms of dementia is feasible. Additionally, there is evidence to suggest that online performance remains stable over time (with a maximum delay of three months between assessments), particularly for the domains of executive function, working memory, verbal episodic memory, and language. Minimal evidence was available for visuospatial tasks, and tests of single word and sentence comprehension.

Notwithstanding considerable progress in this area to date, further research into the feasibility of remote neuropsychological testing of patients with neurodegenerative diseases is required. There are three main considerations that need addressing: (1) Adaptation: are similar performance outcomes obtained on neuropsychological tests designed for face-to-face administration when given remotely? If performance across modalities is equivalent then this could allow for pooling of data collected face-to-face and remotely, potentially allowing more equitable access to research for participants who are not able to physically travel to research centres. (2) Demand: to what extent are research participants with and without dementia willing to engage in remote neuropsychological research? And (3) Acceptability: how satisfactory is remote testing for research participants including those with diverse forms of dementia?[11].

Based largely on the face-to-face protocol for general neuropsychological and neurolinguistic testing used at our research centre, we built a protocol for remote testing of patients diagnosed with typical AD, patients representing major variants of PPA (semantic dementia (SD), progressive nonfluent aphasia (PNFA), logopenic aphasia (LPA)), and bvFTD. Patients were tested from their homes via the widely used video conferencing software, Zoom (Zoom Video Communications Inc). We also recruited a small cohort of healthy older adults who had taken part in our face-to-face research at the Dementia Research Centre three-to-four years before the pandemic. Here, we compared the healthy controls' performance on several

For peer review only - http://bmjopen.bmj.com/site/about/guidelines.xhtml

neuropsychological and neurolinguistic tests between the two testing environments (face-to-face vs remote). We also compared the performance of patients tested remotely with a historical face-to-face cohort of patients chosen to represent the same syndromes and to match the remote cohort based on age, education, and symptom duration. We adopted a Bayesian approach that assesses the amount of evidence in favour of the null hypothesis (i.e., that there is no significant difference in performance on a given neuropsychological task between testing environments) relative to the alternative hypothesis (i.e., that there is a significant difference in performance on a given neuropsychological task between testing environments).

Following previous research[10], we did not predict major differences in terms of participants' performances when tested face-to-face and remotely on most neuropsychological and neurolinguistics tests. However, we did consider the potential for poorer performance on tests of speech perception that were administered remotely, given additional difficulties associated with controlling the remote auditory environment.

Methods

Participant recruitment and group matching

Recruitment for the study took place between February-August 2021. Potential patient participants were identified via the Specialist Cognitive Disorders Clinic at the National Hospital for Neurology and Neurosurgery, direct research referrals from external clinicians or via Rare Dementia Support (www.raredementiasupport.org); healthy controls were recruited via our research participant database. Eighty-seven potentially eligible participants were identified, and all of these were contacted.

An initial telephone screen was conducted for each participant to establish they had access to the necessary equipment (tablet or desktop/laptop computer), a broadband internet connection, a quiet testing space to support the remote research assessment, and no preclusive hearing or visual impairments. We also performed the telephone version of the Mini-Mental State Examination (MMSE) with patients to assess their disease severity[12 13]. A minimum score of 12 on the T-MMSE (which corresponds to a converted MMSE score of 16) was used as an inclusion criterion[12]. No participants were excluded after the telephone screen.

Twenty-five patients (eight with typical AD, three bvFTD, four SD, five PNFA, five LPA) were recruited for the remote study. For comparison purposes, a reference historical cohort comprising 64 patients (25 with AD, 12 bvFTD, nine SD, 12 PNFA, six LPA) who had undertaken a face-to-face research assessment at our Centre between 2013 and 2020 were selected, matching the cohort assessed remotely as closely as possible for syndromic composition, age, years of education and symptom duration. Henceforth, these are referred to as the 'remote' and 'face-to-face' patient cohorts, respectively. All patients fulfilled consensus diagnostic criteria for the relevant syndromic diagnosis[14-16] and all had clinically mild-to-moderate severity disease. Where available, brain MRI was consistent with the syndromic diagnosis, without evidence of significant cerebrovascular burden. Ten healthy older individuals with no history of neurological or psychiatric illness and who had been seen for face-to-face testing three-to-four years previously also underwent remote assessments. The neuropsychological tests reported in this research article were not used for diagnostic purposes. Demographic and clinical details for all participants are summarised in Table 1. All participants gave informed consent for their involvement in the study.

For peer review only - http://bmjopen.bmj.com/site/about/guidelines.xhtml

Table 1. General demographic, clinical and environmental characteristics for all participant groups: comparison of remote and face-to-face cohort characteristics

Characteristic	CTL	AD		bvFTD		SD		PNFA		LPA	
	Remote	F2F	Remote	F2F	Remote	F2F	Remote	F2F	Remote	F2F	Remote
N	10	25	8	12	3	9	4	12	5	6	5
Gender (M/F)	7/3	15/10	5/3	8/4	3/0	7/2	2/2	6/6	2/3	4/2	4/1
Age (yrs)	74.0 (4.1)*	69.5 (4.1)	69.8 (5.9)	70.3 (2.9)	72.0 (5.6)	60.3 (5.2)	58.3 (9.3)	67.1 (5.2)	68.2 (6.4)	69.5 (3.6)	71.6 (4.7)
Education (yrs)	17.6 (0.7)	14.8 (2.5)	16.0 (3.7)	14.9 (2.7)	14.7 (3.1)	16.0 (1.9)	15.5 (3.1)	15.3 (2.3)	14.8 (2.8)	16.0 (1.7)	16.0 (3.3)
Handedness (R/L/A)	9/1/0	23/1/1	8/0/0	11/1/0	3/0/0	9/0/0	3/1/0	10/2/0	5/0/0	5/1/0	5/0/0
Symptom duration (yrs)	NA	6.8 (2.3)	7.3 (3.7)	4.7 (2.1)	3.7 (3.8)	4.1 (1.9)	3.5 (1.9)	3.4 (1.7)	3.2 (0.8)	4.2 (1.9)	3.8 (1.9)
Desktop/Laptop/Tablet	4/5/1	NA	3/2/3	NA	1/2/0	NA	1/3/0	NA	1/2/2	NA	1/3/1
Study Partner Present (Y/N)	NA	NA	5/3	NA	2/1	NA	2/2	NA	2/3	NA	3/2
Interruptions (N)	1	NA	1	NA	1	NA	0	NA	0	NA	0

Mean (standard deviation) values are shown for each group. No significant differences between remote and face-to-face cohorts were found. *on average healthy controls were 3.5 years younger when tested face-to-face; A, ambidextrous; AD, patient group with typical Alzheimer's disease; bvFTD, patient group with behavioural variant frontotemporal dementia; CTL, healthy control group; F, female; F2F, face-to-face; L, left; LPA, patient group with logopenic progressive aphasia; M, male; N, number of participants per group; NA, not applicable; PNFA, patient group with progressive non-fluent aphasia; R, right; SD, patient group with semantic dementia.

For peer review only - http://bmjopen.bmj.com/site/about/guidelines.xhtml

Testing procedure – face-to-face

Data for the reference historical cohort were collected under our face-to-face research assessment protocol, as delivered in experimental sessions at the Dementia Research Centre between 2013 and 2020. Under this protocol, all neuropsychological tests were administered in dedicated quiet testing rooms, with the participant sitting opposite the experimenter. Patients were predominantly tested on their own, unless the informant accompanying them to the study visit requested to be present and the participant agreed to this. In these cases, the informant was explicitly asked not to intervene during testing. No feedback was given on performance and no time limits were imposed (unless timing was intrinsic to the test). General neuropsychological and neurolinguistic test batteries (see Tables 2 and 3) were administered, following standard methods. The neurolinguistic battery was developed specifically to characterise the language profiles of people with PPA and therefore, was not administered to bvFTD or AD participants.

Modifying the face-to-face neuropsychological battery for remote delivery

We reviewed the general neuropsychological and neurolinguistic batteries that had been used historically at our Centre for face-to-face administration, in order to identify tests that could be feasibly delivered remotely online while preserving the overall structure of the batteries and sampling across cognitive domains as far as possible (see Table S1, Supplementary file). Where a task required visual stimulus presentation, a high-quality copy of the stimuli was made. Images were then imported into Microsoft Powerpoint for subsequent presentation to the participant via screen share.

Tests that were retained for remote testing (i.e., tests administered to both the remote and face-to-face patient cohorts) are itemised in Tables 2 and 3. Where applicable, we sought permission from the test publishers to adapt tests for remote administration.

Testing procedure – remote

An initial session was conducted via Zoom to accustom participants to the remote testing format, check the screen and sound sharing options on Zoom, and that the quality of their internet connection was acceptable. Technical aspects of the set-up for remote testing are detailed in additional text and Figure S1 in Supplementary Material online.

The remote neuropsychological and neurolinguistic batteries each took around an hour to administer. To minimise fatigue[17], batteries were delivered in separate testing sessions typically within a week (and never more than two weeks apart).

Feedback on remote testing experience

At the end of each remote testing session, the experimenter debriefed each participant. This provided them with the opportunity to raise any technical issues, give their impressions of the remote testing session and note any distractions that may have occurred for them.

For peer review only - http://bmjopen.bmj.com/site/about/guidelines.xhtml

Where time allowed, at the end of the session, participants were also asked by the experimenter to indicate on a 10-point integer scale how comfortable they had felt with the remote testing format, with 10 indicating ‘very comfortable’.

Statistical analysis

All statistical analyses were performed in JASP (version 0.16).

The remote and face-to-face patient cohorts were compared on demographic characteristics using independent samples t-tests and Wilcoxon rank-sum tests. Healthy controls’ scores in remote and face-to-face testing environments were compared using paired samples t-tests or (where the assumption of normality was not met) Wilcoxon signed rank tests. Healthy controls’ and patients’ ratings of comfort after the general neuropsychology and neurolinguistic sessions with the remote testing set-up were compared using Wilcoxon rank sum tests. To reduce Type I error, no corrections for multiple comparisons were applied.

We did not perform between group comparisons of neuropsychological and neurolinguistic performance as these syndromic profiles of the neuropsychological and neurolinguistic tests have been reviewed and published previously[18 19].

In comparing testing environments, our null hypothesis was that there would be no effect of testing environment on neuropsychological performance – i.e., no differences in performance between remote and face-to-face assessment settings – for any participant group. To critically assess the magnitude of evidence in favour of this null hypothesis versus the alternative hypothesis (i.e., that there was in fact an effect of testing environment) particularly in light of the relatively small patient cohorts here, we employed a Bayesian approach[20]. Bayesian independent samples t-tests (and non-parametric equivalents where assumptions of the general linear model were violated) were performed for each general neuropsychological and neurolinguistic test in each patient group separately. As numbers in some groups were quite small, we also conducted analyses for a combined patient cohort in both environments. Healthy control performance was compared using Bayesian paired samples t-tests (or appropriate non-parametric equivalent). A Bayes factor, which is the ratio of evidence supporting the null hypothesis over the alternative hypothesis (hereafter BF_{01}) was calculated for each comparison using JASP. A BF_{01} value > 3 indicates substantial evidence in favour of the null hypothesis while a value < 0.33 supports the alternative hypothesis; BF_{01} values between 0.33 and 3 are classified as ‘anecdotal’ evidence, comparable to non-significant differences in inferential statistics[21 22]. Bayes factor values are presented in Tables S2 and S3 (Supplementary file).

In comparing groups on comfort ratings after the remote sessions, our null hypothesis was that there would be no differences in comfort ratings between healthy controls and patients; our alternative hypothesis was that healthy control participants would report higher comfort ratings than patients.

Finally, we conducted F-tests and Levene’s equality of variance tests to evaluate differences in variability between the two testing environments.

Patient and public involvement

For peer review only - http://bmjopen.bmj.com/site/about/guidelines.xhtml

In August and September 2020, we contacted 527 people (comprising healthy control participants and people with a diagnosis of a dementia) who had previously taken part in our face-to-face research programmes in the Dementia Research Centre, UCL, or who had expressed an interest in doing so in the future. They were asked, "Would you consider participating in research remotely (telephone/ online)?" Of the 163 people who answered the question, 145 (89%) indicated that they would be happy to take part in remote research. Based on this feedback, we submitted an amendment to our existing research ethics that was approved in October 2020. Following this, we conducted a pilot remote testing session with an older healthy control individual who was also a carer for a family member living with dementia. Their feedback was instrumental in developing and improving our remote testing procedure.

Results from this work will be disseminated to members of the support groups that we run with Rare Dementia Support (www.raredementiasupport.org) through online presentations at webinars and research summaries in newsletters.

Results

General characteristics of participant groups are presented in Table 1; performance on the general neuropsychological battery is given in Table 2; performance on the neurolinguistic battery is shown in Table 3. Figure 1 and Figure 2 show radar plots of performance for each participant group for the general neuropsychological and neurolinguistic batteries, respectively. Figure 3 and Figure 4 show performance profiles of healthy control participants on the general neuropsychological and neurolinguistics batteries, respectively. Figure S2 and Figure S3 show performance profiles of the combined patient cohort on the general neuropsychological and neurolinguistic batteries, respectively. Bayesian statistics are presented in Tables S2 and S3; equality of variance analyses are presented in Table S4; and results for the audibility screening task (see Supplementary methods for more information) are presented in Table S5.

For peer review only - http://bmjopen.bmj.com/site/about/guidelines.xhtml

Table 2. General neuropsychological performance for all participant groups: comparison of remote and face-to-face test administration

	CTL		All Patients		AD		bvFTD		SD		PNFA		LPA	
	**F2F**	**Remote**	**F2F**	**Remote**	**F2F**	**Remote**	**F2F**	**Remote**	**F2F**	**Remote**	**F2F**	**Remote**	**F2F**	**Remote**
**No. tested**	10	10	64	25	25	8	12	3	9	4	12	5	6	5
General intellect														
WASI Matrix (/32)	26.9 (2.4)	26.3 (3.5)	**16.5 (8.86)**	**17.7 (9.24)**	**14.5 (7.5)**	**13.7 (11.5)**[b]	14.6 (9.5)[a]	7.7 (2.5)	25.8 (4.9)	23.3 (3.4)	18.6 (8.3)[a]	24.0 (2.6)	10.0 (9.2)	17.6 (10.3)
Episodic memory														
RMT faces (Short) (/25)	NA	23.3 (2.5)	**18.9 (3.68)**	**19.2 (4.21)**	18.9 (3.7)[h]	15.3 (2.1)	NA	20.3 (4.0)	NA	19.3 (3.7)	NA	22.4 (3.7)	NA	21.6 (3.8)
RMT faces (/50)	42.7 (4.7)	NA	**32.6 (7.40)**	**NA**	30.44 (6.56)[g]	NA	28.56 (6.29)[c]	NA	31.5 (4.47)[a]	NA	40.3 (5.79)[b]	NA	33.17 (8.93)	NA
Working memory														
DS (Reverse) (/12)	**8.0 (2.6)**	**8.2 (2.2)**	**4.82 (2.47)**	**4.64 (2.40)**	4.6 (1.9)[c]	4.0 (1.9)	5.0 (3.1)[a]	4.0 (0.0)	6.9 (2.7)	5.5 (2.7)	4.4 (1.7)[d]	5.2 (4.2)	2.4 (1.1)[a]	4.8 (1.8)
Short-term verbal memory														
DS (Forward) (/12)	9.8 (2.0)	9.2 (2.0)	**6.68 (2.94)**	**6.24 (2.52)**	**6.5 (2.1)**[a]	**6.6 (2.3)**	7.8 (2.9)	6.3 (0.6)	9.4 (2.2)	7.3 (0.5)	5.1 (2.6)[b]	6.0 (4.4)	3.0 (3.1)[a]	5.0 (2.5)
Language														
BPVS (/150)	**149.0 (1.1)**	**148.9 (1.1)**	**120 (39.3)**	**134 (26.4)**	**135.1 (24.0)**[a]	**133.9 (21.4)**	118.7 (28.9)[a]	148.0 (1.0)	61.9 (44.1)	98.0 (46.3)	136.3 (30.4)	142.8 (8.4)	120.2 (39.6)	145.2 (4.4)
GNT (/30)	27.3 (2.1)	26.8 (1.8)	**11 (9.33)**	**13 (8.62)**	**13.7 (8.5)**[a]	**12.4 (6.3)**	11.5 (11.0)[a]	20.0 (4.4)	0.2 (0.4)	0.5 (1.0)	15.8 (6.8)	20.2 (7.6)	6.2 (7.7)	12.4 (7.0)
NART (/50)	45.2 (3.4)	44.6 (3.0)	26.1 (13.4)	31.4 (13.1)	28.3 (11.9)[a]	37.1 (4.6)	30.7 (13.1)	40.0 (2.0)	16.1 (10.2)	16.3 (13.6)	28.3 (15.0)[f]	30.8 (14.9)	20.4 (18.5)[a]	29.8 (16.9)
Category fluency (60s, “Animals”)	**25.0 (6.6)**	**24.7 (5.4)**	8.46 (5.75)	12.2 (8.10)	**10.0 (5.6)**	**10.4 (6.7)**	8.0 (6.1)	10.3 (6.0)	6.1 (5.3)[a]	9.8 (6.2)	8.6 (5.8)[d]	18.6 (11.5)	5.7 (5.8)	11.6 (8.1)
Arithmetic														
GDA Total (/24)	**16.6 (6.4)**	**16.1 (4.0)**	**6.90 (6.25)**	**6.30 (5.60)**	**4.9 (5.0)**[e]	**4.8 (5.4)**	8.2 (7.4)[a]	8.3 (7.2)	13.3 (5.5)	7.3 (4.0)	5 (3.6)[d]	6.4 (7.2)	1.7 (1.5)	7 (5.8)
Visuospatial														
VOSP OD (/20)	*19.5 (0.7)*	*18.4 (1.2)*	**15.6 (3.77)**	**15.2 (3.52)**	*16.1 (2.6)*[a]	*13.0 (3.0)*	14.0 (4.2)	17.0 (1.0)	15.3 (6.3)[a]	14.5 (5.9)	16.8 (3.6)[b]	17.6 (2.1)	15.8 (3.1)	15.8 (2.7)
Executive														
Letter fluency (60s, “F”)	**19.7 (6.0)**	**20.8 (4.2)**	7.70 (5.27)	11.3 (6.29)	10.3 (5.2)	11.9 (7.4)	6.8 (3.9)[a]	7.3 (2.3)	7.0 (3.6)[b]	10.8 (7.0)	4.8 (5.8)[d]	14.0 (8.2)[a]	*2.6 (3.8)*[a]	*11.3 (3.9)*

Mean (standard deviation) values of performance on neuropsychology tests are shown (maximum scores are indicated in parentheses) for each testing environment (face-to-face vs remote research setting) for each patient group. **Bold** indicates values for which there was substantial evidence in support of the null hypothesis (H0); *italics* indicate values for which there was substantial evidence in support of the alternative hypothesis (H1). Exact values for the Bayes factor comparing H0 against H1 (BF_{01}) are presented in Table S2 (Supplementary file). The general neuropsychological battery comprised tests of general intellect (Wechsler Adult Intelligence Scale (WASI) Matrix Reasoning[23]), episodic memory (Recognition Memory Test (RMT) for Faces[24]), working memory (Digit span forwards[25]), language (the graded naming test (GNT[26]), British Picture Vocabulary Scale (BPVS[27]) and National Adult Reading Test (NART)[28]), arithmetic (Graded Difficulty Arithmetic (GDA)[29]), visual perception (Visual Object and Space Perception (VOSP) Object Decision task[30]) and executive function (Digit span reverse[25], letter (‘F’) and category fluency (‘animal’) tasks[31]). A reduced number of participants completed certain tests, as follows: [a]n-1, [b]n-2, [c]n-3, [d]n-4, [e]n-5, [f]n-6, [g]n-9, [h]n-16. AD, patient group with typical Alzheimer’s disease; All Patients, combined patient cohort; bvFTD, patient group with behavioural variant frontotemporal

For peer review only - http://bmjopen.bmj.com/site/about/guidelines.xhtml

dementia; CTL, healthy control group; F2F, face-to-face; LPA, patient group with logopenic progressive aphasia; NA, not available; PNFA, patient group with progressive nonfluent aphasia; SD, patient group with semantic dementia.

For peer review only

Table 3. Neurolinguistic performance for all participant groups: comparison of face-to-face and remote test administration

Test	CTL		All Patients		SD		PNFA		LPA	
	F2F	Remote	F2F	Remote	F2F	Remote	F2F	Remote	F2F	Remote
**No. tested**	10	10	27	14	9	4	12	5	6	5
Phoneme perception										
PALPA 3 (/36)	34.9 (1.6)	33.8 (2.0)	33.5 (4.28)	33.1 (2.85)	35.3 (1.3)	33.5 (2.6)	31.9 (5.7)	34.0 (1.6)	33.8 (2.4)[a]	32.0 (4.0)
Reading										
Non word reading (/25)	24.4 (0.8)	24.9 (0.3)	**18.1 (5.51)**	**16.1 (8.58)**	19.6 (4.9)	16.0 (9.8)	19.3 (5.7)[d]	15.8 (8.7)	14.5 (5.4)	16.4 (9.6)
Regular reading (/25)	25.0 (0.0)	25.0 (0.0)	23.3 (2.83)	21.8 (5.07)	22.8 (3.9)[d]	21.3 (6.2)	23.3 (2.2)[g]	22.2 (4.2)	24.5 (0.7)[d]	21.8 (6.1)
Irregular reading (/25)	24.7 (0.7)	25.0 (0.0)	19.6 (5.30)	21 (5.39)	18.8 (5.8)[d]	18.3 (8.9)	19.8 (6.4)[g]	21.6 (4.4)	21.5 (3.5)[d]	22.6 (2.1)
Naming										
BNT (/30)	**29.2 (0.8)**	**29.0 (1.0)**	13.4 (10.9)	17.4 (9.58)	3.3 (3.4)	9.5 (7.1)	24.7 (4.4)[b]	24.4 (7.6)	9.7 (8.0)	16.8 (8.8)
Semantic association										
Camel and cactus (/32)	**31.0 (1.0)**	**30.9 (1.1)**	25.4 (5.58)	28.4 (2.71)	22.3 (5.1)[e]	25.5 (2.6)	28.3 (5.5)[h]	30.0 (1.4)	29.0 (0.0)[e]	29.0 (2.1)
Word comprehension										
Concrete synonyms (/25)	24.5 (0.5)	24.3 (0.5)	**18.7 (5.99)**	**20.2 (4.00)**	15.5 (2.9)[c]	17.0 (4.0)[a]	22.2 (3.2)[b]	21.0 (4.2)	15.6 (9.4)[a]	21.4 (3.5)
Abstract synonyms (/25)	24.8 (0.4)	24.3 (0.8)	18.5 (6.20)	20.6 (3.12)	14.4 (1.5)[d]	17.3 (1.2)[a]	21.4 (3.7)[b]	21.2 (3.4)	16.5 (11.2)[b]	22.3 (2.1)[a]
Sentence comprehension										
PALPA 55 (/24)	23.8 (0.4)	23.1 (1.3)	**20.5 (3.83)**	**20.9 (2.28)**	22.6 (1.6)[a]	21.8 (2.2)	19.6 (4.8)[a]	21.0 (2.6)	18.8 (2.6)[a]	20.0 (2.1)
Speech repetition										
Monosyllabic word repetition (/15)	*14.6 (0.5)*	*12.7 (1.6)*	13.8 (1.92)	10.6 (3.37)	14.8 (0.4)	10.8 (3.2)	12.8 (2.6)[c]	10.0 (4.2)	14.0 (1.2)[b]	11.2 (3.3)
Bisyllabic word repetition (/15)	**14.7 (0.5)**	**14.7 (0.7)**	**13.5 (2.65)**	**13.4 (2.95)**	14.8 (0.7)	13.0 (1.6)	12.8 (3.3)[c]	12.4 (4.7)	12.0 (2.9)[b]	14.8 (0.4)
Trisyllabic word repetition (/15)	15.0 (0.0)	15.0 (0.0)	**12.8 (4.18)**	**12.9 (3.89)**	14.8 (0.4)	12.8 (3.2)	11.1 (5.9)[c]	12.0 (6.2)	12.3 (2.9)[b]	14.0 (1.0)
Graded difficulty sentence repetition (/10)	9.8 (0.4)	9.3 (0.9)	6.37 (2.89)	5.79 (2.36)	8.3 (1.2)	7.3 (1.7)	4.6 (3.0)[e]	5.2 (3.2)	4.7 (2.9)[c]	5.2 (1.6)
Sentence construction										
Spoken sentences (/25)	25.0 (0.0)	24.9 (0.3)	18.6 (6.70)	22.6 (3.34)	19.1 (7.5)	23.5 (1.3)	18.0 (7.4)[d]	21.6 (5.1)	18.0 (5.3)[c]	22.8 (2.7)

Mean (standard deviation) values of performance on neurolinguistic tests are shown (maximum scores are indicated in parentheses) for each testing environment (face-to-face vs remote) for each participant group. **Bold** indicates values for which there was substantial evidence in support of the null hypothesis (H0); *italics* indicate values for which there was substantial evidence in support of the alternative hypothesis (H1). Exact values for the Bayes factor comparing H0 against H1 (BF_{01}) are presented in Table S3 (Supplementary file). The neurolinguistic battery comprised tests of phoneme perception (a shortened version of the Psycholinguistic Assessments of Language Processing in Aphasia (PALPA) Test 3 – minimal pair discrimination[32]), reading (graded non-word reading[33], and graded tests of regular word and irregular word reading adapted from[34]), confrontation naming (a subset of items from the Boston Naming Test (BNT)[35]), semantic association (modified Camel and Cactus test[36]), single word comprehension (concrete and abstract synonyms[37]), sentence comprehension (a shortened version of the PALPA Subtest 55[32]), speech repetition (tests of monosyllabic, bisyllabic, and trisyllabic single word repetition[38], graded difficulty sentence repetition using a subset of items from the Sentence Repetition test[39] and sentence construction based on[40]. We opted to include two naming tasks as the GNT is part of the core neuropsychological battery at our Centre and therefore administered to patients with all diagnoses. However, as patients with SD often score at floor on this task[41], we also include a shortened version of the BNT in our neurolinguistic battery that is administered to patients with PPA as SD patients are more likely to

For peer review only - http://bmjopen.bmj.com/site/about/guidelines.xhtml

be above floor on this task[42]. A reduced number of participants completed certain tests: [a]n-1, [b]n-2, [c]n-3, [d]n-4, [e]n-5, [f]n-6, [g]n-8, [h]n-9. In the spoken sentence construction test, participants are given five different words (walked, radio, throw, green, tree) and are asked to produce a sentence that contains each word in turn. Sentences are then scored based on grammar and semantic sense, each sentence counting for a maximum of 5 points. All Patients, combined patient cohort; CTRL, healthy control group; F2F, face-to-face; LPA, patient group with logopenic progressive aphasia; N, number of participants per group; PNFA, patient group with progressive nonfluent aphasia; SD, patient group with semantic dementia.

For peer review only

For peer review only - http://bmjopen.bmj.com/site/about/guidelines.xhtml

General participant characteristics

Of the 87 potential participants who were contacted, 35 (40.2%) ultimately took part in the research (25 patients, 10 healthy controls who had taken part in face-to-face research previously). Reasons for declining participation and reports of any technical issues with remote test delivery are detailed in Supplementary Material online.

There were no significant differences in age, years of education or symptom duration between the face-to-face and remote testing patient cohorts (Table 1).

Below we highlight comparisons where there was substantial evidence in support of either the null (i.e. no difference between remote and face-to-face performance) or alternative (i.e. difference between remote and face-to-face performance) hypothesis. Comparisons are shown in full in Tables S2 and S3 (Supplementary file).

General neuropsychological assessment

Overall, there was little evidence for a significant effect of assessment environment on general neuropsychological test performance in any participant group.

Healthy individuals scored equally well on the digit span reverse, the British Picture Vocabulary Scale (BPVS), the Graded Difficulty Arithmetic (GDA), and on both letter and category fluency tests (all $BF_{01}>3$ indicating substantial evidence in favour of the null hypothesis). However, they performed less well on the Visual Object and Spatial Perception Object Decision task (VOSP) ($BF_{01}=0.0404$, indicating substantial evidence in favour of the alternative hypothesis) in remote testing than in face-to-face testing, with the remote group (mean=18.4) performing worse than the face-to-face group (mean=19.5) (Tables 2 and S2, Figure 1 and Figure 3).

For the comparisons of the combined remote vs combined face-to-face patient cohorts, there was substantial evidence supporting the null hypothesis for all neuropsychological tests (all $BF_{01}>3$), except for the NART and both letter and category fluency tests, where evidence in support of the null hypothesis was anecdotal (Tables 2 and S2).

For individual patient groups (Tables 2 and S2, Figure 1), there was substantial evidence to suggest that the remote AD cohort performed similarly to the face-to-face AD cohort on WASI matrix reasoning, digit span forward, graded naming test (GNT), BPVS, GDA, and category fluency test (all $BF_{01}>3$). However, the remote AD cohort performed less well on the VOSP (mean=13.0; $BF_{01}=0.171$, substantial evidence) compared to the face-to-face cohort (mean=16.1). Conversely, LPA patients who completed the letter fluency test remotely (mean words=11.3) performed better than those who completed the same task face-to-face (mean=2.6; BF01=0.188, substantial evidence).

No other comparisons yielded substantial evidence in support of either hypothesis (Table S2, Supplementary file).

For peer review only - http://bmjopen.bmj.com/site/about/guidelines.xhtml

Results of the equality of variance analyses are reported in full in Supplementary materials online (Table S4). The assumption of homogeneity of variance was not violated for any test in the general neuropsychological battery in either the healthy control or combined patient cohorts.

Neurolinguistic assessment

Overall, there was little evidence for a significant effect of assessment environment on neurolinguistic test performance in any participant group.

Healthy individuals scored equally well on the Boston Naming Test (BNT), the camel and cactus test, and the bisyllabic single word repetition test (all $BF_{01}>3$, indicating substantial evidence in favour of the null hypothesis). However, they performed less well on the monosyllabic word repetition test ($BF_{01}=0.0487$, substantial evidence in favour of the alternative hypothesis) in remote testing than in face-to-face testing, with the remote group (mean=12.7) performing worse than the face-to-face group (mean=14.6) (Tables 3 and S3, Figure 2 and Figure 4).

The comparisons between combined patient cohorts for remote vs face-to-face testing showed substantial evidence supporting the null hypothesis for non-word reading, concrete synonyms, the PALPA-55, and bisyllabic and trisyllabic single word repetition tests (all BF_{01} values>3). There was anecdotal evidence supporting the null hypothesis on all other neurolinguistic tests (all BF_{01} values between 1 and 3).

Individual patient group comparisons across environments did not yield substantial evidence in support of either hypothesis.

Results of the equality of variance analyses are reported in full in Supplementary materials online (Table S4). For the healthy control group, the assumption of equality of variance was violated for mono-syllabic repetition ($F=0.11$, $p<0.05$, with higher variability in the remote condition); for the combined patient cohort this assumption was violated for mono-syllabic repetition ($F=4.89$, $p=0.03$, with higher variability in the remote group), the camel and cactus test ($F=4.25$, $p=0.02$, with higher variability in the face-to-face group), and the spoken sentence task ($F=4.37$, $p<0.05$, with higher variability in the face-to-face group).

Feedback on remote testing experience

We received 20 responses to the question, “How comfortable did you feel in this new setting (e.g. online testing?”) after the general neuropsychology remote session (from 10 healthy control participants and 10 patients); and 22 responses to the same question that was posed after the neurolinguistic remote session (from 10 healthy control participants and 12 patients). There was little evidence for a significant difference across groups on these ratings for either the general neuropsychology session (control mean = 9.60, standard deviation = 0.70; patient mean = 9.10, standard deviation = 1.91; $BF_{01}=1.825$, anecdotal evidence supporting the null hypothesis) or neurolinguistic session (control mean = 9.10, standard deviation = 1.73; patient mean = 8.92, standard deviation = 1.73; $BF_{01}=2.325$, anecdotal evidence supporting the null hypothesis).

For peer review only - http://bmjopen.bmj.com/site/about/guidelines.xhtml

Discussion

The present findings suggest that administration of neuropsychological tasks remotely over the internet with healthy older adults and people with a diverse range of dementia phenotypes is feasible according to three key metrics: acceptability, adaptation, and demand[11]. In terms of acceptability, results from our feedback questionnaires indicated that patient and healthy control participants were comfortable with the remote testing environment. For adaptation, we have demonstrated that similar performance outcomes were obtained across test settings (with further discussion of these findings below). And in terms of demand, only 6.9% of those we contacted about taking part in the research declined due to technological reasons (Supplementary Materials online).

Our Bayesian analytical approach demonstrated that there was anecdotal or substantial evidence suggesting comparable performance across testing environments of healthy participants and AD patients on a range of general neuropsychological and neurolinguistic tests, specifically those targeting working memory (digit span forward), executive functioning (digit span reverse, letter and category fluency tests, WASI matrix reasoning), arithmetic skills (GDA), and general semantic knowledge (BNT, BPVS, GNT). These results corroborate previous reports of preserved neuropsychological performance on executive function, working memory, and language tests across testing environments in both healthy individuals and AD patients[10]. Our findings also corroborated previous work suggesting that remote assessments are viable for people with PPA[43].

Healthy controls and AD participants both performed significantly worse on the remote version of the VOSP object decision task, in which participants are presented with four silhouettes and asked to select the drawing of a real object; the three distractor silhouettes are based on nonsense shapes. The typical amnestic AD phenotype can include prominent visuospatial impairments[44 45] and it is feasible that a reduction in stimulus quality may have stressed cortical apperceptive mechanisms still further, akin to a dynamic 'stress test' of degraded input processing[42 46 47]. However, it is worth noting that there was no such discrepancy across the AD cohorts for other tasks involving visual administration (e.g., WASI Matrix). For the healthy controls, the absolute performance difference across environments was relatively small (mean reduction of 1.1 points) for the VOSP; however we note that even small differences can have important consequences if this change were to yield a lower scaled/percentile score and thus affect interpretations of test performance. It is also possible that this reduction at least in part reflected normal healthy ageing, consistent with previous findings[48], as the healthy control cohort was tested on the remote battery three-to-four years after their face-to-face assessment.

Healthy control participants also performed significantly worse on the monosyllabic single word repetition task when delivered remotely, and there was more variability on this task when performed remotely. The videoconferencing software may have degraded the fidelity of the raw speech signal[49], essentially resulting in a harder task than when administered face-to-face. This is potentially consistent with the controls' preserved performance on the bisyllabic single word repetition test where top-down information can be used to complement bottom-up auditory information partially degraded by the videoconferencing software[50]. An alternative (or complementary) explanation could again be age-related changes, here affecting hearing function (presbycusis)[51-53].

For peer review only - http://bmjopen.bmj.com/site/about/guidelines.xhtml

The finding of significantly better performance on the verbal (letter) fluency task in the LPA cohort tested remotely compared to the cohort tested face-to-face is surprising. The obvious explanation is that the remote cohort was overall less impaired than the patients seen face-to-face; although efforts were made to match the two cohorts for disease severity and other potentially relevant factors. An alternative explanation could be that participants found the remote setting less anxiety-provoking than face-to-face testing in an unfamiliar environment. Patients with LPA may be relatively susceptible to anxiety as a factor modulating cognitive performance[54 55]. Additionally, as word retrieval is an intrinsically dynamic process that is likely to be facilitated by the availability of 'prompts'[56], patients may have benefitted from cueing of word retrieval by their more familiar home environments.

The way the neuropsychology and neurolinguistic testing protocol was adapted for remote delivery may have favoured null differences. The testing sessions were shorter and spread out within a week, which may have helped counteract the effect of anxiety related to the unfamiliarity of the remote testing setting, as well as potential 'Zoom' fatigue[17]. The increased flexibility of scheduling compared to face-to-face testing in addition to the absence of potentially stressors associated with a face-to-face research visit (e.g., travelling, being in a unfamiliar environment) may have led to participants feeling more relaxed when taking part remotely vs face-to-face: future research should explore this. Furthermore, certain tests selected for remote delivery may have been intrinsically less susceptible to changes in testing protocol (e.g., BPVS), whereas we deliberately excluded tests that we considered would not be practical or suboptimal for remote delivery (e.g., WASI Block design, Baxter spelling test, Trails). Anecdotally, participants reported satisfaction with the remote testing protocol.

The current study presents several limitations which should inform future work. First, while most statistical comparisons indicated similar performance between testing environments for healthy and dementia participants, they were not all supported by substantial evidence and certain comparisons even led to the opposite conclusion. Second, the present study was not ideally designed to compare the two testing environments, as the patient cohorts were different and the healthy control participants were not tested simultaneously in both environments within the same year. This meant that certain statistical measures that may have been informative (e.g. assessing stability of ranking within groups) were not appropriate here. While it is likely that the variability of test results in the patient cohorts will be influenced by testing environment, this needs to be interpreted cautiously, due to unequal sample sizes and individual disease trajectories - and may further depend on the particular test employed. Indeed, our sample sizes across modalities were relatively small. These findings would need to be replicated in larger cohorts with the same patients in each test situation, to rule out the possibility of small differences observed in favour of face-to-face testing – and in particular, to assess the extent of individual variability in any differential effect of test environment. Patients of equivalent disease severity would also need to be tested to compare the differential impact of diagnosis on remote performance over the course of the illness. Third, here we did not control for potential deficits in peripheral hearing as these are difficult to measure remotely without adequate equipment. Fourth, we manually adapted face-to-face tasks for remote administration, but there are now several established fully integrated online neuropsychological test batteries that have shown success in assessing patients with neurodegenerative disease remotely[57]: future research could explore the extent to which our results are comparable with those obtained by such batteries. Fifth, only a proportion of patients had time to respond to the additional question asking for their rating of comfort with the remote testing setup, and it is possible that there was some selection bias here in that those patients who felt most comfortable with the technology finished the sessions earlier and therefore had time to give this additional

For peer review only - http://bmjopen.bmj.com/site/about/guidelines.xhtml

feedback. Relatedly, whilst there was little evidence for a significant effect of 'group' (controls vs patients) on these comfort ratings, the patient cohort did record a lower mean average score than controls after both the remote general neuropsychological and neurolinguistic sessions, something that would warrant further and more in-depth investigation with validated assessment tools. Finally, we note that the study was designed to explore the potential for remote neuropsychological assessments of research participants, and the results and conclusions here may not generalise to clinical settings.

Overall, the present findings demonstrate that, despite challenges in setting up remote testing protocols (specifically due to technological requirements), these may produce similar results to face-to-face testing protocols. These are encouraging findings given the current climate and anticipating that research participants may continue to favour remote (or hybrid) visits over face-to-face assessments for reasons of convenience as well as safety, as we move beyond the COVID-19 pandemic.

For peer review only

Acknowledgements

We are grateful to all participants for their involvement.

Funding

The Dementia Research Centre is supported by Alzheimer's Research UK, Brain Research UK, and The Wolfson Foundation. This work was supported by the Alzheimer's Society, the Royal National Institute for Deaf People, Alzheimer's Research UK, the National Institute for Health Research University College London Hospitals Biomedical Research Centre, and the University College London Leonard Wolfson Experimental Neurology Centre (grant PR/ylr/18575). MCRK was supported by a Wellcome Trust PhD Studentship (102129/B/13/Z). JJ is supported by a Frontotemporal Dementia Research Studentship in Memory of David Blechner (funded through The National Brain Appeal). EB was supported by a Brain Research UK PhD Studentship. RLB was supported by an MRC PhD Studentship in Mental Health. SJC was supported by grants from ESRC-NIHR (ES/L001810/1), EPSRC (EP/M006093/1) and Wellcome Trust (200783). CJDH was supported by a Royal National Institute for Deaf People–Dunhill Medical Trust Pauline Ashley Fellowship (grant PA23_Hardy) and a Wellcome Institutional Strategic Support Fund Award (204841/Z/16/Z).

This research was funded in part by UKRI and the Wellcome Trust (Grant 204841/Z/16/Z). For the purpose of Open Access, the author has applied a Creative Commons Attribution (CC BY) public copyright licence to any Author Accepted Manuscript version arising from this submission.

Competing interests

The authors have no competing interests to declare.

Data availability statement

The data that support the findings of this study are available on request from the corresponding author.

Contributors

MCRK, JJ, LD, EB, LR, RLB, EVB, CG, SB, JDR, SJC, JDW and CJDH contributed to the conception and design of the study. MCRK, JJ, LD, EB, LR, RLB, EVB, CG and CJDH contributed to the acquisition of data. MCRK, JJ, JDW and CJDH were involved in the analysis and interpretation of data, and wrote the first draft of the article. MCRK, JJ, LD, EB, LR, RLB, EVB, CG, SB, JDR, SJC, JDW and CJDH were involved in revision of the draft and approved the final version of the manuscript.

For peer review only - http://bmjopen.bmj.com/site/about/guidelines.xhtml

Ethics approval

Ethical approval was granted by the University College London and National Hospital for Neurology and Neurosurgery Joint Research Ethics Committees in accordance with the Declaration of Helsinki (Refs 06N032 and 150508).

For peer review only

For peer review only - http://bmjopen.bmj.com/site/about/guidelines.xhtml

References

1. Wang QQ, Davis PB, Gurney ME, et al. COVID-19 and dementia: Analyses of risk, disparity, and outcomes from electronic health records in the US. *Alzheimers Dement* 2021;17(8):1297-306. doi: 10.1002/alz.12296
2. Poon P, Hui E, Dai D, et al. Cognitive intervention for community-dwelling older persons with memory problems: telemedicine versus face-to-face treatment. *Int J Geriat Psychiatry* 2005;20(3):285-6. doi: 10.1002/gps.1282
3. Jelcic N, Agostini M, Meneghello F, et al. Feasibility and efficacy of cognitive telerehabilitation in early Alzheimer's disease: a pilot study. *Clin Interv Aging* 2014;9:1605-11. doi: 10.2147/CIA.S68145
4. Costanzo MC, Arcidiacono C, Rodolico A, et al. Diagnostic and interventional implications of telemedicine in Alzheimer's disease and mild cognitive impairment: a literature review. *Int J Geriatr Psychiatry* 2020;35(1):12-28.
5. Dial HR, Hinshelwood HA, Grasso SM, et al. Investigating the utility of teletherapy in individuals with primary progressive aphasia. *Clin Interv Aging* 2019;14:453-71. doi: 10.2147/CIA.S178878
6. Rogalski EJ, Saxon M, McKenna H, et al. Communication Bridge: A pilot feasibility study of Internet-based speech-language therapy for individuals with progressive aphasia. *Alzheimers Dement* 2016;2(4):213-21. doi: 10.1016/j.trci.2016.08.005
7. Capozzo R, Zoccolella S, Frisullo ME, et al. Telemedicine for Delivery of Care in Frontotemporal Lobar Degeneration During COVID-19 Pandemic: Results from Southern Italy. *J Alz Dis* 2020;76(2):481-9. doi: 10.3233/JAD-200589
8. Cuffaro L, Di Lorenzo F, Bonavita S, et al. Dementia care and COVID-19 pandemic: a necessary digital revolution. *Neurol Sci* 2020;41(8):1977-9. doi: 10.1007/s10072-020-04512-4
9. Waddington CG, Harding E, Brotherhood EV, et al. The Development of Virtual Videoconference-Based Support for People Living with Rare Dementias and Their Carers: Three-Phase Support Group Evaluation Protocol. *JMIR Research Protocols* in press
10. Hunter M, Jenkins N, Dolan C, et al. Reliability of Telephone and Videoconference Methods of Cognitive Assessment in Older Adults with and without Dementia. *J Alz Dis* 2021;81:1625-47. doi: 10.3233/JAD-210088
11. Bowen DJ, Kreuter M, Spring B, et al. How We Design Feasibility Studies. *American Journal of Preventive Medicine* 2009;36(5):452-57. doi: https://doi.org/10.1016/j.amepre.2009.02.002
12. Newkirk LA, Kim JM, Tinklenberg JR, et al. Validation of a 26-point telephone version of the Mini-Mental State Examination. *J Geriatr Psychiatry Neurol* 2004;17(2):81-7. doi: 10.1177/0891988704264534
13. Kennedy RE, Williams CP, Sawyer P, et al. Comparison of In-Person and Telephone Administration of the Mini-Mental State Examination in the University of Alabama at Birmingham Study of Aging. *J Am Geriatr Soc* 2014;62(10):1928-32. doi: 10.1111/jgs.13027
14. Dubois B, Feldman HH, Jacova C, et al. Advancing research diagnostic criteria for Alzheimer's disease: the IWG-2 criteria. *Lancet Neurol* 2014;13:614-29.
15. Rascovsky K, Hodges JR, Knopman D, et al. Sensitivity of revised diagnostic criteria for the behavioural variant of frontotemporal dementia. *Brain* 2011;134:2456-77.
16. Gorno-Tempini ML, Hillis AE, Weintraub S, et al. Classification of primary progressive aphasia and its variants. *Neurology* 2011;76:1006-14.
17. Bailenson JN. Nonverbal overload: A theoretical argument for the causes of Zoom fatigue. *TMB* 2021;2(1) doi: 10.1037/tmb0000030
18. Sivasathiaseelan H, Marshall CR, Agustus JL, et al. Frontotemporal Dementia: A Clinical Review. *Semin Neurol* 2019;39(2):251-63. doi: 10.1055/s-0039-1683379
19. Marshall CR, Hardy CJD, Volkmer A, et al. Primary progressive aphasia: a clinical approach. *J Neurol* 2018;265(6):1474-90. doi: 10.1007/s00415-018-8762-6 [published Online First: 2018/02/03]

For peer review only - http://bmjopen.bmj.com/site/about/guidelines.xhtml

20. Dienes Z. Using Bayes to get the most out of non-significant results. *Front Psychol* 2014;5:781. doi: 10.3389/fpsyg.2014.00781
21. Quintana DS, Williams DR. Bayesian alternatives for common null-hypothesis significance tests in psychiatry: a non-technical guide using JASP. *BMC Psychiatry* 2018;18:178. doi: 10.1186/s12888-018-1761-4
22. Ashworth M, Palikara O, Burchell E, et al. Online and Face-to-Face Performance on Two Cognitive Tasks in Children With Williams Syndrome. *Front Psychol* 2021;11:594465. doi: 10.3389/fpsyg.2020.594465
23. Wechsler D. Wechsler Adult Intelligence Scale-Revised. New York: Psychological Corporation 1981.
24. Warrington EK. Recognition memory test. Windsor: NFER-Nelson 1984.
25. Wechsler D. Wechsler memory scale: Revised. San Antonio, TX: The Psychological Corporation 1987.
26. McKenna P, Warrington EK. Testing for nominal dysphasia. *J Neurol Neurosurgery, Psychiatry* 1987;43:781-8.
27. Dunn LM, Whetton C. British Picture Vocabulary Scale. Windsor, England: NFER-Nelson 1997.
28. Nelson HE, Wilson J. National adult reading test (NART). Windsor, UK: Nfer-Nelson 1991.
29. Jackson M, Warrington EK. Arithmetic Skills in Patients with Unilateral Cerebral Lesions. *Cortex* 1986;22(4):611-20. doi: https://doi.org/10.1016/S0010-9452(86)80020-X
30. Warrington EK, James M. The visual object and space perception battery. Bury St Edmunds, UK: Thames Valley Test Company 1991.
31. Delis DC, Kaplan E, Kramer JH. Delis-Kaplan executive function system (D-KEFS). San Antonio, TX: Psychological Corporation 2001.
32. Kay J, Lesser R, Coltheart M. Psycholinguistic Assessments of Language Processing in Aphasia. 1992
33. Snowling M, Stothard J, McLean JF. Graded nonword reading test. *Thames Valley Test* 1996
34. Coltheart M, Besner D, Jonasson JT, et al. Phonological encoding in the lexical decision task. *Q J Exp* 1979;31
35. Kaplan E, Goodglass H, Weintraub S. Boston Naming Test. 1983
36. Moore K, Convery R, Bocchetta M, et al. A modified Camel and Cactus Test detects presymptomatic semantic impairment in genetic frontotemporal dementia within the GENFI cohort. *Appl Neuropsychol Adult* 2022;29(1):112-9. doi: 10.1080/23279095.2020.1716357
37. Warrington EK, Mckenna P, Orpwood L. Single Word Comprehension: A Concrete and Abstract Word Synonym Test. *Neuropsychol Rehabil* 1998;8(2):143-54.
38. McCarthy R, Warrington EK. A two-route model of speech production: evidence from aphasia. *Brain* 1984;107(2):463-85. doi: 10.1093/brain/107.2.463
39. Strauss E, Sherman EMS, Spreen O. A compendium of neuropsychological tests: Administration, norms, and commentary: Oxford University Press 2006.
40. Rohrer JD, Crutch SJ, Warrington EK, et al. Progranulin-associated primary progressive aphasia: A distinct phenotype? *Neuropsychologia* 2010;48(1):288-97. doi: 10.1016/j.neuropsychologia.2009.09.017
41. Hardy CJ, Agustus JL, Marshall CR, et al. Behavioural and neuroanatomical correlates of auditory speech analysis in primary progressive aphasias. *Alzheimer's Research and Therapy* 2017;9(53) doi: 10.1186/s13195-017-0278-2
42. Hardy CJ, Marshall CR, Bond RL, et al. Retained capacity for perceptual learning of degraded speech in primary progressive aphasia and Alzheimer's disease. *Alzheimers Res Ther* 2018;10(1):70. doi: 10.1186/s13195-018-0399-2
43. Rao LA, Roberts AC, Schafer R, et al. The Reliability of Telepractice Administration of the Western Aphasia Battery-Revised in Persons With Primary Progressive Aphasia. *Am J Speech Lang Pathol* 2022:1-15. doi: 10.1044/2021_AJSLP-21-00150
44. Day BL, Ocal D, Peters A, et al. Altered visual and haptic verticality perception in posterior cortical atrophy and Alzheimer's disease. *J Physiol* 2022;600(2):373-91. doi: 10.1113/JP282289

For peer review only

For peer review only - http://bmjopen.bmj.com/site/about/guidelines.xhtml

45. Mandal PK, Joshi J, Saharan S. Visuospatial perception: an emerging biomarker for Alzheimer's disease. *J Alz Dis* 2012;31 Suppl 3:S117-35. doi: 10.3233/JAD-2012-120901
46. Zarkali A, Adams RA, Psarras S, et al. Increased weighting on prior knowledge in Lewy body-associated visual hallucinations. *Brain Comms* 2019;1(1):fcz007. doi: 10.1093/braincomms/fcz00777
47. Weil RS, Pappa K, Schade RN, et al. The cats-and-dogs test: a tool to identify visuoperceptual deficits in Parkinson's disease. *Mov Disord* 2017;32(12):1789-90. doi: 10.1002/mds.27176
48. Herrera-Guzmán I, Peña-Casanova J, Lara JP, et al. Influence of Age, Sex, and Education on the Visual Object and Space Perception Battery (VOSP) In a Healthy Normal Elderly Population. *The Clinical Neuropsychologist* 2004;18(3):385-94. doi: 10.1080/1385404049052421
49. Weerathunge HR, Segina RK, Tracy L, et al. Accuracy of Acoustic Measures of Voice via Telepractice Videoconferencing Platforms. *J Speech Lang Hear Res* 2021;64(7):2586-99. doi: 10.1044/2021_JSLHR-20-00625
50. Jiang J, Benhamou E, Waters S, et al. Processing of Degraded Speech in Brain Disorders. *Brain Sci* 2021;11(3):394. doi: 10.3390/brainsci11030394
51. Goodwin MV, Hogervorst E, Maidment DW. The impact of presentation modality on cognitive test performance for adults with hearing loss. *Alzheimers Dement* 2021;17(S12):e058571. doi: 10.1002/alz.058571
52. Helfer KS, Mamo SK, Clauss M, et al. Listening in 2020: A Survey of Adults' Experiences With Pandemic-Related Disruptions. *Am J Audiol* 2021;30(4):941-55. doi: 10.1044/2021_AJA-21-00021
53. Naylor G, Burke LA, Holman JA. Covid-19 Lockdown Affects Hearing Disability and Handicap in Diverse Ways: A Rapid Online Survey Study. *Ear Hear* 2020;41(6):§442-9. doi: 10.1097/AUD.0000000000000948
54. Rohrer JD, Warren JD. Phenomenology and anatomy of abnormal behaviours in primary progressive aphasia. *J Neurol Sci* 2010;293:35-8. doi: 10.1016/j.jns.2010.03.012
55. Magnin E, Chopard G, Ferreira S, et al. Initial neuropsychological profile of a series of 20 patients with logopenic variant of primary progressive aphasia.
. *J Alzheimers Dis* 2013;36(4):799-808. doi: 10.3233/JAD-122335
56. Birn RM, Kenworthy L, Case L, et al. Neural systems supporting lexical search guided by letter and semantic category cues: a self-paced overt response fMRI study of verbal fluency. *Neuroimage* 2010;49(1):1099-107. doi: 10.1016/j.neuroimage.2009.07.036
57. Alosco ML, Mariani ML, Adler CH, et al. Developing methods to detect and diagnose chronic traumatic encephalopathy during life: rationale, design, and methodology for the DIAGNOSE CTE Research Project. *Alzheimers Res Ther* 2021;13(1):136. doi: 10.1186/s13195-021-00872-x

Figure legends

Figure 1. Radar plots of general neuropsychology battery performance, by participant group and testing environment

Average percentage correct score (plotted on concentric lines) was calculated for each participant group for each test in the neuropsychology battery, across each testing environment. Scores for the fluency tasks were not included here as responses on these tasks cannot be evaluated as correct/incorrect. AD, patient group with typical Alzheimer’s disease; BPVS, British Picture Vocabulary Scale; bvFTD, patient group with behavioural variant frontotemporal dementia; DS_For/Back, Digit Span Forwards/Backwards; GDA, Graded Difficulty Arithmetic test; GNT, Graded Naming Test; LPA, patient group with logopenic progressive aphasia; Matrix, WASI Matrix Reasoning; NART, National Adult Reading Test;

For peer review only - http://bmjopen.bmj.com/site/about/guidelines.xhtml

PNFA, patient group with progressive nonfluent aphasia; RMT, Recognition Memory Test; SD, patient group with semantic dementia; VOSP, Visual Object Space Perception battery.

Figure 2. Radar plots of performance on neurolinguistic battery performance, by participant group and testing environment

Average percentage score (plotted on concentric lines) was calculated for each participant group for each test in the neurolinguistic battery, across each testing environment. Abstract, abstract synonyms test; bi rep, bisyllabic single word repetition; BNT, Boston Naming Test; C & C, camel and cactus test; concrete, concrete synonyms test; irregular, irregular word reading test; LPA, patient group with logopenic progressive aphasia; mono rep, monosyllabic single word repetition test; non word, non-word reading test; PALPA, Psycholinguistic Assessment of Language Processing in Aphasia subtests; PNFA, patient group with progressive nonfluent aphasia; regular, regular word reading test; sentence rep, graded difficulty sentence repetition test; SD, patient group with semantic dementia; spoken sentences, spoken sentences test; tri rep, trisyllabic single word repetition test.

Figure 3. Performance profiles of healthy control participants on tasks in general neuropsychological battery

Line plots showing performance profiles of individual healthy control participants on tasks in the general neuropsychological battery. BPVS, British Picture Vocabulary Scale; GDA, Graded Difficulty Arithmetic test; GNT, Graded Naming Test; Matrix, WASI Matrix Reasoning; NART, National Adult Reading Test; VOSP, Visual Object Space Perception Object Decision task.

Figure 4. Performance profiles of healthy control participants on tasks in the neurolinguistic battery

Line plots indicating percentage scores for each healthy control on representative tests from the neurolinguistic battery administered face-to-face and remotely. Scores on the trisyllabic single word repetition task were jittered slightly on the x-axis to allow for plotting as participants were uniformly at ceiling in both environments. Bi rep, bisyllabic single word repetition; BNT, Boston Naming Test; Concrete, concrete synonyms test; F2F, face-to-face; Mono rep, monosyllabic single word repetition test; Non word, non-word reading test; PALPA, Psycholinguistic Assessment of Language Processing in Aphasia; Tri rep, trisyllabic single word repetition.

For peer review only - http://bmjopen.bmj.com/site/about/guidelines.xhtml

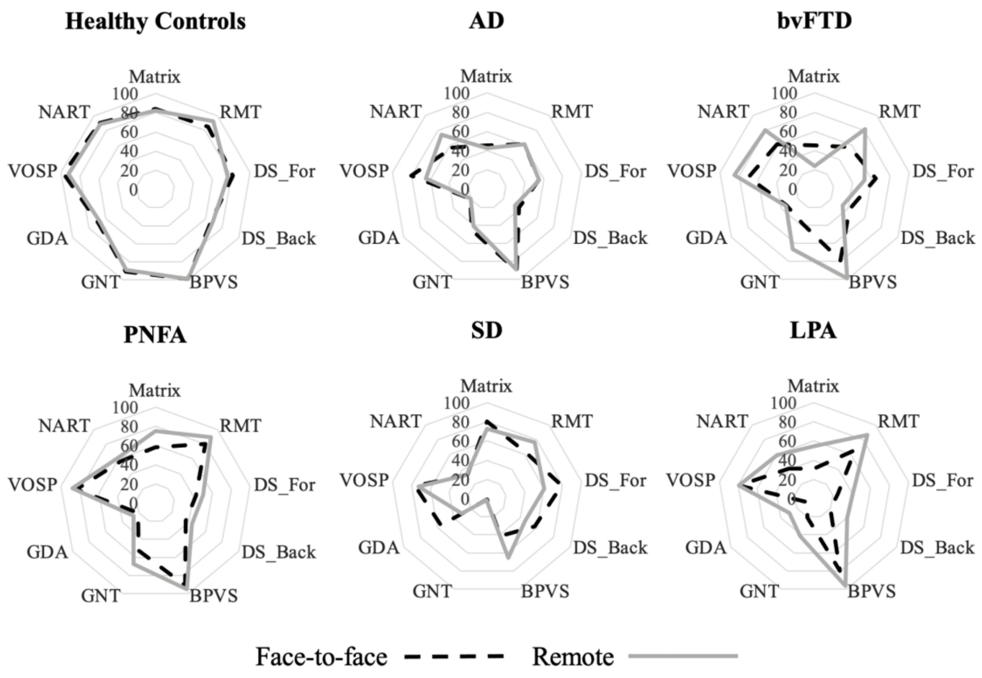

Figure 1. Radar plots of general neuropsychology battery performance, by participant group and testing environment.

Average percentage correct score (plotted on concentric lines) was calculated for each participant group for each test in the neuropsychology battery, across each testing environment. Scores for the fluency tasks were not included here as responses on these tasks cannot be evaluated as correct/incorrect. AD, patient group with typical Alzheimer's disease; BPVS, British Picture Vocabulary Scale; bvFTD, patient group with behavioural variant frontotemporal dementia; DS_For/Back, Digit Span Forwards/Backwards; GDA, Graded Difficulty Arithmetic test; GNT, Graded Naming Test; LPA, patient group with logopenic progressive aphasia; Matrix, WASI Matrix Reasoning; NART, National Adult Reading Test; PNFA, patient group with progressive nonfluent aphasia; RMT, Recognition Memory Test; SD, patient group with semantic dementia; VOSP, Visual Object Space Perception battery.

409x293mm (118 x 118 DPI)

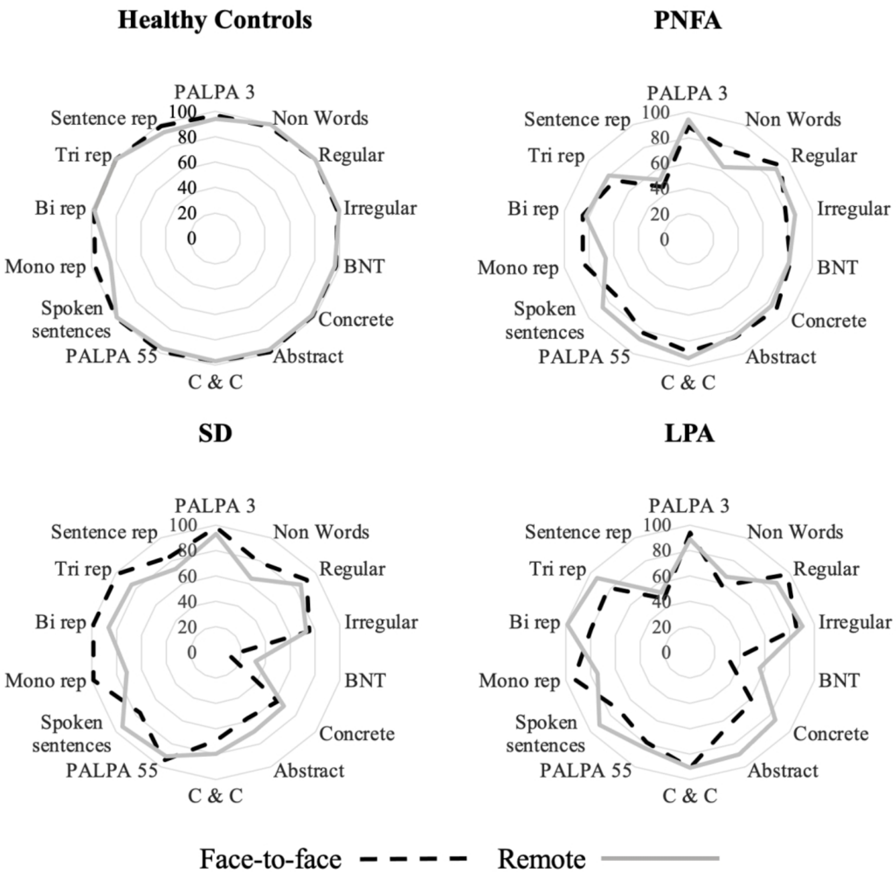

Figure 2. Radar plots of performance on neurolinguistic battery performance, by participant group and testing environment.

Average percentage score (plotted on concentric lines) was calculated for each participant group for each test in the neurolinguistic battery, across each testing environment. Abstract, abstract synonyms test; bi rep, bisyllabic single word repetition; BNT, Boston Naming Test; C & C, camel and cactus test; concrete, concrete synonyms test; irregular, irregular word reading test; LPA, patient group with logopenic progressive aphasia; mono rep, monosyllabic single word repetition test; non word, non-word reading test; PALPA, Psycholinguistic Assessment of Language Processing in Aphasia subtests; PNFA, patient group with progressive nonfluent aphasia; regular, regular word reading test; sentence rep, graded difficulty sentence repetition test; SD, patient group with semantic dementia; spoken sentences, spoken sentences test; tri rep, trisyllabic single word repetition test.

352x319mm (118 x 118 DPI)

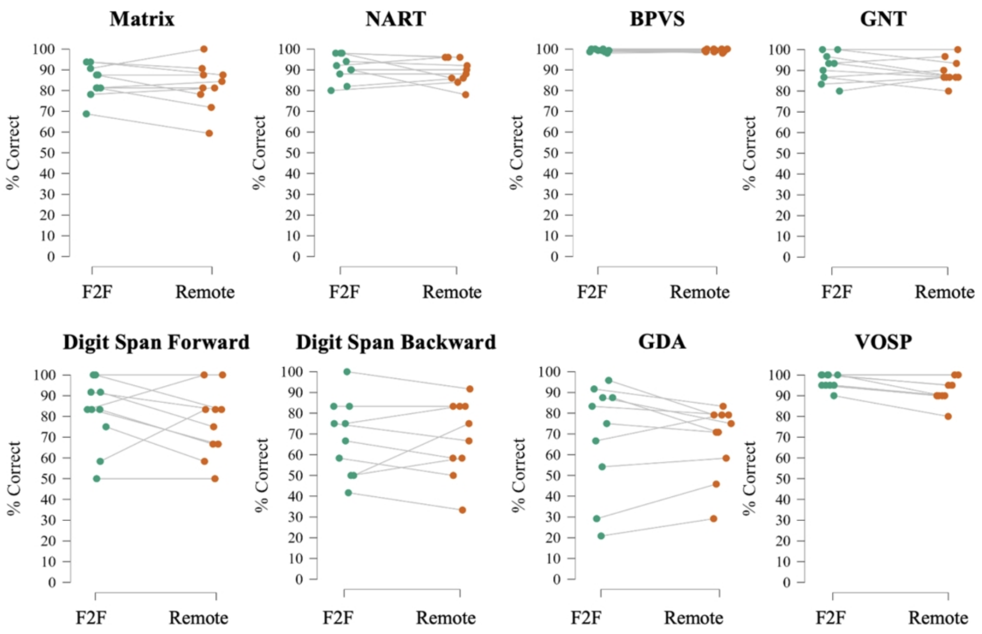

Figure 3. Performance profiles of healthy control participants on tasks in general neuropsychological battery.

Line plots showing performance profiles of individual healthy control participants on tasks in the general neuropsychological battery. BPVS, British Picture Vocabulary Scale; GDA, Graded Difficulty Arithmetic test; GNT, Graded Naming Test; Matrix, WASI Matrix Reasoning; NART, National Adult Reading Test; VOSP, Visual Object Space Perception Object Decision task.

456x302mm (118 x 118 DPI)

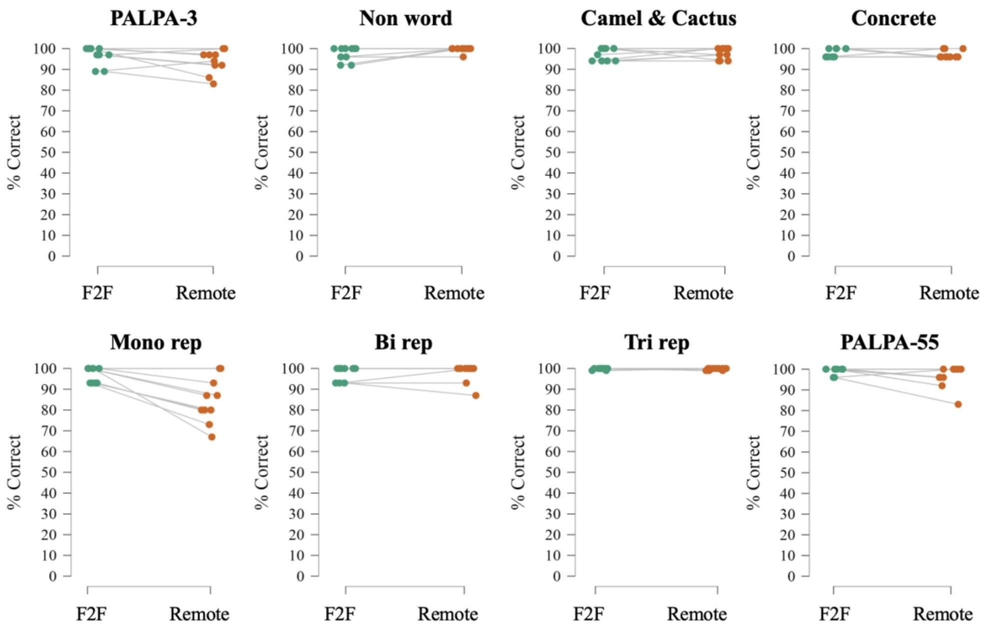

Figure 4. Performance profiles of healthy control participants on tasks in the neurolinguistic battery.

Line plots indicating percentage scores for each healthy control on representative tests from the neurolinguistic battery administered face-to-face and remotely. Scores on the trisyllabic single word repetition task were jittered slightly on the x-axis to allow for plotting as participants were uniformly at ceiling in both environments. Bi rep, bisyllabic single word repetition; BNT, Boston Naming Test; Concrete, concrete synonyms test; F2F, face-to-face; Mono rep, monosyllabic single word repetition test; Non word, non-word reading test; PALPA, Psycholinguistic Assessment of Language Processing in Aphasia; Tri rep, trisyllabic single word repetition.

452x294mm (118 x 118 DPI)

Supplementary Material: Remote versus face-to-face neuropsychological testing for dementia research: a comparative study in Alzheimer's disease, frontotemporal dementia, and healthy older individuals

by MC Requena-Komuro, J Jiang et al

Technical aspects of the set-up for remote testing

Participants were permitted to use their preferred device (tablet, laptop, or desktop computer; see Table 1). To ensure screen visibility, we did not accept the use of smartphones. Most participants (90%) listened via speakers; six participants used headphones, and device volume was set to a comfortable level by each participant or their caregiver. Remote assessments were scheduled to ensure that testing could be completed in a quiet environment with minimal distractions. Additionally, the experimenters asked that each participant's video remained turned on so the experimenter could keep track of any distractions that may be occurring (see Table 1), as well as to ensure that no additional materials were used during the tests (e.g., paper/calculator). Where a task required visual presentation, this was done by screen sharing the Microsoft Powerpoint presentation containing the scanned stimuli for that task in full screen mode. Each patient's primary caregiver was asked to be available during each research session in case of any problems with using the equipment; in practice, no major technological issues arose. The primary caregiver was also permitted to be within the room during the sessions (see Table 1), but were told explicitly not to interfere with any of the neuropsychological and neurolinguistics tests given to the patient participants.

To check basic audibility in the remote testing environment, before each remote session, participants first listened to a set of 10 sentences from the Bamford-Kowal-Bench (BKB) list[1]. These sentences have previously been validated in hearing-impaired children. The spoken sentences were delivered online using an online experiment builder, Labvanced[2]. In each trial, a spoken sentence was played to the participant via screen and sound share on Zoom and the participant was encouraged to select the last word in the sentence they had just heard from three possible options presented visually via screen share (see Figure S1). A perfect score on the final three items was required for the participant to proceed to the remote testing session proper (this allowed each participant and/or caregiver to manually adjust the volume to a comfortable level during the first seven sentences). Most participants (95%) performed at ceiling across all ten items, and no participant made an error on any of the final three items, meaning that none was rejected based on their BKB performance (see Table S5). The order of sentences was fixed across participants.

Reasons for declining participation

Of those who did not take part, 20 (23.0%) did not respond (four AD, two bvFTD, three SD, two PNFA, three LPA, six controls); six (6.9%) declined due to not being comfortable using videoconferencing technology (two LPA, four controls); two (2.3%) declined due to changing health conditions (one AD, one control); one (1.1%) bvFTD patient declined due to sensory difficulties; one (1.1%) LPA patient declined due to family reasons; two (2.3%) declined due to anxieties about research (one AD, one LPA); three (3.4%) declined due to being too impaired

For peer review only - http://bmjopen.bmj.com/site/about/guidelines.xhtml

(two bvFTD, one LPA); two (2.3%) declined due to being too busy with other activities (one SD, one PNFA); 12 (13.8%) provisionally said they were interested but we were unable to schedule a convenient time for them to take part (eight AD, three PNFA, one LPA); two (2.3%) healthy controls expressed an interest in participating but had not previously undertaken face-to-face research so were not included here; and one (1.1%) patient with atypical PPA expressed an interest in participating but was not included here as no other atypical PPA patients were recruited for the remote research.

Technical issues with remote test delivery

Three minor interruptions were recorded during administration of the remote general neuropsychological battery. On two occasions, the experimenter observed that the internet connection had slowed considerably for a brief period of time; however, this was not explicitly commented on by either participant affected (one healthy control, one bvFTD patient). The third interruption was a dog barking during an AD patient's testing session. In all cases, the trial that the interruption had occurred in was restarted, and the examiner judged that there was no penalty or benefit afforded by the interruption in any case. No interruptions were recorded during administration of the neurolinguistic battery, and no other interruptions were reported by participants.

For peer review only - http://bmjopen.bmj.com/site/about/guidelines.xhtml

Table S1. List of general neuropsychological and neurolinguistic tests delivered face-to-face that were not included in the remote battery.

Test	Reason for removal
WASI Vocabulary	Other tests from same domain already included
WASI Similarities	Other tests from same domain already included
Long Recognition Memory Test for Faces	To reduce the length of the remote battery, this was replaced with the Short Recognition Memory Test for Faces
Long Recognition Memory Test for Words	To reduce length of battery
Camden Paired Associates Learning	Other tests from same domain already included
WASI Block Design	Not feasible online as participants would not have blocks to manipulate
Stroop task	Not feasible online as too difficult for the examiner to determine the participant's target and therefore whether or not they had made an error. Also differences in colour display across participants' screens.
Trails A and B	Not feasible online as requires use of pencil and paper
WAIS-R Digit Symbol	Not feasible online as requires use of pencil and paper
Usual/Unusual views	Other tests from same domain already included
Modified Kissing and Dancing	Other tests from same domain already included
Baxter Spelling Test	Not feasible online as face-to-face task requires the participant to use pencil and paper; typing responses was not considered appropriate due to potential interference from spell-check software
Written sentences	Not feasible online as face-to-face task requires the participant to use pencil and paper; typing responses was not considered appropriate due to potential interference from spell-check software
Spatial Span Forwards	To reduce length of battery
Spatial Span Backwards	To reduce length of battery

We reduced the number of tests used in our face-to-face general neuropsychological and neurolinguistic batteries down for remote testing, reflecting a) the need to make the remote testing batteries shorter to minimise fatigue; b) impracticalities of administering certain stimuli remotely; and c) inability to adequately record participants' responses to some tasks. The Table shows the tasks that were not included in the remote batteries.

For peer review only - http://bmjopen.bmj.com/site/about/guidelines.xhtml

Table S2. Bayesian statistics comparing general neuropsychological test performance on remote vs face-to-face assessments

Test	CTL	All Patients	AD	bvFTD	SD	PNFA	LPA
General intellect							
WASI Matrix	2.56[t] (Anecdotal)	5.403 (Substantial)	3.134[t] (Substantial)	1.477[t] (Anecdotal)	1.911[t] (Anecdotal)	1.356[t] (Anecdotal)	1.441[t] (Anecdotal)
Episodic memory							
RMT Faces short	NA	3.46 (Substantial)	0.411[t] (Anecdotal)	NA	NA	NA	NA
Working memory							
DS (Reverse)	4.304[t] (Substantial)	5.28 (Substantial)	2.746[t] (Anecdotal)	Variance at 0	1.991[t] (Anecdotal)	2.659 (Anecdotal)	0.417[t] (Anecdotal)
Short-term verbal memory							
DS (Forward)	2.663[t] (Anecdotal)	4.54 [t] (Substantial)	3.467[t] (Substantial)	1.948[t] (Anecdotal)	0.799[t] (Anecdotal)	2.528[t] (Anecdotal)	1.598[t] (Anecdotal)
Language							
BPVS	4.304[t] (Substantial)	4.05 (Substantial)	3.363 (Substantial)	1.836 (Anecdotal)	1.382[t] (Anecdotal)	2.754 (Anecdotal)	1.261 (Anecdotal)
GNT	2.923[t] (Anecdotal)	4.16 (Substantial)	3.542 (Substantial)	1.407[t] (Anecdotal)	2.331 (Anecdotal)	1.68[t] (Anecdotal)	1.325[t] (Anecdotal)
NART	2.83[t] (Anecdotal)	1.99 (Anecdotal)	1.552 (Anecdotal)	1.511[t] (Anecdotal)	2.606[t] (Anecdotal)	2.52[t] (Anecdotal)	1.948[t] (Anecdotal)
Category fluency	3.767[t] (Substantial)	1.08 (Anecdotal)	3.532 (Substantial)	2.186[t] (Anecdotal)	1.715[t] (Anecdotal)	1.027 (Anecdotal)	1.29[t] (Anecdotal)
Arithmetic							
GDA Total	3.671 (Substantial)	5.30 (Substantial)	3.33 (Substantial)	2.467[t] (Anecdotal)	0.943[t] (Anecdotal)	2.483[t] (Anecdotal)	1.123[t] (Anecdotal)
Visuospatial							
VOSP	0.0404 (Substantial)	4.76 (Substantial)	0.171[t] (Substantial)	2.215 (Anecdotal)	2.478 (Anecdotal)	2.553 (Anecdotal)	2.592[t] (Anecdotal)
Executive							

For peer review only - http://bmjopen.bmj.com/site/about/guidelines.xhtml

Letter fluency	3.159[t] (Substantial)	0.59 (Anecdotal)	2.86[t] (Anecdotal)	2.426[t] (Anecdotal)	1.519[t] (Anecdotal)	0.511[t] (Anecdotal)	0.188[t] (Substantial)

A Bayes factor (BF_{01}) indicates the extent to which the null hypothesis is favoured against the alternative hypothesis (e.g., a BF_{01} value of 4 means that the obtained data are 4 times more likely under the null hypothesis than under the alternative hypothesis). A $BF_{01} > 3$ is therefore considered as substantial evidence in support of the null hypothesis; while a BF_{01} of <1/3 is considered as substantial evidence in support of the alternative hypothesis. Any values in between are categorised as 'anecdotal' evidence, equivalent to a non-significant result in inferential statistics [3]. Results are influenced by the prior (more specifically the shape of the prior influences the strength of the evidence), which can be specified be default using a Cauchy distribution, as here; the Cauchy scale set here is 1.00. The superscript [t] indicates that a parametric Bayesian test was used; else the non-parametric Mann Whitney (with 1000 iterative samples) was employed. Blue shading indicates that the alternative hypothesis (H1, i.e. there was a difference in performance across the two environments) was favoured with substantial evidence; Green shading indicates that the null hypothesis (H0; i.e. there was no difference in performance across environments) was favoured with substantial evidence. AD, patient group with typical Alzheimer's disease; BPVS, British Picture Vocabulary Scale; bvFTD, patient group with behavioural variant frontotemporal dementia; CTL, healthy control group; DS, Digit Span; F2F, face-to-face; GDA, Graded Difficulty Arithmetic test; GNT, Graded Naming Test; LPA, patient group with logopenic progressive aphasia; Matrix, WASI Matrix Reasoning; NART, National Adult Reading Test; PNFA, patient group with progressive nonfluent aphasia; RMT, Recognition Memory Test; SD, patient group with semantic dementia; VOSP, Visual Object Space Perception battery.

For peer review only

Table S3. Bayesian statistics comparing neurolinguistic test performance on remote vs face-to-face assessments

Test	CTL	All Patients	SD	PNFA	LPA
Phoneme perception					
PALPA 3	1.003 (Anecdotal)	2.918 (Anecdotal)	1.594 (Anecdotal)	2.731 (Anecdotal)	2.079 (Anecdotal)
Reading					
Non word reading	1.544 (Anecdotal)	3.239 (Substantial)	1.940[t] (Anecdotal)	2.033[t] (Anecdotal)	2.428[t] (Anecdotal)
Regular reading	Variance at 0	2.989 (Anecdotal)	1.975 (Anecdotal)	2.305 (Anecdotal)	1.923 (Anecdotal)
Irregular reading	Variance at 0	2.996 (Anecdotal)	2.256 (Anecdotal)	2.194[t] (Anecdotal)	2.005 (Anecdotal)
Naming					
BNT	3.612[t] (Substantial)	1.995 (Anecdotal)	0.584[t] (Anecdotal)	2.580 (Anecdotal)	1.490 (Anecdotal)
Semantic association					
Camel and cactus	4.039[t] (Substantial)	1.067[t] (Anecdotal)	1.524[t] (Anecdotal)	2.102 (Anecdotal)	N<2 for F2F
Word comprehension					
Concrete synonyms	2.739[t] (Anecdotal)	3.622 (Substantial)	2.009[t] (Anecdotal)	2.400[t] (Anecdotal)	1.413[t] (Anecdotal)
Abstract synonyms	1.726[t] (Anecdotal)	2.718 (Anecdotal)	0.782 (Anecdotal)	2.741 (Anecdotal)	1. 941 (Anecdotal)
Sentence comprehension					
PALPA55	0.944 (Anecdotal)	3.954 (Substantial)	2.246 (Anecdotal)	2.603 (Anecdotal)	2.110 (Anecdotal)
Speech repetition					
Monosyllabic word repetition	0.0487[t] (Substantial)	0.117 (Anecdotal)	0.477[t] (Anecdotal)	1.167[t] (Anecdotal)	1.055[t] (Anecdotal)
Bisyllabic word repetition	4.304[t] (Substantial)	3.744 (Substantial)	1.086 (Anecdotal)	2.639 (Anecdotal)	0.650[t] (Anecdotal)
Trisyllabic word repetition	Variance at 0	3.701 (Substantial)	1.339 (Anecdotal)	2.400 (Anecdotal)	1.677 (Anecdotal)
Graded difficulty sentence repetition	1.779 (Anecdotal)	2.983 (Anecdotal)	1.420[t] (Anecdotal)	2.535[t] (Anecdotal)	1.996 (Anecdotal)
Sentence construction					
Spoken	Variance at 0	1.525 (Anecdotal)	2.270 (Anecdotal)	1.960. (Anecdotal)	0.930[t] (Anecdotal)

A Bayes factor (BF_{01}) is shown for each remote vs face-to-face testing comparison. The interpretation and colour coding are as indicated in the legend to Table S2 above. BNT, Boston Naming Test; CTRL, healthy control group; F2F, face-to-face; LPA, patient group with logopenic progressive aphasia; N, number of participants per group; PALPA, Psycholinguistic Assessment of Language Processing in Aphasia subtests; PNFA, patient group with progressive nonfluent aphasia; SD, patient group with semantic dementia

6

For peer review only

Table S4. Equality of variance analyses

Test	Healthy controls	Combined patients
**General neuropsychology tasks**		
WASI Matrix	F = 0.50, p = 0.32	F* = 0.15, p = 0.70
RMT faces short	N/A	F*=0.67, p=0.42
Digit span forwards	F = 1, p = 1	F = 1.36, p = 0.41
Digit span backwards	F = 1, p = 1	F* = 0.20, p = 0.65
BPVS	F = 0.82, p = 0.77	F* = 2.56, p = 0.11
GNT	F = 1.38, p =0.64	F* = 1.15, p = 0. 29
GDA	F* = 1.14, p = 0.30	F* = 0.13, p = 0.72
VOSP	F* = 0.42, p = 0.52	F* = 0.001, p = 0.97
NART	F = 1.15, p = 0.84	F* = 1.72, p = 0.19
Letter fluency	F = 2.20, p = 0.26	F* = 1.28, p = 0.26
Category fluency	F = 2.31, p = 0.23	F* = 0.96, p = 0.33
**Neurolinguistic tasks**		
PALPA-3	F* = 0.72, p = 0.41	F* = 0.07, p = 0.79
Nonword reading	F* = 3.08, p = 0.10	F* = 1.31, p = 0.26
Regular word reading	Variance=0	F* = 0.70, p = 0.41
Irregular word reading	Variance=0	F* = 0.30, p = 0.59
BNT	F = 0.57, p = 0.42	F* = 1.37, p = 0.25
Mono-syllabic repetition	**F = 0.11, p < 0.05**	**F* = 4.89, p = 0.03**
Bi-syllabic repetition	F = 0.58, p = 0.43	F* = 0.001, p = 0.98
Tri-syllabic repetition	Variance=0	F* = 0.04, p = 0.85
Concrete synonyms	F = 1.19, p = 0.80	F* = 0.91, p = 0.35
Abstract synonyms	F = 0.29, p = 0.09	F* = 2.24, p = 0.15
PALPA-55	F* = 4.41, p=0.05	F* = 1.10, p = 0.30
Sentence repetition	Variance=0	F* = 0.48, p = 0.49
Camel and cactus	F* = 1.30, p = 0.70	**F = 4.25, p = 0.02**
Spoken sentences	Variance=0	**F* = 4.37, p = 0.046**

Results of equality of variance analyses. F-test results are reported; *indicates that Levene's test was adopted instead due to violations of the assumption of normality. **Bold** indicates that the test was significant, meaning that the assumption of homogeneity of variance was violated. The short version of the RMT faces task was not administered to healthy control participants as part of their remote testing battery, making an equality of variance test impossible here. BPVS, British Picture Vocabulary Scale; GDA, Graded Difficulty Arithmetic test; GNT, Graded Naming Test; Matrix, WASI Matrix Reasoning; NART, National Adult Reading Test; VOSP, Visual Object Space Perception Object Decision task. BNT, Boston Naming Test; PALPA, Psycholinguistic Assessment of Language Processing in Aphasia.

For peer review only

Table S5. Performance on audibility screening task by remote participant groups

	CTL	AD	bvFTD	SD	PNFA	LPA
Pre neuropsychology battery						
Average number of incorrect items (/10)	0.0 (0.0)	0.1 (0.4)	0.0 (0.0)	0.0 (0.0)	0.2 (0.4)	0.0 (0.0)
Average number of errors on last three items (/3)	0.0 (0.0)	0.0 (0.0)	0.0 (0.0)	0.0 (0.0)	0.0 (0.0)	0.0 (0.0)
Pre neurolinguistic battery						
Average number of incorrect items (/10)	0.0 (0.0)	NA	NA	0.0 (0.0)	0.4 (0.9)	0.0 (0.0)
Average number of errors on last three items (/3)	0.0 (0.0)	0.0 (0.0)	0.0 (0.0)	0.0 (0.0)	0.0 (0.0)	0.0 (0.0)

The data indicate that there were no major background listening environmental confounds nor any significant differences between participant groups (all p>0.05). AD, patient group with typical Alzheimer's disease; bvFTD, patient group with behavioural variant frontotemporal dementia; CTL, healthy control group; LPA, patient group with logopenic progressive aphasia; NA, not applicable; PNFA, patient group with progressive non-Fluent aphasia; SD, patient group with semantic dementia.

For peer review only - http://bmjopen.bmj.com/site/about/guidelines.xhtml

Figure S1. Example of basic audibility screening measure

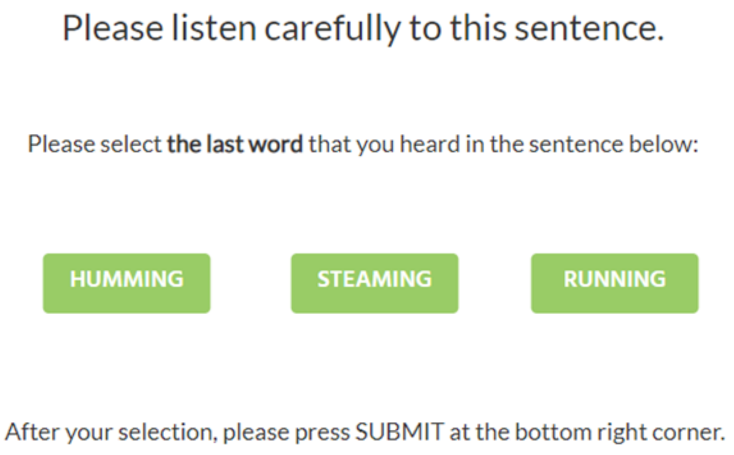

The Figure shows a Labvanced display of the BKB hearing screening measure. In this example, the sentence spoken was "The car engine is running". For each sentence, two foils were displayed alonside the target, both of which made sense in the sentence when replacing the target. One of the foils was also selected to loosely rhyme with the target word (here, "humming").

For peer review only - http://bmjopen.bmj.com/site/about/guidelines.xhtml

Figure S2. Performance profiles of patients on tasks in general neuropsychological battery.

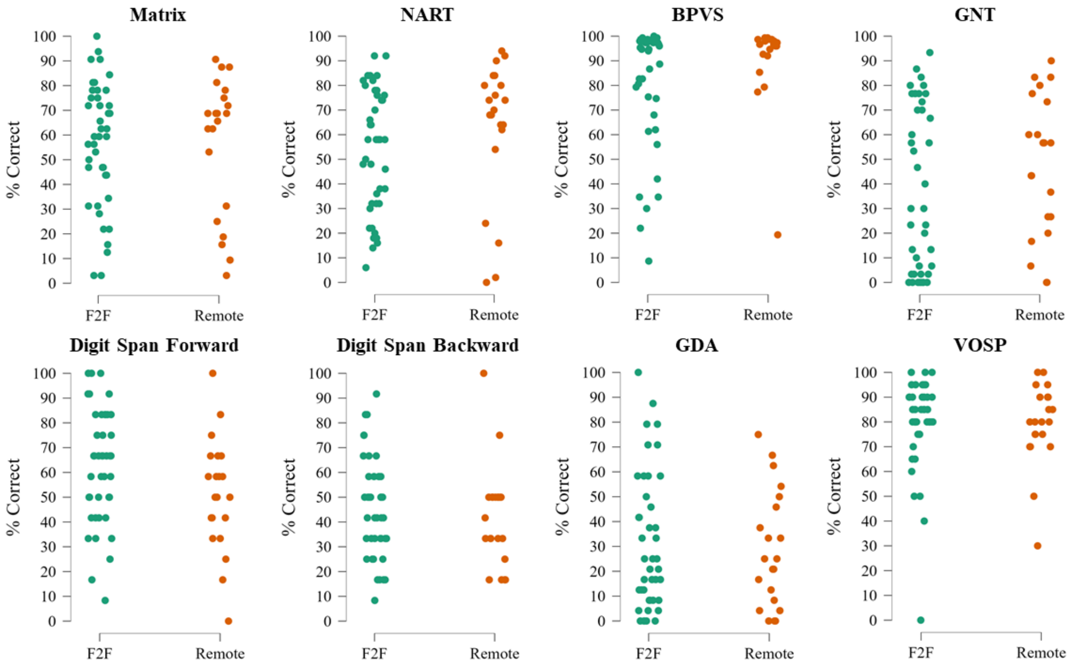

Scatter plots showing performance profiles of individual patients on tasks in the general neuropsychological battery. BPVS, British Picture Vocabulary Scale; GDA, Graded Difficulty Arithmetic test; GNT, Graded Naming Test; Matrix, WASI Matrix Reasoning; NART, National Adult Reading Test; VOSP, Visual Object Space Perception Object Decision task.

For peer review only - http://bmjopen.bmj.com/site/about/guidelines.xhtml

Figure S3. Performance profiles of patients on tasks in the neurolinguistic battery.

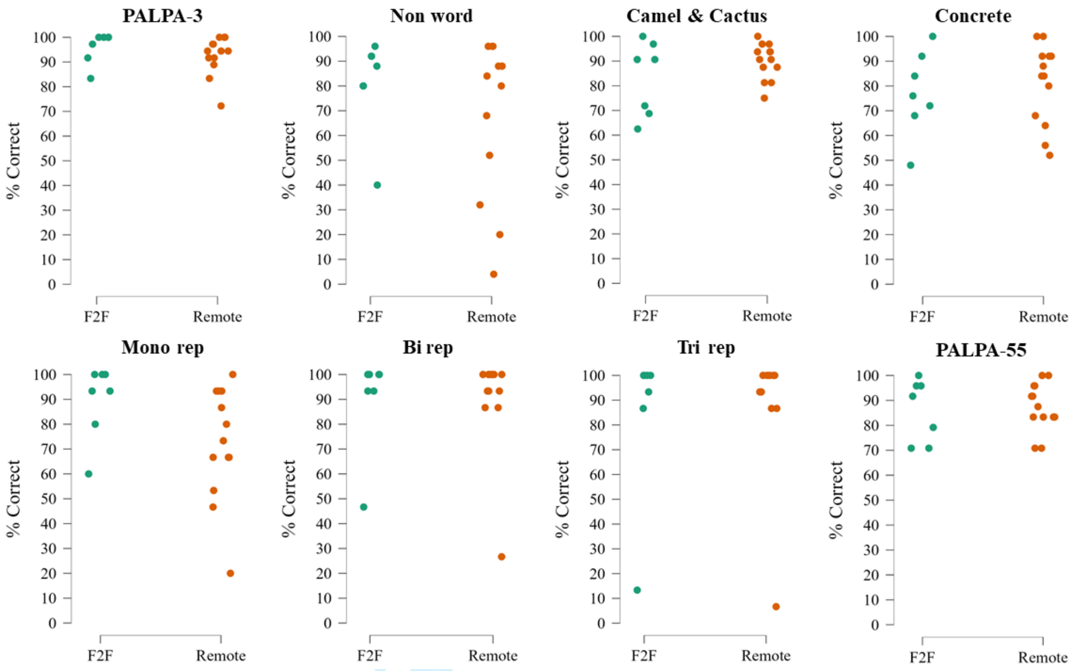

Scatter plots indicating percentage scores for each patient on representative tests from the neurolinguistic battery administered face-to-face and remotely. Bi rep, bisyllabic single word repetition; BNT, Boston Naming Test; Concrete, concrete synonyms test; F2F, face-to-face; Mono rep, monosyllabic single word repetition test; Non word, non-word reading test; PALPA, Psycholinguistic Assessment of Language Processing in Aphasia; Tri rep, trisyllabic single word repetition.

For peer review only - http://bmjopen.bmj.com/site/about/guidelines.xhtml

References

1. Bench J, Kowal Å, Bamford J. The Bkb (Bamford-Kowal-Bench) Sentence Lists for Partially-Hearing Children. *British Journal of Audiology* 1979;13(3):108-12. doi: 10.3109/03005367909078884
2. Finger H, Goeke C, Diekamp D, et al. LabVanced: A Unified JavaScript Framework for Online Studies. *International Conference on Computational Social Science* 2016
3. Ashworth M, Palikara O, Burchell E, et al. Online and Face-to-Face Performance on Two Cognitive Tasks in Children With Williams Syndrome. *Front Psychol* 2021;11:594465. doi: 10.3389/fpsyg.2020.594465

For peer review only

STROBE 2007 (v4) Statement—Checklist of items that should be included in reports of *cohort studies*

Section/Topic	Item #	Recommendation	Reported on page #
**Title and abstract**	1	(*a*) Indicate the study's design with a commonly used term in the title or the abstract	2
		(*b*) Provide in the abstract an informative and balanced summary of what was done and what was found	2
**Introduction**			
Background/rationale	2	Explain the scientific background and rationale for the investigation being reported	3
Objectives	3	State specific objectives, including any prespecified hypotheses	4
**Methods**			
Study design	4	Present key elements of study design early in the paper	4-6
Setting	5	Describe the setting, locations, and relevant dates, including periods of recruitment, exposure, follow-up, and data collection	4-6
Participants	6	(*a*) Give the eligibility criteria, and the sources and methods of selection of participants. Describe methods of follow-up	4-6
		(*b*) For matched studies, give matching criteria and number of exposed and unexposed	4
Variables	7	Clearly define all outcomes, exposures, predictors, potential confounders, and effect modifiers. Give diagnostic criteria, if applicable	4-6
Data sources/ measurement	8*	For each variable of interest, give sources of data and details of methods of assessment (measurement). Describe comparability of assessment methods if there is more than one group	*5-6*
Bias	9	Describe any efforts to address potential sources of bias	4
Study size	10	Explain how the study size was arrived at	4
Quantitative variables	11	Explain how quantitative variables were handled in the analyses. If applicable, describe which groupings were chosen and why	6-7
Statistical methods	12	(*a*) Describe all statistical methods, including those used to control for confounding	6-7
		(*b*) Describe any methods used to examine subgroups and interactions	6-7
		(*c*) Explain how missing data were addressed	6-7
		(*d*) If applicable, explain how loss to follow-up was addressed	6-7
		(*e*) Describe any sensitivity analyses	NA
**Results**			

For peer review only

Participants	13*	(a) Report numbers of individuals at each stage of study—eg numbers potentially eligible, examined for eligibility, confirmed eligible, included in the study, completing follow-up, and analysed	4
		(b) Give reasons for non-participation at each stage	4
		(c) Consider use of a flow diagram	NA
Descriptive data	14*	(a) Give characteristics of study participants (eg demographic, clinical, social) and information on exposures and potential confounders	4, 8
		(b) Indicate number of participants with missing data for each variable of interest	16
		(c) Summarise follow-up time (eg, average and total amount)	NA
Outcome data	15*	Report numbers of outcome events or summary measures over time	7-9
Main results	16	(*a*) Give unadjusted estimates and, if applicable, confounder-adjusted estimates and their precision (eg, 95% confidence interval). Make clear which confounders were adjusted for and why they were included	6-9
		(*b*) Report category boundaries when continuous variables were categorized	NA
		(*c*) If relevant, consider translating estimates of relative risk into absolute risk for a meaningful time period	NA
Other analyses	17	Report other analyses done—eg analyses of subgroups and interactions, and sensitivity analyses	7-9
**Discussion**			
Key results	18	Summarise key results with reference to study objectives	9
**Limitations**			
Interpretation	20	Give a cautious overall interpretation of results considering objectives, limitations, multiplicity of analyses, results from similar studies, and other relevant evidence	10
Generalisability	21	Discuss the generalisability (external validity) of the study results	10
**Other information**			
Funding	22	Give the source of funding and the role of the funders for the present study and, if applicable, for the original study on which the present article is based	11

*Give information separately for cases and controls in case-control studies and, if applicable, for exposed and unexposed groups in cohort and cross-sectional studies.

Note: An Explanation and Elaboration article discusses each checklist item and gives methodological background and published examples of transparent reporting. The STROBE checklist is best used in conjunction with this article (freely available on the Web sites of PLoS Medicine at http://www.plosmedicine.org/, Annals of Internal Medicine at http://www.annals.org/, and Epidemiology at http://www.epidem.com/). Information on the STROBE Initiative is available at www.strobe-statement.org.

For peer review only
